# Development of 18 Quality Control Gates for Additive Manufacturing of Error Free Patient-Specific Implants

**DOI:** 10.3390/ma12193110

**Published:** 2019-09-24

**Authors:** Daniel Martinez-Marquez, Milda Jokymaityte, Ali Mirnajafizadeh, Christopher P. Carty, David Lloyd, Rodney A. Stewart

**Affiliations:** 1 School of Engineering, Griffith University, Gold Coast, QLD 4222, Australia; daniel.martinezmarquez@griffithuni.edu.au; 2 Ortho Baltic, Kaunas 51124, Lithuania; milda.jokymaityte@orthobaltic.lt; 3 Molecular Cell Biomechanics Laboratory, University of California, Berkeley, CA 94720, USA; ali.mirnajafi@gmail.com; 4 School of Allied Health Sciences and Gold Coast Orthopaedic Research and Education Alliance, Menzies Health Institute Queensland, Griffith University, Gold Coast, QLD 4222, Australia; c.carty@griffith.edu.au (C.P.C.); david.lloyd@griffith.edu.au (D.L.); 5 Department of Orthopaedic Surgery, Queensland Children’s Hospital, Children’s Health Queensland Hospital and Health Service, Brisbane, QLD 4101, Australia

**Keywords:** additive manufacturing, patient-specific implants, quality control, 3D printing, regulations and standards

## Abstract

Unlike subtractive manufacturing technologies, additive manufacturing (AM) can fabricate complex shapes from the macro to the micro scale, thereby allowing the design of patient-specific implants following a biomimetic approach for the reconstruction of complex bone configurations. Nevertheless, factors such as high design variability and changeable customer needs are re-shaping current medical standards and quality control strategies in this sector. Such factors necessitate the urgent formulation of comprehensive AM quality control procedures. To address this need, this study explored and reported on a variety of aspects related to the production and the quality control of additively manufactured patient-specific implants in three different AM companies. The research goal was to develop an integrated quality control procedure based on the synthesis and the adaptation of the best quality control practices with the three examined companies and/or reported in literature. The study resulted in the development of an integrated quality control procedure consisting of 18 distinct gates based on the best identified industry practices and reported literature such as the Food and Drug Administration (FDA) guideline for AM medical devices and American Society for Testing and Materials (ASTM) standards, to name a few. This integrated quality control procedure for patient-specific implants seeks to prepare the AM industry for the inevitable future tightening in related medical regulations. Moreover, this study revealed some critical success factors for companies developing additively manufactured patient-specific implants, including ongoing research and development (R&D) investment, investment in advanced technologies for controlling quality, and fostering a quality improvement organizational culture.

## 1. Introduction

### 1.1. Impact of AM in the Medical Industry

Additive manufacturing (AM), generally known as 3D printing, uses technologies supported by computer-aided design (CAD) software to progressively build 3D physical models from a series of cross sections that are automatically joined together to create the final shape [1]. This 3D printing technology was pioneered in the 1980s by Charles Hull, who used a system called stereolithography (SLA) [2], and over subsequent years, a number of different systems emerged, all using the same basic principle of fusing 2D layers to create a 3D model. The existing AM processes are classified into seven main categories: binder jetting; directed energy deposition; material extrusion; material jetting; powder bed fusion; sheet lamination; and vat photopolymerization [3]. Within these AM categories, the most used AM systems in the biomedical industry are stereolithography, selective laser sintering, Inkjet 3D printing, electron beam melting, polyjet photopolymer, and fused deposition modeling [4,5], especially in orthopedics, dentistry, and maxillofacial surgery [6,7].

Appropriate materials for implantation require distinct biocompatible properties depending on implant function and location within the human body [8,9]. There are a large variety of materials used with AM to produce bone implants and scaffolds. For example, ceramic materials are used in tissue engineering for bone substitution and regeneration due to their good biocompatibility and mechanical properties [10]. Some of the most representative ceramic materials in AM for bone scaffolds applications are hydroxyapatite (HAP) and β-tricalcium phosphate (β-TCP) [11]. Ceramic materials are brittle and crack prone; however, their high wear resistance makes them the preferred choice for surface coating in joint prostheses [12]. Different polymers and bioglasses, such as collagen, chitosan, alginate, and 45S5 Bioglass^®^, have been intensely investigated for bone tissue engineering applications due to their biodegradability, easy processability, and flexibility to tailor their properties for bone regeneration [9,13,14,15]. Nevertheless, practical application of these materials for load bearing applications is limited due to their low resistance to cyclic loading, brittleness, and processability difficulties, to name a few [16,17]. Therefore, these types of materials are mainly used in industry for cranial and facial reconstruction [18,19]. Therefore, to replace hard tissue for load bearing applications, metals have been the best choice due to their mechanical properties, corrosion resistance, and biocompatibility. Most of these materials are alloys, such as 316L stainless steel (316LSS), cobalt chromium (Co-Cr), and titanium alloys [20]. Above all metallic materials, titanium alloys such as Ti-6Al-4V are currently considered as the gold standard for orthopedic applications [21]. Consequently, the majority of AM patient-specific implants currently produced in the orthopedic industry are made of Ti-6Al-4V alloy.

Unlike subtractive manufacturing technologies, AM offers several advantages in terms of product design and manufacturing environment due to its ability to build parts by joining the material layer by layer [3]. Firstly, due to the manufacturing flexibility of AM, just-in-time production becomes the natural manufacturing environment for these technologies, where a short-lead to market is needed [22,23]. This AM capability allows companies to adapt to the immediate intermittent demand of personalized products using minimal amounts of material resources to produce high-quality goods with maximum variety [24]. Secondly, AM technologies are capable of fabricating complex shapes that are not possible with the use of traditional manufacturing methods. This capability permits fabrication of hierarchical structures at the micro-scale level, allowing the manipulation of material properties to create meso-materials. In terms of implant design, this means that products can be designed with a biomimetic approach according to patient anatomy and the bone tissue mechanical properties [25]. This design freedom opens the way for AM to be used for difficult clinical scenarios where bone diseases, deformities, and trauma usually require reconstruction of bone defects with complex anatomical shapes, which is extremely difficult to perform, even for the most skilled surgeon [26]. The complex reconstruction of bone defects is possible by combining the advantages of AM with CAD and medical image technologies such as computed tomography and magnetic resonance in order to fabricate implants according to the patient’s specific anatomy, thus achieving an exact adaptation to the region of implantation. Nevertheless, factors such as high design variability and changeable customer needs of patient-specific products increase complexity in all domains, requiring shorter and more effective product development cycles with the integration of high performance processes and technologies in order to achieve reliable production systems [27,28,29]. These factors are also re-shaping manufacturing and medical standards, manufacturing management, quality control, and product lifecycle management [7,27].

### 1.2. The Quest for Comprehensive Standards for the AM Medical Industry

Standards are voluntary documents that establish specifications, procedures, and guidelines to maximize the reliability of products, services, and systems and ensure they are consistent and safe. Standards help to make better products that are compatible and able to interact with other products. Standards facilitate the implementation of technologies and speed up the product development cycle. Generally, standards stimulate innovation by accumulating, codifying, and sharing technological knowledge and experience [30] through the identification of best industry and research practices in producing better products [31].

In the medical field, companies are subjected to strict regulations that require the implementation of quality standards, such as ISO13485:2016, in order to demonstrate their ability to provide medical devices and related services that consistently meet customer requirements [32]. These standards and regulations are designed for mass production and off-the-shelf standard implants that have low variability of product characteristics. As a result, the recommended quality control methods for medical devices heavily rely on statistical techniques and regulations focused on compliance of customer requirements instead of continuous product improvement and satisfaction of customer needs [33]. Imposed priority forces companies to deliberately design their products to fit within existing approved thresholds in order to avoid seeking further time consuming approvals for minor variations [34].

The introduction of additive manufactured patient-specific products into the medical market is presenting serious challenges to regulatory bodies tasked with managing and assuring product quality and safety [35]. According to the Food and Drug Administration (FDA) [36], some of these challenges include “determining optimal characterization and assessment methods for the final finished device, as well as optimal process validation and acceptance methods for these devices”. Medical device regulatory bodies around the world are slowly introducing regulatory changes to cope with the significant technological and scientific progress in the medical area. For example, in 2017, the European Union (EU) created the Medical Device Regulation (MDR) that will come into force in 2020 to replace the current Medical Device Directive (MDD 93/42/EEC) and Active Implantable Medical Devices Directive (AIMDD 90/385/EEC) [37]. Furthermore, despite the drastic changes that MDR will impose, currently, there is no published guidance on patient-specific medical devices [38,39].

On the other hand, in December 2017, in an effort to cope with the rapid growth of 3D-printed medical devices in the market, the FDA released its first guidance document called Technical Considerations for Additive Manufactured Medical Devices: Guidance for Industry and Food and Drug Administration Staff [36]. The objective of this document is to provide a framework to evaluate AM processes by identifying different aspects of AM technologies. Therefore, the guide does not intend to establish a quality system for the manufacture of patient-specific devices produced with AM; instead, the guide emphasizes the use of current International Organization for Standardization (ISO)/American Society of Testing and Materials (ASTM) standards for additive manufacturing. ASTM and ISO are two similar organizations focused on the development of standards for a great variety of industries [40,41]. Establishing ISO/ASTM standards is a collaboration work between the two main organizations with the purpose of developing the standards for AM. 

Currently, there are a total of fifteen ISO/ASTM active standards for AM as well as approximately 119 related standards, which should be considered for the development and the manufacture of metallic patient-specific implants and of polymeric surgical guides, as presented in Table 1. To date, ISO and ASTM are actively working on fourteen new guides for designing, manufacturing, and testing methods of AM parts, as presented in Table 2. Overall, it can be said that, despite the considerable efforts to create standards for AM technologies, there is still a lack of medical regulations for medical devices produced with AM technologies [25,42,43], which is also preventing manufacturers and practitioners from adopting these technologies [44]. Therefore, as we showed in previous work, to achieve a successful industry transformation in this domain, collaborative efforts are needed to share best industry and research practices to promote the use of AM technology and to foster innovation in the medical area [45].

## 2. Purpose and Objectives

According to Gausemeier et al. [46], quality control of AM processes will be one of the most crucial technological requirements in 2020. Moreover, to overcome the worldwide need of AM regulations and patient-specific standards, a proactive collaboration between regulatory authorities, industry stakeholders, and research is required [47]. However, to create adequate standards for AM patient-specific implants first, it is important to better understand quality control and quality assurance methods currently used in this industry. As a result, there is a critical need for research that explores the medical AM industry to facilitate the development of standards and the sharing of best quality control practices. Nevertheless, to the authors’ knowledge, there is no empirical research on how different companies in the sector of AM patient-specific implants deal with product quality.

Taking this into consideration, the purpose of this study was to explore and report on a variety of aspects related to the production and the quality control of additively manufactured patient-specific implants in three different companies. These aspects are: AM technologies, quality management systems, manufacturing process performance, quality control methods, and technologies used for this purpose. Furthermore, this study aimed to propose an innovative integrated quality control flow diagram based on the best quality control practices of the studied companies. For this purpose, the FDA “Technical Considerations for Additive Manufactured Medical Devices” were followed in conjunction with current ASTM standards for AM and the results from our previous study [36].

Thus, the objectives of this study were:

1. Describe the different AM technologies used for fabrication of patient-specific implants from an industry perspective;
2. Identify current quality issues and percentages of rework and scrap in the industry of AM patient-specific implants;
3. Identify key quality control methods and technologies used during design and fabrication of AM patient-specific implants from an industry perspective;
4. Develop an integrated quality control flow diagram with gates for the design and the fabrication process of AM patient-specific implants taking into consideration best industry practices.

## 3. Material and Methods

To develop the clearest possible picture of a contemporary phenomenon within its real-life context, it was vital to perform an in-depth examination in the form of a case study, which in this case required gathering primary data from different organizations [48,49]. Moreover, where there was limited information about the topic, a qualitative approach was more suitable to capture textual data from a few selected cases [50]. Therefore, to achieve objectives 1, 2, and 3, a qualitative exploratory investigation in the form of a case study was performed following the consolidated criteria for reporting qualitative research (COREQ) [51]. Furthermore, to produce a validated managerial solution (objective 4) to this practical problem, a constructive research approach was employed [52].

### 3.1. Data Collection 

Conducting a case study in business and management research requires the gathering of primary data through interviews and questionnaires from key individuals such as managers, workers, and technical staff to extract expert knowledge about their experiences, beliefs, or opinions [48,53,54]. Semi-structured interviews are mainly used to gather qualitative data as well as when the researcher wants to delve deeply into a topic to thoroughly understand the answers provided [55]. Face-to-face interviews have the advantage of having the highest response rate in survey research [56]. Moreover, face-to-face interviews capture the most detail of both verbal and nonverbal communication and provide a space to establish rapport with participants, permitting the researcher to clarify ambiguous answers during the interview [56]. Therefore, the team selected face-to-face interviews as the main data collection method for this research. To ensure that each interview was performed under the same standards, an interview guide and a protocol were developed following the consolidated criteria for reporting qualitative research (COREQ) [51].

### 3.2. Study Selection

The criteria to select the companies for this study were based on their experience and expertise in the design and the manufacturing of medical devices using AM technologies. These included companies in the aerospace field due to their shared similarities in relation to materials used and strict quality regulations. Hence, the companies selected for this study had to comply with at least one of the following criteria: (1) companies that manufacture patient-specific implants and/or medical devices using AM; (2) companies that design patient-specific implants and/or medical devices for AM; (3) companies that manufacture aerospace parts with AM technologies; and (4) companies that design aerospace parts with AM.

It is noteworthy to mention that, currently, the use of AM in biomedical and aerospace industries is limited with a relatively small number of international companies operating in this new industry. Many of these companies are not open to researchers and open sharing of knowledge due to intellectual property concerns. This study sought to extract in-depth knowledge on AM processes and practices from companies that were open to knowledge sharing. The niche size of the biomedical AM industry and the limited number of companies willing to share in-depth information necessitated that the research team focus on a sample of comprehensive case studies. Consequently, this study is characterized by a small sample size but with in-depth and comprehensive data collection [57].

### 3.3. Data Extraction

For the data extraction of this study, the team developed a semi-structured guide and a PowerPoint presentation to be conducted in the form of face-to-face interview in the premises of each company. Moreover, a research information sheet and a consent form were developed and delivered at the beginning of each interview. The purpose of the information sheet was to provide a detailed description of this study and the type of information that would be requested from each company. The consent form described that the identity of each participant would be considered confidential and that only a de-identified summary of results would be used for presentations and publications. The interview guide was composed by constructing a set of 28 open-ended questions to guide the direction of the conversation. The interview questions were divided in six main groups. The first group was composed of 7 questions aimed to acquire participants’ and companies’ backgrounds. The remaining five groups of questions were designed to achieve objectives 1, 2, and 3. 

The types of questions asked during each interview were descriptive and structural. Descriptive questions are used to gather descriptions of things and processes in order to obtain insight or to check validity or accuracy about them [54]. In contrast, structural questions help the researcher to categorize groups of things and processes and to understand their relationships [54]. Furthermore, semi-structured interviews allow for new questions to emerge through the interview process [58], which may reveal new and different aspects of the topic [55]. Additionally, a protocol composed of 11 steps was developed to perform the semi-structured interviews. A more detailed description of the data extraction method, the protocol, and an example of the questionnaire are provided in the Supplementary Material S1.

### 3.4. Data Analysis

The data analysis was performed following within-case and cross-case analysis approaches [59]. In this study, the within-case analysis was concerned with the evaluation of the collected data as well as the reporting of the findings of each individual case study. The information obtained from each interview and visits to the manufacturing premises provided a clear understanding of the design, the fabrication, and the quality control process of each company. Following this, the cross-case analysis was performed between the technologies and the processes of the studied companies with the purpose of making a comparative analysis of their advantages and disadvantages [48] in order to later produce an integrated quality control flow diagram that contains the best practices of each company, thus achieving objective 5. For a detailed description of the data analysis method, refer to the Supplementary Material S1.

## 4. Results

A total of 10 invitations to participate in this study were sent to different companies in America, Europe, and Australia to explore the design, the manufacturing, and the quality control processes of AM medical devices produced by these companies. Three companies agreed to participate in this study. To protect participant companies’ confidentiality and anonymity, they are referred as companies A, B, and C.

A total of nine face-to-face interviews were performed between June and August 2018 with pertinent experts in AM, quality control, and implant design on a one-to-one basis at the headquarters of each company. The participants interviewed in Company A comprised the head of the additive manufacturing group, the head of the product quality control group, the quality manager, and the head of the clinical engineering research group. In the case of Company B, the director of research and development (R&D), the head of additive manufacturing, and two clinical engineers were interviewed. In Company C, the interviews were carried out with the chief technology officer and the head of R&D. The duration of each interview ranged from 90 to 120 min and was followed by a detailed tour through the manufacturing premises of each company. Moreover, at the beginning of each interview, a consent form was signed by each company participant. Nevertheless, none of the participants agreed to be voice recorded. Furthermore, a non-disclosure agreement was delivered by each company and signed by the research team members of this study to protect the companies’ confidential information shared during meetings and visits in their premises.

In the following section, a description of each company background is provided. This is followed by a description of their design, manufacturing, and quality control processes. Finally, an integrated quality control workflow diagram based on the best practices of each company is presented.

### 4.1. Companies’ Background

Company A is a medium size company that operates two business units, one in the field of design, manufacturing, sales, and distribution of patient-specific orthopedic footwear, and the other for patient specific prosthetic-orthotic devices using AM technologies. Currently, this company is producing, through the use of AM technologies, a large variety of products such as patient-specific implants, anatomical models, surgical guides, and patient-specific orthoses and prosthetic covers.

Company B is also a medium size company dedicated to product development, manufacturing, sterilization, packaging, sales, and distribution of standard and patient-specific orthopedic implants. The main headquarters of Company B are located in Europe from where its products are distributed to more than 65 countries worldwide. The types of products produced by Company B are a variety of standard and patient-specific implants, including surgical guides.

Company C is a recently incorporated American company founded to supply, manufacture, and provide the design service needed for complex metallic parts. This company produces specialized components for aerospace, medical, automobile, and oil and gas sectors solely fabricated using AM. The medical products include different types of surgical instruments and medical devices. For more details about these companies, refer to Table 3.

### 4.2. AM Systems Used in Each Company

This section discusses the different AM used by each of the companies studied. Moreover, the reasons why each company chose a specific AM are highlighted. Each of the three firms studied in this research selected a different AM system to manufacture their products. Their selection criteria were mainly based on: specific needs; budget; machine acquisition cost; operation cost; maintenance cost and downtime; technical support; fabrication accuracy and resolution; manufacturing speed.

#### 4.2.1. Company A

Company A selected three different AM systems to fabricate their products based on their advantages and disadvantages. For the fabrication of patient-specific implants made of cobalt chromium and titanium alloy Ti-6Al-4V, Company A decided to acquire an EOSINT M 280 Direct Metal Laser Sintering (DMLS) system. To fabricate surgical guides, anatomical models for surgical planning, and prosthetic covers, this company uses an EOS P 396 Selective Laser Sintering (SLS) system with polymer PA 2200 (also known as Nylon-12) as the main material. To fabricate patient-specific anatomical models for training purposes and visual means, Company A chose a 3D SYSTEMS ProJet CJP 660 Pro, which uses Binder Jetting (BJ) technology. 

The DMLS and the SLS AM systems selected by Company A use a laser to scan and fuse cross sections of compacted powder particles in a preheated bed inside a chamber filled with an atmosphere of inert gas, such as argon and nitrogen [60]. The difference between these two machines is that the EOSINT M 280 DMLS system requires a more powerful laser (400 watt) to partially melt metallic materials [61], whereas the EOS P 396 machine uses a 70 W laser to sinter thermoplastics [62].

According to the head of the additive manufacturing group of Company A, they decided to acquire a DMLS system to manufacture titanium implants firstly because the machine acquisition costs are less than other AM systems such as electron beam melting (EBM). However, other factors such as its easy maintenance, system assembly, and agreement conditions were are also heavily taken into consideration. Additionally, they can also create almost fully dense (99.8%) objects with high accuracy and resolution of small details with similar mechanical properties to common manufacturing methods, such as cast or machined parts [2]. Moreover, in terms of operational cost, the DMLS system uses less power and requires shorter downtimes compared to other AM machines. Furthermore, after building an object, it is possible to recycle the unsintered material [1]. Nevertheless, there are several disadvantages of this AM system. 

First, DMLS requires long fabrication cycles that can take several hours to a couple of days depending on the size and the number of parts [63]. Building metal parts also demands a large number of support structures that are difficult to remove, usually leaving marks on the surface [64]. Furthermore, the drastic temperature changes during the DMLS building process cause detrimental effects in the material mechanical properties, such as internal stresses, changes in material microstructure, the occurrence of pores, and anisotropic behavior along the building direction [65]. Nevertheless, these effects are counteracted with different heat treatments, such as annealing to provide the required mechanical properties for medical use [66].

In the case of the SLS system, its advantages are that it can build large and small parts for prototypes, models, and final products [67] without the need of supports [68]. Moreover, for this system, there is a wide range of semi-crystalline and amorphous polymers available [69]. Additionally, SLS builds plastic objects with less anisotropy and best mechanical properties among AM systems [68]. Nevertheless, the disadvantages of this system are that objects fabricated with SLS have a rough surface [68], and their mechanical properties may vary depending on their orientation during fabrication [70]. Furthermore, the unsintered powder has poor reusability because it suffers from thermal degradation, affecting its molecular weight [70].

Inkjet 3D Printing (3DP), also known as binder jet printing, is a low temperature (room temperature) AM technique that builds the 3D model by applying discrete droplets with the method drop on demand (DoD) that releases drops of liquid adhesive one-by-one when needed to bind powder material on a powder bed layer by layer [69]. This technology can create parts made of polymers, ceramic, metals, and wax [71,72]. However, the machine acquired by Company A uses ColorJet Printing (CJP) technology to bind a plaster-ceramic material with water-based binder. Full-color anatomical models with different textures can be created to facilitate surgical planning [73,74,75]. Other advantages of inkjet 3D printers are that they are cost effective machines that can rapidly build small and large parts without the need of supports [5,76,77]. Nonetheless, the drawbacks of using Inkjet 3D printing are that the built parts are fragile, it requires post-processing, it has large tolerances, and it produces rough surface finishes [74].

#### 4.2.2. Company B

Currently, Company “B” is using four Arcam Q10 plus EBM machines. The EBM system is similar to the SLS technology in the way that cross sections of metal powder are melted in a powder bed to build the model. However, EBM uses a 60 kW beam of electrons to fuse the powder particles inside a vacuum chamber at temperatures between 600–1000 °C [78,79]. When the model is finished, the chamber is filled with helium gas to speed up the cooling process [80].

According to the head of additive manufacturing and the director of R&D, the company decided to acquire this system for several reasons. First, with the EBM system, it is possible to fully use its volume capacity because it allows an easy stacking of parts in the Z-direction, which is not possible with other AM systems. These advantages in combination with the numerous electron beams simultaneously used in the EBM system makes this system suitable for high production rates [64]. Moreover, parts fabricated with EBM require a lower number of supports, which can easily be removed. Furthermore, due to the high temperatures inside the vacuum chamber and the controlled temperature cycles, the produced parts have very low residual stresses and distortion, creating fully dense high purity metallic parts with unique mechanical properties and microstructures that can meet and in fact exceed the ASTM standards [81]. For example, Co-Cr-Mo alloy parts that are fabricated with EBM have a higher percentage of elongation (up to 20%) compared to Co-Cr-Mo ASTM alloys when made using traditional processes [80]. 

Further, EBM produces parts with higher surface roughness [82], which are known to promote the early stages of bone healing and osseointegration of titanium implants [83,84]. However, high surface roughness has detrimental effects in material mechanical properties that reduce its fatigue resistance [85]. Other disadvantages of EBM are the costs associated with machine acquisition, maintenance, and production. Apart from its high acquisition cost, its downtimes are 50% higher than laser-based AM systems. Moreover, due to the high amount of energy required to operate the electron beam, its consumption of electricity is much higher [82]. 

Additionally, EBM has a narrow range of available materials, which are metals with sufficient electrical conductivity that can be melted. Therefore, only the cobalt chromium alloy Cr-Co ASTM F75, three titanium alloys, Ti-6Al-4V, Ti-6Al-4V ELI, and Ti Grade 2, and nickel alloy 718 are commercially available for this technology [86].

#### 4.2.3. Company C

In the case of Company C, they are producing 24,000 stainless steel parts per year for aerospace, medical, automobile, and oil and gas applications solely with AM. According to the company’s chief technology officer, this ultra-fast production rate is only possible thanks to their own in-house developed AM system. This system works in a similar way as an Inkjet 3D printer with a powder bed. However, instead of precisely building the part’s geometry layer by layer, their system uses a non-inkjet-based spray system combined with a micro CNC milling machine.

Their fabrication process starts with a spray head that locally sprays binder droplets onto the powder bed, wetting and binding the entire layer of powder particles of the target area. This process is repeated several times, layer by layer. Then, the process is followed by a 250 micron end-mill CNC, which cuts, shapes, and contours several layers at once with high precision. When the fabrication process is finished, the green specimen is extracted from the powder bed and sintered in an oven at about 1000 °C. As reported by the chief technology officer, the advantages of this AM system are that the final cost is cut by up to 80% compared to other AM systems. 

Moreover, the ultra-fast production rate allows them to mass produce and compete with traditional manufacturing methods such as machining and metal injection molding. Additionally, the produced parts have an approximate density of 99%+ with excellent mechanical properties and surface finish that exceed the Metal Powder Industries Federation (MPIF) standards MPIF 35 for metal injection molded parts. 

According to the head of R&D, this is due to the powder material that this system uses, which is a standard metal injection molding (MIM) powder. Nevertheless, the drawbacks of this technology are that there is only one material available (i.e., stainless steel 17-4PH), and that the final parts can experience deformation and shrinkage due to the sintering process. Unfortunately, more details about this technology cannot be further described due to the company’s policies. Table 4 details the production volumes and the AM machines used by each of the three studied companies. Table 5 details advantages and disadvantages of the different AM systems used by these companies.

### 4.3. Companies’ Quality Management System and Quality Control Processes

Almost every organization that produces products and services is supervised by some form of quality management system. These systems are aimed at establishing product and process confidence within given requirements and standards [90] through the use of policies, procedures, and guidelines to prevent and mitigate risks of non-conformance. The most well-known quality management system is ISO 9001, which is designed to suit most businesses to demonstrate an acceptable level of control over their processes with the aim of ensuring customer satisfaction and continuous improvement [91]. However, aerospace, medical, and automobile industries require the implementation of more specialized quality systems. For example, organizations that are involved in at least one of the stages of the life cycle of medical devices must demonstrate their ability to consistently meet both regulatory and customer requirements through ISO 13485 certification [32]. Moreover, an appropriate organizational culture is required for the development of an organization’s quality management practices towards continuous quality improvement. Organizational culture is composed of the belief and the values shared among the people in an organization that shape its policies and philosophy of managing business [92]. Therefore, organizations that aim to continuously improve the quality of their products and services require an organizational culture that encourages employees to proactively be concerned with quality, thus empowering quality in all activities [93].

During the visits to the premises of the studied companies, it was found the each of the three companies have obtained ISO 13485 certification and a variety of different standards to complement their quality management system, as presented in Table 6. For example, Companies A and B have their medical devices certified with the Council Directive 93/42/EEC, which is compulsory for standard medical devices in order to obtain the Conformité Européenne (CE) mark before they can be marketed in any country of the EU [94]. Moreover, Company A has three additional ISO certifications to cover different aspects of their business model. The first management system that this company implemented was the ISO 9001 in conjunction with the ISO 14001:2015, which is a voluntary environmental management standard aimed at reducing the environmental impact of a firm’s activities [95]. According to Company A’s quality manager, this certification allows the company to maintain a production that is sustainable and environmentally driven through the identification and the understanding of toxicological and environmental hazards as well as safety issues of their AM process and post processing activities. Additionally, this company has also implemented ISO/IEC 27001:2013 as its security management system to manage and preserve confidentiality, availability, and integrity of their information assets [96]. These assets, which include sensible digital data such as implants’ designs and patient information, are shared between the company and its clients. In the case of Company C, it recently acquired ISO 9001 certification and AS9100 certification, which is the quality system standard for the aerospace industry. The AS9100 is based on the ISO 9001, but it is enhanced with additional aerospace industry requirements in order to satisfy Department of Defense (DOD), National Aeronautics and Space Administration (NASA), and Federal Aviation Administration (FAA) quality requirements [97]. Interestingly, we noticed that all the companies studied have a strong organizational culture that encourages a proactive emphasis on quality and continuous improvement similar to total quality management.

Some of the external advantages of ISO 9000 series are higher perceived quality, competitive advantage, better documentation, quality awareness, and improvement in operating performance [98,99]. On the other hand, ISO 13485 provides a strong foundation towards meeting the minimum regulatory requirements of medical device regulatory bodies [91]. Many companies, including the three in this study, perceived the implementation of ISO quality systems as a major achievement [100,101]. However, quality improvement does not stop with ISO certification. ISO quality management certifications do not guarantee the production of high-quality products and reduction in the cost of poor-quality [102]. ISO 9001 only addresses the implementation of quality practices through conformance of the required documentation [103]. Therefore, ISO quality management certification is just the first step towards more advanced quality systems such as total quality management and Lean Six Sigma [104,105,106].

To demonstrate each companies’ quality control processes being implemented, a thorough site visit was undertaken through their premises. In these site visits, the team focused on the identification of quality control gates and key quality control technologies used in each gate. Quality control gates are go/no-go checkpoints designed to facilitate the detection of quality issues by measuring and monitoring products quality. Thus, in quality control gates, it is decided whether a product can continue or not thought the production chain. Moreover, it was found that the total number of quality control gates differed between the three companies. The differences in the number of quality control gates and the technologies used could be the result of their experience, competitive strategy, available budget to invest in different technologies, and their interpretation of what is needed to fulfill current regulations. A summary of quality control gates, technologies, and quality management systems is presented in Table 6.

### 4.4. Production Process Performance

Process performance evaluation is vital for future financial success. It allows the evaluation of the performance of manufacturing and organizational processes to achieve optimum business performance benchmarks [107]. Six Sigma methodology uses the Sigma quality level to measure the performance of a process. This is done by identifying the capability of the process to accomplish defect-free products [108], thus allowing the comparison of the performance of different processes irrespective of their nature [107]. 

The term Sigma (σ) refers to the number of standard deviations from the mean, where the products of a process with a normal distribution curve fall within specification limits [109]. However, Six Sigma assumes that, for long-term performance, the process mean has a 1.5 Sigma distribution shift in either specification limit [110]. Taking this into consideration, a process with a Six Sigma level means that 3.4 defects are produced per million opportunities (DPMO), giving a yield of 99.99966%. Most companies have a Sigma quality level between 2σ and 3.3σ (yield of 93.319%) [101,110,111,112,113]. Whereas, world-class companies usually operate between 4σ and 5σ levels [114,115], having between 6000 to 230 DPMO, respectively.

Some of the most commonly used Six Sigma metrics are percentage of defective products and scrap [100,101]. During the interview with the quality managers of the three companies studied, we obtained data in relation to the total number of defective additively manufactured products that each company is producing per year. For patient-specific implants, quality characteristics such as geometry and mechanical properties are targeted to nominal values with upper and lower specification limits determined by the clinical engineer, the surgeon, and specific standards. In this industry, if the final product has characteristics outside of the tolerance limits, the final product is considered scrap. 

Using this defect information provided with Equations (1) and (2), the Sigma process performance and the DPMO of each company was determined (Table 7). Equation (1) calculates the Sigma process performance in an Excel spreadsheet using NORMSINV, which is the inverse of the standard normal cumulative distribution [108]. The DPMO is determined by dividing the number of defective products by the total opportunities (number of components in a batch) and multiplying the results by one million [108].

(1) Sigmaprocessperformance=NORMSINV(probability)

$$Sigmaprocessperformance=NORMSINV\left(1-\left(Defects/10^{6}\right)\right)+1.5$$

(2) $$DPMO=\left[Defects/Opportunities\right]\times 10^{6}$$

**Figure 1:**
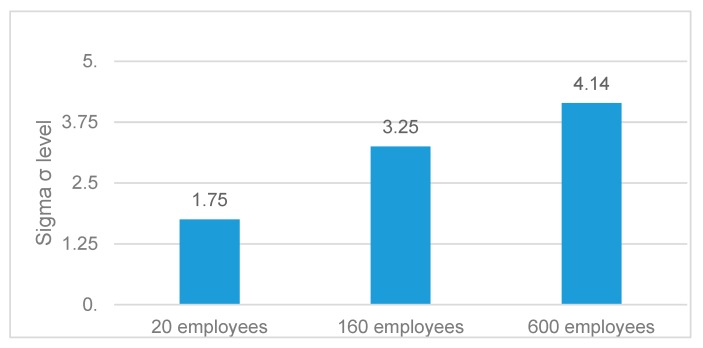
Comparative analysis of the Sigma production process performance of the studied companies against years of experience and firm size.

According to Table 7, the company with the highest Sigma level was Company B with a 4.14σ, followed by Companies A (3.25σ) and C (1.75σ). As shown in Figure 1, the Sigma process performance of the studied companies follows an increasing trend in relation to their size and years of experience in the medical device industry. The level of process performance of these two companies may look low compared with the 4σ and the 5σ levels of world-class companies. However, it must be considered that these are young companies in the market of orthopedic implants with very short performance track records [116]. 

Moreover, Companies A and B have a very small production rate to provide statistical confidence with these results. Furthermore, it should be noted that, for all companies studied, they have excellent systems in place for ensuring that no defective products are released to the market, meaning that none of these companies have had products recalls.

### 4.5. Integrated Quality Control Flow Diagram

Multistage manufacturing systems such as those used to produce patient-specific implants are composed by multiple production processes that require a delicate coordination to obtain the final product. The overall performance of these types of systems depends on the accumulated performance of their stages [117]. Therefore, error propagation is the major contributor to suboptimal overall system performance of multistage manufacturing systems [118]. According to Hrgarek [114], the cost of fixing an error increases exponentially when it moves forward through the product development cycle and the production chain [114]. This cost is even higher in the medical industry, because a defective product can represent a life threatening risk and lead to product recalls with serious financial implications such as liability costs and market capitalization loss due to negative brand image [114,119,120,121].

Quality control is composed of several processes designed to effectively monitor and prevent quality issues in order to help achieve the necessary process performance and product quality standards [122]. More recently, we identified 85 main causes that lead to non-conformance quality through design and manufacturing processes of patient-specific implants [123]. These potential risks of non-quality conformance are mainly caused by the novelty of AM technologies, product geometrical complexity, material properties, and the great variability of customers’ needs.

Controlling the quality of this type of product is a difficult task. This is due to fact that patient-specific implants are one-off design products that require higher quality standards than traditional implants, leaving no space for uncertainty. Moreover, the large variation of product characteristics in the design of patient-specific implants increases the probability of human errors due to the decreasing learning from repetitive operations [118]. Therefore, the quality control activities for the design and the fabrication of patient-specific implants should take place in the most sensible activities. This is not a rare practice in many discrete manufacturing processes, where total inspections at each intermediate operation are commonly performed [117].

To overcome some of these challenges, this study explored the quality control methods employed within three different companies to select the best quality control practices and propose an integrated quality control flow diagram for patient-specific implants. The selection of best practices was based on the quality performance of each company, including their internal quality management culture, and technologies used. The integrated quality control flow diagram was also developed taking into account the FDA guideline, “Technical Considerations for Additive Manufactured Medical Devices” [36] and ASTM standards, including the following assumptions:

- Mass production with AM is performed;
- The biocompatibility assessment was previously performed following the ISO 10993 standard;
- The aim is to achieve the highest production performance and customer quality ratings, pursuing the 6σ rating;
- Defective products are unacceptable due to the potential high risks that they represent to the company, the customer, and the patient;
- Missed flaws in the final product have serious consequences to the patient ranging from injury to fatality;
- Product external failure costs and penalty costs are much higher than a quality inspection cost. Therefore, they should be avoided in any instance;
- The scrap and rework costs and penalty risks should stay at a minimum level;
- The company employees should embrace a proactive quality culture similar to the one promoted in total quality management;
- Inspections are only performed by highly qualified personnel;
- If defects are not detected in a quality control gate, they should be detected in the following gate;
- The minimum quality management system in place should be ISO 13485;
- A detailed risk identification and a failure mode analysis should be previously performed.

**Figure 2:**
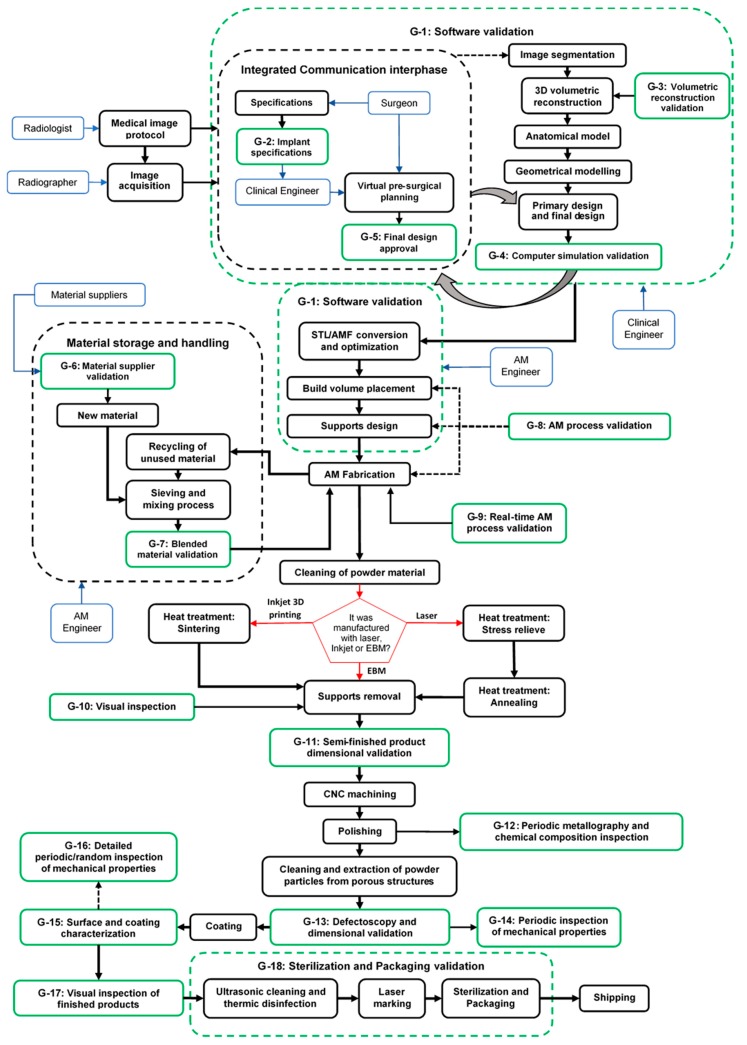
Integrated quality control workflow chart with the 18 gates for the design and fabrication of patient-specific implants by AM. The meanings of the operators in the chart are as follows: black solid outlined boxes = processes; black dash outlined boxes = overarching processes; green solid outlined boxes = quality control gates; green dash outlined boxes = overarching quality control gates; red pentagonal boxes = decision gate; blue boxes = experts/staff; solid arrows = on-line processes; dashed arrow = off-line processes.

The proposed quality control workflow diagram presented in Figure 2 considers the entire design and production cycle of patient-specific implants. It focuses on preventive quality control activities from a conservative approach. This integrated quality control flow diagram is composed of 18 go/no-go quality control gates that take place before, during, or after the most sensible processes and activities depending on their criticality and availability of quality control technologies. Go/no-go gates mean that the corresponding product quality attributes at each stage must be satisfied in order to continue to the next process [122]. Each quality control gate must have its corresponding product validation documentation containing checklists and control diagrams to track/trace product quality variations at each stage.

According to the Pareto principle, decisions made during product planning and design phases are responsible for approximately 80% of the product final costs [124,125]. Therefore, 28% of the proposed quality control gates of this study were strategically allocated in the product design phase, which could be divided into four different sub-phases: (1) information design phase; (2) conceptual design phase; (3) preliminary design phase; (4) detailed design phase [126]. From all of the 18 proposed quality control gates, 56% were on-line inspections. These inspections are part of the flow process of the design and the production line [127]. They are performed during production to catch quality variations caused by careless workers, maladjusted and uncalibrated machines, and environmental conditions [128]. This type of inspection is also aimed to control and ensure that the quality requirements of incoming materials and semi-finished/finished products are met before a value-adding operation is undertaken [128]. The remaining 44% of the quality control gates corresponded to off-line inspections performed by specialized quality inspectors. These are more detailed and time-consuming inspections that interrupt the process flow. However, they are more effective than on-line inspections [127]. Table 8 presents a brief description of all the 18 quality control gates (i.e., G-1 to G-18) of the herein developed integrated quality control workflow diagram shown in Figure 2 with their required technologies and tools. 

Additionally, a detailed description of each quality control gate is provided in a comprehensive **practitioner companion guide** to this paper provided in Supplementary Material S2.

## 5. Discussion

The different AM technologies for metal 3D printing presented in this study have proven to be ideal for the fabrication of patient-specific implants. The advantages and the disadvantages of each system heavily rely on the needs, the budget, and the market strategies of each company. For example, DMLS systems have lower acquisition cost and can fabricate higher resolution parts than EBM systems. On the other hand, EBM systems have a better monitoring system and much higher production rate than DMLS. Furthermore, the patented AM system of Company C is the only AM system that can truly mass produce metallic components, making this system the best competitor for mass production of medical devices. Regarding quality control technologies for the design and the fabrication of patient-specific implants, it can be said that there are several key technologies that are currently applied by some companies to further improve the production quality of these products. Firstly, a concurrent engineering online communication interphase is vital not only to facilitate the communication between the clinical engineer and the surgeon but also to support real time information exchange that reduces the risks of miscommunication, helping to easily identify potential issues during the implant design process.

The second key technology is a real time monitoring system for AM that can accurately monitor and control the AM production process. These real time monitoring systems are the first steps in quality control of AM machines. The last vital technology for quality control of patient specific implants is the combination of contact coordinate measurement machine, 3D laser scanner, and micro CT scanner for an accurate dimensional analysis and defectoscopy of metallic components. Nevertheless, to mass produce complex and high-performance metallic components with reliable characteristics with AM, more advanced AM machines are needed. These types of AM need to be completely integrated with micro-CT scanners—infrared cameras with smarter in-line monitoring systems—to fully control the manufacturing process in real time and achieve zero defects rates.

ISO quality management system certifications such as ISO 9001 and ISO 13485 as well as medical device registrations have proven to be great competitive advantages for many companies [129]. They can also be considered as the starting point towards more specialized quality management systems such Six Sigma, Lean Six Sigma, and total quality management [101,113]. Aspiring to world-class quality standards and process performance requires great organizational efforts and investment in training and advanced technologies [101,113]. For AM practices to achieve similar process performance levels as traditional manufacturing, comprehensive standards and well-tailored quality management systems are needed [118]. In the current environment, companies may perceive that the journey to world-class quality is long and undefined; nonetheless, there are a number of companies who are venturing into this unexplored territory with promising results.

With the explosion of AM technologies, companies such as the ones studied in this research are rapidly adapting and learning to take the great opportunities that AM is offering to the medical device industry. For this purpose, these companies are employing a variety of different strategies and approaches that are providing insight towards high quality AM products. There are several lessons learned in this research regarding process performance of AM of patient-specific implants. The first lesson is that R&D oriented companies are more prepared to apply innovative technical advances and can react more rapidly against the innovations of competitors. In this way, corporate emphasis on R&D leads towards higher levels of performance [130]. This can be clearly reflected by Companies A and B, who were able to develop and apply their own technology to extract powder particles from lattice structures, achieving results well surpassing the regulatory allowance.

Secondly, a company’s knowledge and expertise are some of the main factors for competitive advantage [131]. When a company ventures into a new market that is closely related to their core business, the new success factors that it faces are similar to the original challenges [132]. However, this situation can only positively impact corporate performance if the firm is able to rely on its previous strengths and adapt to overcome the new challenges [130,132]. For example, Company B has successfully integrated their 30 years of experience, human resources, and quality control technologies to control the production of patient-specific implants.

Finally, in emerging markets, companies have the opportunity to obtain competitive advantage by aligning their efforts and strategies towards new external opportunities [133]. In these circumstances, a competitive advantage can be created by adding exclusive, valuable resources that rivals are unable to replicate [134]. New venture companies are key players in the development of high-tech industry [135], which can give to them significant competitive advantage [136]. In the case of Company C, their in-house developed AM system provides this company a significant technological competitive advantage in terms of production capacity. The production capacity of the AM system used by Company C allows them to mass produce additively manufactured components at a rate rarely seen in the AM industry. This strong manufacturing capability also allows Company C to aggressively compete in the market with a much lower price (up to 80% less). Another strategy to compete in the AM industry is the manufacture of components for different markets to increase profitability without affecting production costs [136]. This strategy can be seen in Company C, which manufactures products for a variety of sectors such as aerospace, automobile, oil and gas, medical, and dental. Early entry in a new market can lead to high levels of long-term performance [130]. Nevertheless, one of the main important challenges for new ventures is to build long-term performance, which requires acquisition of knowledge, experience, manufacturing assets, and tangible and intangible resources [136]. Therefore, production performance improvements within Company C can be obtained by gaining further AM production experience and capital to invest in R&D and high-tech quality control technologies.

Meeting current regulations and standards is not enough to register and market high quality patient-specific medical devices. Knowing that the future changes in the medical device regulations will make more stringent the requirements to register and commercialize patient-specific implants, it is vital that companies in this sector are better prepared. It is already known that regulatory changes will force manufacturers and designers of patient-specific medical devices to provide sufficient clinical data and clinical evaluation before the registration of a device in order demonstrate its clinical performance and benefits. However, following traditional design approaches and trial-and-error studies, it is not feasible to clinically test every patient-specific implant that is produced due to the large number of clinical trials and costs involved during this process. Moreover, it is not possible to fully demonstrate the benefits of patient-specific implants if current design approaches do not account for patient specific joint contact forces produced during real-life activities.

To adequately address these challenges, we propose the combination of the Quality by Design (QbD) system with a 4D implant design approach, presented in our previous work [123], in conjunction with the 18 integrated quality control gates formulated in this study. The QbD system allows practitioners to systematically design, test, and produce reliable products with minimal clinical trials and costs. This is possible because using the QbD system can result in a 90% reduction in required experimental runs while providing a deep understanding of a product, as already proven in the pharmaceutical sector [123]. On the other hand, the 4D implant design approach can help to validate patient-specific implants with patient-specific computational neuromusculoskeletal (NMS) predictions and multiscale finite element analysis (MFEA), reducing the number of mechanical and in vivo tests. 

This 4D implant design approach can accelerate the development process of patient specific implants and simultaneously improve their reliability before clinical testing. Finally, the 18 integrated quality control gates proposed in this study can be used as a starting point to further improve quality control strategies in the sector of additively manufactured patient-specific implants. These 18 integrated quality control gates combine the best proven quality control practices from three companies manufacturing patient-specific implants as well as best-practice guidance outlined in the literature, making the proposed 18 quality control gates of great value to this industry, new ventures, and research groups. Readers are referred to the **practitioner companion guide** provided in the Supplementary Material S2 for a complete description of the developed procedure.

For mass production of AM standard components and bespoke components, the proposed quality control gates can help to reduce the potential costs related to defective products. Obviously, these savings must be higher than the inspection costs, because unnecessary inspections and longer inspection times can force the inspection capacity to an increment of inspection errors and excessive costs [137]. However, to reduce inspection time without decreasing the performance of the process, a more detailed study is required. Hence, future studies should focus on time and cost-efficient AM inspection processes in combination with in-line quality control technologies.

The limitation of this study was the small sample size of studied companies. However, this is common in qualitative research, which is characterized by small samples but detailed and extensive work [57]. Moreover, the openness and the knowledge sharing of the studied companies gave us an accurate snapshot of the technologies, the manufacturing processes, and the quality control methods used by them. This allowed us to acquire valuable detailed information to perform a comprehensive analysis and to develop an integrated quality control workflow. Therefore, the results of this study are the starting point to further improve quality control strategies in the sector of patient-specific implants and to help this industry be properly prepared for future changes in medical regulations. Overall, this study can be used as the foundation for future studies in engineering quality management of additive manufactured patient-specific implants. 

Consequently, in future research, the authors will work on a dedicated paper that will be focused on complementing the developed quality control workflow by identifying the most critical quality risks and their corresponding corrective and preventive actions in the form of a Failure Mode Effects, and Critical Analysis (FMECA). Moreover, future work in the field of engineering management should focus more on AM industry case studies in order to share best industry practices, which are necessary to fully leverage the great advantages of AM. Furthermore, another important area to consider is bioprinting, which is a rapidly growing field of research and development [138]. Bioprinting is a promising research field aimed towards the fabrication of complex biological constructs of living tissues, such as muscles and organs [139]. 

Despite the fact that bioprinting is not yet widespread at a commercial scale [140], several efforts have been made for its standardization [141,142]. Nevertheless, there is a significant need for future research to focus on the development of quality control technologies and strategies as well as process validation methods for bioprinting [143] in order to facilitate its clinical testing and foster its adoption and standardization.

## 6. Conclusions

This study explored a variety of aspects related to the production and the quality control of additively manufactured patient-specific implants in three different companies. Companies operating in this market sector should adhere to several critical success factors identified in this study, including continuous investment in R&D, investment in advanced technologies for quality control, and fostering a quality improvement organizational culture. Furthermore, in time, companies should embrace more advanced quality management systems such as Six Sigma and TQM.

This study developed an innovative quality control workflow composed of 18 gates. This quality control workflow comprises the best practices adopted by the studied companies, the FDA guideline for AM medical devices, and ASTM standards. This integrated quality control workflow represents a starting point to further improve quality control strategies in the sector of patient-specific implants and to help this industry for future changes in medical device regulations. Implementation of the developed quality control procedure outlined herein should reduce the propagation of quality issues through the product development cycle and production chain, thus minimizing the risk of product recalls and ultimately leading to heightened levels of confidence with AM patient-specific implants. Nevertheless, having a comprehensive quality workflow does not help to improve the yield or reduce the defect rates in production. 

Manufacturing processes must be able to produce products within specification limits, otherwise, there will be defective products that would eventually increase costs, prices, and customer dissatisfaction. However, due to the infancy of the AM industry for patient-specific medical products, there is presently a low level of maturity with respect to quality control technologies, comprehensive standards, and a tailored quality management system. From the results of this study, it can be said that much more has to be done to improve AM machines in order to achieve Six Sigma production levels. 

For this purpose, further developments are necessary to produce faster and more accurate AM machines coupled with smarter and more advanced real time quality control technologies. Therefore, the journey for AM to achieve a Six Sigma level process has not yet been well defined and may take some time to be realized. It is important to remember that the success of world-class companies relies not only on their products but also on the use of advanced quality management systems, decades of experience, and a strong collaboration with other industries towards standardization. 

In order to accelerate the maturity of the AM industry and derive greater quality outcomes for its products, there is a need to review and share industry best-practices, as has been conducted in this study.

## Figures and Tables

**Table 1:** Existing ISO/ASTM active standards for additive manufacturing relevant to patient-specific implants and surgical guides.

Standard Designation Code	Standard	Last Revision Date
ISO/ASTM 52900	Standard Terminology for Additive Manufacturing (AM)—General Principles—Terminology	2015
ISO/ASTM 52901	Standard Guide for Additive Manufacturing—General Principles—Requirements for Purchased AM Parts	2016
ISO/ASTM 52910	Additive Manufacturing—Design Requirements, Guidelines, and Recommendations	2018
ISO/ASTM 52915	Standard Specification for Additive Manufacturing File Format (AMF) Version 1.2	2016
ISO/ASTM 52921	Standard Terminology for Additive Manufacturing—Coordinate Systems and Test Methodologies	2013
ASTM F2924	Standard Specification for Additive Manufacturing Titanium-6 Aluminum-4 Vanadium with Powder Bed Fusion	2014
ASTM F2971	Standard Practice for Reporting Data for Test Specimens Prepared by Additive Manufacturing	2013
ASTM F3049	Standard Guide for Characterizing Properties of Metal Powders Used for Additive Manufacturing Processes	2014
ASTM F3001	Standard Specification for Additive Manufacturing Titanium-6 Aluminum-4 Vanadium ELI (Extra Low Interstitial) with Powder Bed Fusion	2014
ASTM F3091	Standard Specification for Powder Bed Fusion of Plastic Materials	2014
ASTM F3122	Standard Guide for Evaluating Mechanical Properties of Metal Materials Made via Additive Manufacturing Processes	2014
ASTM F3213	Standard for Additive Manufacturing—Finished Part Properties—Standard Specification for Cobalt-28 Chromium-6 Molybdenum via Powder Bed Fusion	2017
ASTM F3301	Standard for Additive Manufacturing–Post Processing Methods–Standard Specification for Thermal Post-Processing Metal Parts Made Via Powder Bed Fusion1,2	2018
ASTM F3302	Standard for Additive Manufacturing—Finished Part Properties—Standard Specification for Titanium Alloys via Powder Bed Fusion	2018
ASTM F3303	Standard for Additive Manufacturing—Process Characteristics and Performance: Practice for Metal Powder Bed Fusion Process to Meet Critical Applications	2018

**Table 2:** Work in process of ISO/ASTM new guides for designing, manufacturing, and testing methods of AM parts.

Draft Number	Standard
WK64190	New Guide for Additive Manufacturing Design—Decision Guide
WK49229	New Guide for Orientation and Location Dependence Mechanical Properties for Metal Additive Manufacturing
WK62190	New Specification for Additive Manufacturing Feedstock Materials Technical Specifications on Metal Powder
WK55610	New Test Methods for the Characterization of Powder Flow Properties for Additive Manufacturing Applications
WK62867	New Guide for Additive Manufacturing—General Principles—Guide for Design for Material Extrusion Processes
WK62946	New Guide for Additive Manufacturing—General Principles—Guide for Design for Directed Energy Deposition Processes
WK60265	New Guide for Assessing the Removal of Additive Manufacturing Residues in Medical Devices Fabricated by Powder Bed Fusion
WK58219	New Guide for Additive Manufacturing—Feedstock Materials-Creating Feedstock Specifications for Metal Powder Bed Fusion
WK65420	New Specification for Additive Manufacturing Qualification Principles for Equipment—Standard Guidelines Laser Powder Bed Fusion (L-PBF) for Metal
WK60942	New Test Method for Additive Manufacturing—General Principles—Effective Shear Properties for Ordered Cellular Additively Manufactured (AM) Materials
WK60943	New Test Method for Additive Manufacturing—General Principles—Effective Tensile Properties for Ordered Cellular Additively Manufactured (AM) Materials
WK60941	New Test Method for Additive Manufacturing—General Principles—Effective Compressive Properties for Ordered Cellular Additively Manufactured (AM) Materials
WK62417	Revision of F3301—18 Standard for Additive Manufacturing—Post Processing Methods—Standard Specification for Thermal Post-Processing Metal Parts Made Via Powder Bed Fusion
WK58220	New Guide for Additive Manufacturing—Process Characteristics and Performance -Standard Guidance for Specifying Gases and Nitrogen Generators Used with Metal Powder Bed Fusion Machines

**Table 3:** Summary of companies’ profile.

Company	Research Method	Experts Interviewed	Company Location	Company Age	Number of Employees	Years in the AM Market	Type of Products Produced with AM and Traditional Manufacturing	Total Production of Products Per Year with AM and Traditional Manufacturing
A	Visit to the company’s headquarters and face to face interviews	Head of Additive Manufacturing Group Head of Product Quality Control Group Quality Manager Head of Clinical Engineering Research group	Europe	18 years	160	6 years	**Manufactured with AM:** Prosthetics and orthotics Orthopedic footwear. Standard implantable medical devices. Patient-specific implants. **Not manufactured with AM:** Prosthetics and orthotics.	20,000 units per year
B	Visit to the company’s headquarters and face to face interviews	Director of Research and Development (R&D) Head of Additive Manufacturing Two Clinical Engineers	Europe	30 years	600	5 years	**Manufactured with AM:** Standard implantable medical devices. **Not manufactured with AM:** Standard implantable medical devices. Patient-specific implants.	820,000 units per year
C	Video conference and interviews	Chief Technology Officer Head of R&D	America	3 years	20	3 years	**Manufactured with AM:** Standard implantable medical devices. Patient-specific implants.	24,000 units per year

**Table 4:** Summary of AM production rates and the technologies used by Companies A, B, and C.

Company	AM Technology Used	Number of Machines	Total AM Production Per Year
A	EOSINT M 280 Direct Metal Laser Sintering system Material used: Cobalt chromium and Ti-6Al-4V	1	50 Class IIb-III patient-specific implants and unspecified number of other surgical and dental products
	EOS P 396 Selective Laser Sintering system Material used: polymer PA 2200 (also known as Nylon-12)	1	100 patient-specific surgical guides and unspecified number of other products
	3D SYSTEMS CJP ProJet 660 Pro Material used: VisiJet^®^ PXL Core	1	Unspecified number of anatomical models and other products
B	Arcam Q10plus electron beam melting (EBM) system Material used: Ti-6Al-4V	4	500 Class IIb-III patient-specific implants
C	In-house developed AM system for serial production: Material used: Stainless steel	4	24,000 parts for medical and dental applications, aerospace, automobile, oil and gas

**Table 5:** Advantages and disadvantages of the AM machines for production of metallic parts used by the studied companies.

AM System	Advantages	Disadvantages	References
**EOSINT M 280 Direct Metal Laser Sintering (DMLS)**	Moderate production rate High accuracy and details (layer thickness 20–40 µm) Fully dense parts after heat treatments Good static mechanical properties Finer grain size Produces pars with medium surface roughness that improves biological performance Requires heat treatments Metals: AlSi10Mg, Cobalt-Chrome MP1, Cobalt-Chrome SP2, Maraging-Steel MS1, Nickel Alloy HX, Nickel Alloy IN625, Nickel Alloy IN718, Stainless Steel 17-4PH, Stainless Steel CX, Stainless Steel GP1, Stainless Steel 316L, Ti-6Al-4V, Ti-6Al-4V ELI, TiCP grade 2, and Tungsten W1 Low maintenance time	High energy consumption Long building cycles Need of building supports, which are difficult to remove Grainy surface Difficult part cleaning Parts require heat treatment to release internal stresses Needs high quality powder spec, only supplied by machine brand	[1,4,63,66,76,77,87,88]
**Arcam Q10plus EBM**	High production rates Good accuracy (layer thickness 50–70 µm) Easy nesting of parts Produces pars with a high surface roughness that improves biological performance Fully dense parts Lower residual stresses than DMLS Easy removal of support structures (manually) No heat treatments required Metals: cobalt chromium alloy (CrCo ASTM F75), Ti-6Al-4V, Ti-6Al-4V ELI, TiCP grade 2, and nickel alloy 718 Compliance with ASTMF136 standards High maintenance time	High maintenance time Only conductive materials Rough surface Needs high quality powder spec Needs high quality powder spec, only supplied by machine brand	[77,80,87,89]
**Company C in-house developed AM system**	Ultra-fast production rate (mass production) Cuts up to 80% in production costs compared to other AM systems Low energy consumption Machine cost is two orders of magnitude lower Complies with metal injection molding standards Metals: Stainless steel Use of standard metal injection molding (MIM) powder material	Only one material is available Parts require heat treatment	

**Table 6:** Quality management systems, control gates, and technologies used by the three studied companies.

Company	Quality System and Certifications	Number of Quality Control Gates	Technologies Used for Quality Control
A	ISO 9001:2015 ISO 13485:2016 ISO 14001:2015 ISO/IEC 27001:2013 Council Directive 93/42/EEC	15	Biomedical software package Finite element analysis software Powder handling and sieving AM equipment Real time AM process monitoring system Micro CNC 5 + 1 axis milling machine Tactile and laser 3D coordinate measuring system Micro-CT scanner Light optical microscope X-Ray fluorescence spectrometer Surface roughness tester
B	ISO 13485:2016 Council Directive 93/42/EEC	14	Biomedical software package Finite element analysis software Powder handling and sieving AM equipment Real time AM process monitoring system Tactile 3D coordinate measuring system Industrial X-ray machine Light optical microscope X-Ray fluorescence spectrometer Surface roughness tester Fatigue testing machine Universal testing machine
C	ISO 9001 ISO 13485 AS9100 MPIF Standard 35	12	Finite element analysis software Powder handling and sieving AM equipment Real time AM process monitoring system Tactile 3D coordinate measuring system Optical measuring system Light optical microscope X-Ray fluorescence spectrometer Surface roughness tester Fatigue testing machine Universal testing machine

**Table 7:** Comparison of Sigma process performance based on their internal production defect rate solely of additive manufactured products.

Company	Production Per Year with AM	Production Defect Rate	Defects Per Million	Six Sigma Rating
A	50	≈4%	≈40,000	≈3.25
B	500	≈0.4%	≈4000	≈4.14
C	24,000	≈40%	≈400,000	≈1.75

**Table 8:** Description and characteristics of the proposed integrated quality control flow diagram.

Quality Control Gate (G)	Inspection Type	Description	Technology and Tools Required
**G-1**: Software validation	Off-line	G-1 is to validate all software used throughout the whole product design workflow and fabrication processes	
**G-2**: Implant specifications	On-line	G-2 is to control communication issues between the surgeon and the clinical engineer. This quality control gate uses an online communication interphase. Through this interphase, the most suitable medical image protocol is decided, and the necessary surgical requirements, patient’s information, and implant specifications are collected and corroborated in a systematic way before proceeding to the next steps of the workflow. Moreover, this interphase allows to perform concurrent surgery planning to identify issues.	Integrated communication interphase
**G-3**: Volumetric reconstruction validation	On-line	In G-3, the 3D volumetric reconstruction is compared to the original medical images from the patient in order to find segmentation mistakes.	Segmentation software, CT images
**G-4**: Computer simulation validation	On-line	In G-4, a 4D implant design approach is used to validate patient-specific implants with patient-specific computational neuromusculoskeletal (NMS) predictions and multiscale finite element analysis (MFEA). Therefore, non-destructive static and dynamic simulations are performed to test the implant design performance. Moreover, a thermo-mechanical simulation is required to identify thermic deformations during the fabrication process. The simulations are carried out two times during the overall design process, one after the primary design process and the other after the final design approval.	Multiscale finite element analysis software package, and biomechanical modeling, simulation and analysis software package
**G-5**: Final design approval	On-line	G-5 takes place as a final design approval. Here, the surgeon is asked to fill out and sign the presurgical planning protocol to approve that the surgical procedure plan, the patient-specific implant design, and its corresponding surgical guides are suitable for the patient. The result of this procedure is a detailed planning report of the preoperative situation, which includes the characteristics of the implant and the expected postoperative situation to be achieved.	Integrated communication interphase
**G-6**: Material supplier validation	On-line	G-6 is used with the purpose of controlling the quality of the powder material that comes from the material supplier. According to each AM equipment supplier, to achieve the highest performance of their specific AM system, it is necessary to use validated powder material, which is strictly supplied by them. However, regardless of who the supplier of the powder material is, the supplier must have a recognized quality management program such as ISO 9001, AS9100, or ISO 13485.	
**G-7**: Blended material validation	On-line	G-7 is performed in order to guarantee the physical and the chemical characteristics of virgin and blended powder. For this purpose, first it is needed to characterize the metal powder to control its characteristics such as particle size distribution, flow rate, particle shape, tap density, oxygen content, and hydrogen content [16]. Moreover, metal powder should have a chemical composition within the established limits required by the ASTM and medical standards and be free from inclusions and impurities.	
**G-8**: AM process validation	Off-line	G-8 is a validation of the AM process that links machine-process and nesting parameters with part mechanical properties and more general dimensional and shape-related metrological parameters. Here, coupons and representative components are also tested using destructive and non-destructive standard methods to verify that dimensional accuracy, mechanical properties, porosity, chemical composition, and material microstructure are within the required quality standards and specifications. This allows one to verify the correct functioning of the AM machine through the identification of links between material properties of coupons and final products, including worst case scenarios and process limitations in relation to machine conditions, part placement, and geometry.	
**G-9**: Real time AM process validation	On-line	Real-time process monitoring is essential for self-regulating process control. Therefore, the objective of G-9 is to monitor, in real time, the most important process parameter of the AM system used. Some of the machine parameters that need to be monitored are: laser or electron beam power and diameter; scanning speed; layer thickness; hatch spacing; bed temperature; melt pool; cooling cycle; chamber temperature, atmosphere, and pressure.	Real-time AM monitoring system
**G-10**: Visual inspection	On-line	G-10 is a visual inspection of the implant surface quality and dimensional deviations. This is required because, during the processes of fabrication, detachment from the build platform, and removal of support structures, dimensional variations and visible surface marks could be introduced.	
**G-11**: Semi-finished product dimensional validation	Off-line	G-11 is a rapid but detailed dimensional validation of the semi-finish components. The dimensional validation of components is performed by an expert that compares each component with the original design and its specified tolerances using basic measurement tools such as caliper and micrometer. However, if the implant’s geometrical complexity does not allow the undertaking of accurate metrological measurements using traditional tools, a more detailed dimensional inspection is required. In this detailed dimensional inspection, a high-resolution point cloud data obtained from a coordinate measuring machine (CMM) and a 3D laser scanner are combined to improve measurement resolution and speed. The result is a deviation map that quantifies critical component sections such as holes for future threads, spherical surfaces, bearing surfaces, and surface roughness. A report is then generated to determine whether the component is rejected or accepted based on the deviation map.	CMM and 3D laser scanner
**G-12**: Periodic metallography and chemical composition inspection	On-line	G-12 is a periodic inspection that takes place to certify that each manufactured batch complies with the required chemical composition and microstructure standards for its specific use. For this purpose, representative test coupons are used. The results of the metallographic examinations should be reported in the device master record with microphotographs of the material microstructure along with a paragraph containing an interpretation of the results. The results of the metallographic examinations should be reported in the device master record with microphotographs of the material microstructure along with a paragraph containing an interpretation of the results.	Light stereo microscope, etching solutions, grinder/polishing machine, microhardness tester, and X-ray fluorescence (XRF) spectrometer
**G-13**: Defectoscopy and dimensional validation	Off-line	G-13’s objective is to perform an evaluation of shape deviations, defectoscopy, and dimensional analysis of semi-finished components in one single test. For this purpose, a micro-CT scanner is used to obtain a 3D representation of the real implant. The dimensional validation is performed with a color deviation map similarly as in G-11. The defectoscopy test looks through the entire part to identify internal pores and powder particles trapped within the trabecular and lattice structures.	Micro-CT scanner
**G-14**: Periodic inspection of mechanical properties	On-line	To guarantee consistent mechanical properties, the objective of the 14th quality control gate is to perform periodic tests of each manufactured batch. For this, the Food and Drug Administration (FDA) recommends the use of test coupons for tensile and micro-hardness tests [26]. The test coupons should be built within each batch, and their location and orientation in the building chamber shall correspond to the worst-case scenarios previously identified in G-8.	Universal testing machine
**G-15**: Surface and coating characterization	On-line	G-15 is a non-destructive quality control gate for implant surface characterization. For modified and non-modified surfaces of metallic implants, there are several surface characteristics at the microscale and the nanoscale that need to be controlled. For this purpose, a noncontact topography characterization is preferred. However, micrometric and nanometric features should be characterized separately.	Non-contact profilometers such as low coherence interferometer, confocal microscope
**G-16**: Detailed periodic random inspection of finished product	Off-line	The objective of G-16 is to perform periodic random destructive tests of standard and bespoke components. In the case of bespoke components, they can only be randomly tested if a strong data base is present. This data base should contain enough information about all the different variations of an implant family to be able to predict the mechanical behavior of its different variations. If this is not the case, bespoke components should be manufactured with a twin coupon to be subjected to the same destructive tests of AM standard components. Moreover, surface properties of coated and non-coated implants also need to be tested. Some of these properties are roughness, hardness, layer thickness, shear fatigue strength, static shear strength, plastic deformation, and abrasion.All of these tests should be performed not just to control quality but also to create a strong data base for continuous improvement of the whole manufacturing process chain. The tests are static and dynamic mechanical tests that should be performed following the corresponding ASTM standards of each component type.	Fatigue testing machine and universal testing machine, indentation hardness tester, scanning electron microscope, and coating thickness gauges
**G-17**: Visual inspection of finished products	On-line	G-17 is a comprehensive visual inspection of the final product. The aim is to detect residual errors that could not be detected in previous stages. Here, an inspector checks the overall quality of each implant and assembly, including all the product documentation from the previous quality control gates. In this quality control gate, the inspector visually compares each component and assembly with the original design and its specified tolerances. Some of the critical areas to be measured are thread holes, assembly tolerances and movement, and height and width of each component.	Caliper, micrometer, magnifying goggles, and schematics
**G-18**: Sterilization and Packaging validation	Off-line	The objective of G-18 is to perform a validation and routine inspections of cleaning, disinfection, sterilization, marking, labeling, and packaging processes. G-18 also includes biocompatibility tests to certify batches. The sterility validation of medical devices at the industrial scale can be performed using a small number of product samples to determine the sterility assurance level (SAL). After validation, the efficiency of disinfection, cleaning, and sterilization processes most be routinely monitored on each cycle. Therefore, during routine production, quality engineers must check sterilization certificates and sterilization indicators.Regarding marking, labeling, and packaging of patient-specific implants, a visual inspection is required. In this visual inspection, it is necessary to verify that each component is adequately marked based on patient information and intended used. Moreover, external package labeling should correspond to it content and follow the corresponding standards. Regarding the main implant package, it is important to inspect it in an exhaustive way to identify potential issues such as punctures, damage, or defective sealing.	Product master record, sterilization certificates, and magnifying goggles

## Supplementary Materials

The following are available online at https://www.mdpi.com/1996-1944/12/19/3110/s1, S1 File: Interviews and questionnaire data collection and analysis details, S2 File: Practitioner companion guide.

## References

[1] Prince J.D. (2014). 3D Printing: An industrial revolution. J. Electron. Resour. Med. Libr..

[2] Bogue R. (2013). 3D printing: The dawn of a new era in manufacturing?. Assem. Autom..

[3] ISO/ASTM (2015). Standard Terminology for Additive Manufacturing-General Principles-Terminology.

[4] Mazzoli A. (2013). Selective laser sintering in biomedical engineering. Med Biol. Eng. Comput..

[5] Ho C.M.B., Ng S.H., Yoon Y.J. (2015). A review on 3D printed bioimplants. Int. J. Precis. Eng. Manuf..

[6] Witowski J., Sitkowski M., Zuzak T., Coles-Black J., Chuen J., Major P., Pdziwiatr M. (2018). From ideas to long-term studies: 3D printing clinical trials review. Int. J. Comput. Assist. Radiol. Surg..

[7] Culmone C., Smit G., Breedveld P. (2019). Additive manufacturing of medical instruments: A state-of-the-art review. Addit. Manuf..

[8] Kulkarni M., Mazare A., Schmuki P., Iglič A., Seifalian A., de Mel A., Kalaskar D.M. (2014). Biomaterial surface modification of titanium and titanium alloys for medical applications. Nanomedicine.

[9] Heness G., Ben-Nissan B. (2004). Innovative bioceramics. Mater. Forum.

[10] Liu Y., Lim J., Teoh S.H. (2013). Review: Development of clinically relevant scaffolds for vascularised bone tissue engineering. Biotechnol. Adv..

[11] Bose S., Roy M., Bandyopadhyay A. (2012). Recent advances in bone tissue engineering scaffolds. Trends Biotechnol..

[12] Katti K.S. (2004). Biomaterials in total joint replacement. Colloids Surf. B Biointerfaces.

[13] Lichte P., Pape H., Pufe T., Kobbe P., Fischer H. (2011). Scaffolds for bone healing: Concepts, materials and evidence. Injury.

[14] Giannoudis P.V., Dinopoulos H., Tsiridis E. (2005). Bone substitutes: An update. Injury.

[15] Lee J.M., Yeong W.Y. (2015). A preliminary model of time-pressure dispensing system for bioprinting based on printing and material parameters: This paper reports a method to predict and control the width of hydrogel filament for bioprinting applications. Virtual Phys. Prototyp..

[16] Hench L.L. (2006). The story of Bioglass^®^. J. Mater. Sci. Mater. Med..

[17] Dhandayuthapani B., Yoshida Y., Maekawa T., Kumar D.S. (2011). Polymeric scaffolds in tissue engineering application: A review. Int. J. Polym. Sci..

[18] Gmeiner R., Deisinger U., Schönherr J., Lechner B., Detsch R., Boccaccini A., Stampfl J. (2015). Additive manufacturing of bioactive glasses and silicate bioceramics. J. Ceram. Sci. Technol.

[19] Anatomics Facial Implants. http://www.anatomics.com/applications/cranio-maxillo-facial/facial-implants/.

[20] Geetha M., Singh A., Asokamani R., Gogia A. (2009). Ti based biomaterials, the ultimate choice for orthopaedic implants—A review. Prog. Mater. Sci..

[21] Liu X., Chu P.K., Ding C. (2004). Surface modification of titanium, titanium alloys, and related materials for biomedical applications. Mater. Sci. Eng. R Rep..

[22] Esmaeilian B., Behdad S., Wang B. (2016). The evolution and future of manufacturing: A review. J. Manuf. Syst..

[23] Zhong R.Y., Xu X., Klotz E., Newman S.T. (2017). Intelligent Manufacturing in the Context of Industry 4.0: A Review. Engineering.

[24] Dennis P. (2007). Lean Production Simplified: A Plain-Language Guide to the World’s Most Powerful Production System.

[25] Wong K.C., Scheinemann P. (2018). Additive manufactured metallic implants for orthopaedic applications. Sci. China Mater..

[26] Parthasarathy J. (2014). 3D modeling, custom implants and its future perspectives in craniofacial surgery. Ann. Maxillofac. Surg..

[27] Ferreira F., Faria J., Azevedo A., Marques A.L. (2017). Product lifecycle management in knowledge intensive collaborative environments: An application to automotive industry. Int. J. Inf. Manag..

[28] Tofail S.A., Koumoulos E.P., Bandyopadhyay A., Bose S., O’Donoghue L., Charitidis C. (2018). Additive manufacturing: Scientific and technological challenges, market uptake and opportunities. Mater. Today.

[29] Hollister S.J., Crotts S.J., Ramaraju H., Flanagan C.L., Zopf D.A., Morrison R.J., Les A., Ohye R.G., Green G.E. (2018). Quality Control of 3D Printed Resorbable Implants: The 3D Printed Airway Splint Example. 3D Print. Biofabrication.

[30] Allen R.H., Sriram R.D. (2000). The role of standards in innovation. Technol. Forecast. Soc. Chang..

[31] Shin D.-H., Kim H., Hwang J. (2015). Standardization revisited: A critical literature review on standards and innovation. Comput. Stand. Interfaces.

[32] ISO-13485 (2016). Medical Devices—Quality Management Systems—Requirements for Regulatory Purposes.

[33] Brown A., Eatock J., Dixon D., Meenan B.J., Anderson J. (2008). Quality and continuous improvement in medical device manufacturing. TQM J..

[34] Sangshetti J.N., Deshpande M., Zaheer Z., Shinde D.B., Arote R. (2014). Quality by design approach: Regulatory need. Arab. J. Chem..

[35] Hollister S.J., Flanagan C.L., Zopf D.A., Morrison R.J., Nasser H., Patel J.J., Ebramzadeh E., Sangiorgio S.N., Wheeler M.B., Green G.E. (2015). Design control for clinical translation of 3D printed modular scaffolds. Ann. Biomed. Eng..

[36] FDA, U.S. Department of Health and Human Services (2017). Technical Considerations for Additive Manufactured Medical Devices: Guidance for Industry and Food and Drug Administration Staff.

[37] Clemens N. (2017). The New European Medical Device Regulation 2017/745: Main Changes and Challenges.

[38] Union E. (2017). Regulation (EU) 2017/745 of the European Parliament and of the Council of 5 April 2017 on medical devices, amending Directive 2001/83/EC, Regulation (EC) No 178/2002 and Regulation (EC) No 1223/2009 and repealing Council Directives 90/385/EEC and 93/42/EEC. Off. J. Eur. Union.

[39] TGA, Department of Health Therapeutic Goods Administration (2017). Proposed Regulatory Changes Related to Personalised and 3D Printed Medical Devices Consultation Paper.

[40] ASTM What Is ASTM?. https://www.astm.org/ABOUT/factsheet.html.

[41] ISO About ISO. https://www.iso.org/about-us.html.

[42] Frits F., Klas B., Benoit V., Adriaan S., Henk B., Martin S., Suny M., Dominique T., Mario M., Commision E. (2014). Support Action for Standardisation in Additive Manufacturing (SASAM).

[43] Ituarte I.F., Coatanea E., Salmi M., Tuomi J., Partanen J. (2015). Additive manufacturing in production: A study case applying technical requirements. Phys. Procedia.

[44] Everton S.K., Hirsch M., Stravroulakis P., Leach R.K., Clare A.T. (2016). Review of in-situ process monitoring and in-situ metrology for metal additive manufacturing. Mater. Des..

[45] Martinez-Marquez D., Mirnajafizadeh A., Carty C.P., Stewart R.A. (2019). Facilitating industry translation of custom 3d printed bone prostheses and scaffolds through Quality by Design. Procedia Manuf..

[46] Gausemeier J., Echterhoff N., Kokoschka M., Wall M. (2011). Thinking ahead the Future of Additive Manufacturing–Future Applications.

[47] Geremia F. (2018). Quality aspects for medical devices, quality system and certification process. Microchem. J..

[48] McCutcheon D.M., Meredith J.R. (1993). Conducting case study research in operations management. J. Oper. Manag..

[49] Tharenou P., Donohue R., Cooper B. (2007). Management Research Methods.

[50] Leedy P.D., Ormrod J.E. (2013). Practical Research: Planning and Design.

[51] Tong A., Sainsbury P., Craig J. (2007). Consolidated criteria for reporting qualitative research (COREQ): A 32-item checklist for interviews and focus groups. Int. J. Qual. Health Care.

[52] Oyegoke A. (2011). The constructive research approach in project management research. Int. J. Manag. Proj. Bus..

[53] Maylor H., Blackmon K.L. (2005). Researching Business and Management.

[54] Harrell M.C., Bradley M.A. (2009). Data Collection Methods. Semi-Structured Interviews and Focus Groups.

[55] Hussey J., Hussey R. (1997). Business Research: A Practical Guide for Undergraduate and Postgraduate Students.

[56] University of Wisconsin Data Collection Methods. https://people.uwec.edu/piercech/ResearchMethods/Data%20collection%20methods/DATA%20COLLECTION%20METHODS.htm.

[57] Anderson C. (2010). Presenting and evaluating qualitative research. Am. J. Pharm. Educ..

[58] DiCicco-Bloom B., Crabtree B.F. (2006). The qualitative research interview. Med Educ..

[59] Eisenhardt K.M. (1989). Building Theories from Case Study Research. Acad. Manag. Rev..

[60] Qian B., Shen Z. (2013). Laser sintering of ceramics. J. Asian Ceram. Soc..

[61] EOS EOSINT M 280. https://platforms.monash.edu/mcam/images/stories/Eos/eosint_m280_e.pdf.

[62] EOS EOS P 936. https://www.eos.info/systems_solutions/plastic/systems_equipment/eos_p_396.

[63] Butler J. (2011). Using selective laser sintering for manufacturing. Assem. Autom..

[64] Bertol L.S., Júnior W.K., da Silva F.P., Aumund-Kopp C. (2010). Medical design: Direct metal laser sintering of Ti-6Al-4V. Mater. Des..

[65] Becker T.H., Beck M., Scheffer C. (2015). Microstructure and mechanical properties of direct metal laser sintered Ti-6Al-4V. South Afr. J. Ind. Eng..

[66] Qian M., Xu W., Brandt M., Tang H. (2016). Additive manufacturing and postprocessing of Ti-6Al-4V for superior mechanical properties. MRS Bull..

[67] Petrovic V., Vicente Haro Gonzalez J., Jorda Ferrando O., Delgado Gordillo J., Ramon Blasco Puchades J., Portoles Grinan L. (2011). Additive layered manufacturing: Sectors of industrial application shown through case studies. Int. J. Prod. Res..

[68] Ligon S.C., Liska R., Stampfl J., Gurr M., Mülhaupt R. (2017). Polymers for 3D printing and customized additive manufacturing. Chem. Rev..

[69] Shirazi S.F.S., Gharehkhani S., Mehrali M., Yarmand H., Metselaar H.S.C., Kadri N.A., Osman N.A.A. (2015). A review on powder-based additive manufacturing for tissue engineering: Selective laser sintering and inkjet 3D printing. Sci. Technol. Adv. Mater..

[70] Stansbury J.W., Idacavage M.J. (2016). 3D printing with polymers: Challenges among expanding options and opportunities. Dent. Mater..

[71] Levy G.N., Schindel R., Kruth J.P. (2003). Rapid manufacturing and rapid tooling with layer manufacturing (LM) technologies, state of the art and future perspectives. CIRP Ann. Manuf. Technol..

[72] Barui S., Mandal S., Basu B. (2017). Thermal inkjet 3D powder printing of metals and alloys: Current status and challenges. Curr. Opin. Biomed. Eng..

[73] O’Brien E.K., Wayne D.B., Barsness K.A., McGaghie W.C., Barsuk J.H. (2016). Use of 3D printing for medical education models in transplantation medicine: A critical review. Curr. Transplant. Rep..

[74] Peng Q., Tang Z., Liu O., Peng Z. (2015). Rapid prototyping-assisted maxillofacial reconstruction. Ann. Med..

[75] Park S.W., Choi J.W., Koh K.S., Oh T.S. (2015). Mirror-imaged rapid prototype skull model and pre-molded synthetic scaffold to achieve optimal orbital cavity reconstruction. J. Oral Maxillofac. Surg..

[76] Butscher A., Bohner M., Hofmann S., Gauckler L., Müller R. (2011). Structural and material approaches to bone tissue engineering in powder-based three-dimensional printing. Acta Biomater..

[77] Gao W., Zhang Y., Ramanujan D., Ramani K., Chen Y., Williams C.B., Wang C.C., Shin Y.C., Zhang S., Zavattieri P.D. (2015). The status, challenges, and future of additive manufacturing in engineering. Comput. Aided Des..

[78] Harrysson O.L., Cansizoglu O., Marcellin-Little D.J., Cormier D.R., West H.A. (2008). Direct metal fabrication of titanium implants with tailored materials and mechanical properties using electron beam melting technology. Mater. Sci. Eng. C.

[79] Gong H., Rafi K., Gu H., Starr T., Stucker B. (2014). Analysis of defect generation in Ti-6Al-4V parts made using powder bed fusion additive manufacturing processes. Addit. Manuf..

[80] Kircher R., Christensen A., Wurth K. (2009). Electron beam melted (EBM) Co-Cr-Mo alloy for orthopaedic implant applications. Austin Solid Free Fabr..

[81] Sing S.L., An J., Yeong W.Y., Wiria F.E. (2016). Laser and electron-beam powder-bed additive manufacturing of metallic implants: A review on processes, materials and designs. J. Orthop. Res..

[82] Frazier W.E. (2014). Metal additive manufacturing: A review. J. Mater. Eng. Perform..

[83] Lampin M., Warocquier C., Legris C., Degrange M., Sigot-Luizard M.F. (1997). Correlation between substratum roughness and wettability, cell adhesion, and cell migration. J. Biomed. Mater. Res..

[84] Barrere F., Mahmood T., De Groot K., Van Blitterswijk C. (2008). Advanced biomaterials for skeletal tissue regeneration: Instructive and smart functions. Mater. Sci. Eng. R Rep..

[85] Nicoletto G., Konečná R., Frkáň M., Riva E. (2018). Surface roughness and directional fatigue behavior of as-built EBM and DMLS Ti-6Al-4V. Int. J. Fatigue.

[86] Arcam A.B. Metal powders. http://www.arcam.com/technology/products/metal-powders/.

[87] Bartlett J.L., Li X. (2019). An overview of residual stresses in metal powder bed fusion. Addit. Manuf..

[88] Khorasani A., Gibson I., Awan U.S., Ghaderi A. (2019). The effect of SLM process parameters on density, hardness, tensile strength and surface quality of Ti-6Al-4V. Addit. Manuf..

[89] Baufeld B., Van der Biest O., Gault R. (2010). Additive manufacturing of Ti-6Al-4V components by shaped metal deposition: Microstructure and mechanical properties. Mater. Des..

[90] Jabnoun N. (2002). Control processes for total quality management and quality assurance. Work Study.

[91] Psomas E., Antony J. (2015). The effectiveness of the ISO 9001 quality management system and its influential critical factors in Greek manufacturing companies. Int. J. Prod. Res..

[92] Ramachandran S.D., Chong S.C., Ismail H. (2011). Organisational culture: An exploratory study comparing faculties’ perspectives within public and private universities in Malaysia. Int. J. Educ. Manag..

[93] Mohamed S.S., YuanJian Q. The impact of the organizational culture on the implementation of TQM programs. Proceedings of the 2008ISECS International Colloquium on Computing, Communication, Control, and Management, CCCM’08.

[94] Stanberry B. (2006). Legal and ethical aspects of telemedicine. J. Telemed. Telecare.

[95] Ozusaglam S., Robin S., Wong C.Y. (2018). Early and late adopters of ISO 14001-type standards: Revisiting the role of firm characteristics and capabilities. J. Technol. Transf..

[96] ISO/IEC (2013). Information Technology-Security Techniques-Information Security Management Systems-Requirements.

[97] Tomić B., Spasojević-Brkić V., Klarin M. (2012). Quality management system for the aerospace industry. J. Eng. Manag. Compet..

[98] Sissell K. (1996). Survey Rates ISO 9000 Success.

[99] Aba E.K., Badar M.A., Hayden M.A. (2016). Impact of ISO 9001 certification on firms financial operating performance. Int. J. Qual. Reliab. Manag..

[100] Antony J., Kumar M., Labib A. (2008). Gearing Six Sigma into UK manufacturing SMEs: Results from a pilot study. J. Oper. Res. Soc..

[101] Antony J., Kumar M., Madu C.N. (2005). Six sigma in small- and medium-sized UK manufacturing enterprises: Some empirical observations. Int. J. Qual. Reliab. Manag..

[102] Chiarini A. (2015). Effect of ISO 9001 non-conformity process on cost of poor quality in capital-intensive sectors. Int. J. Qual. Reliab. Manag..

[103] Zhu Z. (1999). A comparison of quality programmes: Total quality management and ISO 9000. Total Qual. Manag..

[104] Chen C.K., Anchecta K., Lee Y.D., Dahlgaard J.J. (2016). A stepwise ISO-based TQM implementation approach using ISO 9001: 2015. Manag. Prod. Eng. Rev..

[105] Fonseca L.M. (2015). From Quality Gurus and TQM to ISO 9001: 2015: A review of several quality paths. Int. J. Qual. Res..

[106] Marques P.A., Meyrelles P.M., Saraiva P.M., Frazao-Guerreiro F.J. Integrating Lean Six Sigma with ISO 9001:2015. Proceedings of the 2016 IEEE International Conference on Industrial Engineering and Engineering Management.

[107] Dasgupta T. (2003). Using the six-sigma metric to measure and improve the performance of a supply chain. Total Qual. Manag. Bus. Excell..

[108] Taghizadegan S. (2006). Essentials of Lean Six Sigma.

[109] Koch P.N., Yang R.J., Gu L.J.S., Optimization M. (2004). Design for six sigma through robust optimization. Struct. Multidiscip. Optim..

[110] Senvar O., Tozan H. (2010). Process capability and six sigma methodology including fuzzy and lean approaches. Products and Services; from R&D to Final Solutions.

[111] Shahin A. (2008). Design for Six Sigma (DFSS): Lessons learned from world-class companies. Int. J. Six Sigma Compet. Advant..

[112] Henderson K.M., Evans J.R. (2000). Successful implementation of Six Sigma: Benchmarking General Electric Company. Benchmarking Int. J..

[113] Antony J., Desai D.A. (2009). Assessing the status of Six Sigma implementation in the Indian industry: Results from an exploratory empirical study. Manag. Res. News.

[114] Hrgarek N., Bowers K.A. (2009). Integrating six sigma into a quality management system in the medical device industry. J. Inf. Organ. Sci..

[115] Crago M.G. (2000). Patient safety, six sigma & ISO 9000 quality management. Qual. Dig..

[116] Shane S., Stuart T. (2002). Organizational endowments and the performance of university start-ups. Manag. Sci..

[117] Shi J., Zhou S. (2009). Quality control and improvement for multistage systems: A survey. IIE Trans..

[118] Colledani M., Tolio T., Fischer A., Iung B., Lanza G., Schmitt R., Váncza J. (2014). Design and management of manufacturing systems for production quality. CIRP Ann. Manuf. Technol..

[119] Chang D. (2013). Internalizing the External Costs of Medical Device Preemption. Hastings LJ.

[120] He D. (2010). Engineering Quality Systems: Cost of Quality. Mod. Appl. Sci..

[121] Bettayeb B., Bassetto S.J., Sahnoun M. (2014). Quality control planning to prevent excessive scrap production. J. Manuf. Syst..

[122] Nicholas J.M., Steyn H. (2008). Project Management for Business, Engineering, and Technology: Principles and Practice.

[123] Martinez-Marquez D., Mirnajafizadeh A., Carty C.P., Stewart R.A. (2018). Application of quality by design for 3D printed bone prostheses and scaffolds. PLoS ONE.

[124] Appleton E. (2008). Product Design for Manufacture and Assembly. Assem. Autom..

[125] Craft R.C., Leake C. (2002). The Pareto principle in organizational decision making. Manag. Decis..

[126] Junior O.C., Okumura M.L.M., Young R.I.M. (2015). The application of an integrated product development process to the design of medical equipment. Concurrent Engineering in the 21st Century.

[127] Kujawińska A., Vogt K. (2015). Human factors in visual quality control. Manag. Prod. Eng. Rev..

[128] Bhat K.S., Ebrary I. (2010). Total Quality Management: Text and Cases.

[129] Terlaak A., King A.A. (2006). The effect of certification with the ISO 9000 Quality Management Standard: A signaling approach. J. Econ. Behav. Organ..

[130] Smith C.G., Cooper A.C. (1988). Established companies diversifying into young industries: A comparison of firms with different levels of performance. Strateg. Manag. J..

[131] Friesl M. (2012). Knowledge acquisition strategies and company performance in young high technology companies. Br. J. Manag..

[132] Vasconcellos e Sá J. (1988). The impact of key success factors on company performance. Long Range Plan..

[133] Brown S., Blackmon K. (2005). Aligning manufacturing strategy and business-level competitive strategy in new competitive environments: The case for strategic resonance. J. Manag. Stud..

[134] Makhija M. (2003). Comparing the Resource-Based and Market-Based Views of the Firm: Empirical Evidence from Czech Privatization. Strateg. Manag. J..

[135] Zahra S.A. (1996). Technology strategy and new venture performance: A study of corporate-sponsored and independent biotechnology ventures. J. Bus. Ventur..

[136] Zahra S.A., George G. (1999). Manufacturing strategy and new venture performance: A comparison of independent and corporate ventures in the biotechnology industry. J. High Technol. Manag. Res..

[137] Shetwan A.G., Vitanov V.I., Tjahjono B. (2011). Allocation of quality control stations in multistage manufacturing systems. Comput. Ind. Eng..

[138] Groll J., Boland T., Blunk T., Burdick J.A., Cho D.-W., Dalton P.D., Derby B., Forgacs G., Li Q., Mironov V.A. (2016). Biofabrication: Reappraising the definition of an evolving field. Biofabrication.

[139] Lee J.M., Ng W.L., Yeong W.Y. (2019). Resolution and shape in bioprinting: Strategizing towards complex tissue and organ printing. Appl. Phys. Rev..

[140] TGA, Department of Health Therapeutic Goods Administration (2017). Proposed Regulatory Changes Related to Personalised and 3D Printed Medical Devices.

[141] Lee J.M., Sing S.L., Zhou M., Yeong W.Y. (2018). 3D bioprinting processes: A perspective on classification and terminology. Int. J. Bioprint..

[142] Moroni L., Boland T., Burdick J.A., De Maria C., Derby B., Forgacs G., Groll J., Li Q., Malda J., Mironov V.A. (2018). Biofabrication: A guide to technology and terminology. Trends Biotechnol..

[143] Ng W.L., Wang S., Yeong W.Y., Naing M.W. (2016). Skin bioprinting: Impending reality or fantasy?. Trends Biotechnol..

