# Supplementary material for: Development of 18 Quality Control Gates for Additive Manufacturing of Error Free Patient-Specific Implants
Source: Materials (Basel). 2019 Sep 24;12(19):3110. doi: 10.3390/ma12193110 (PMC6803939; doi:10.3390/ma12193110)
Supplement: Supplementary file 1 [file materials-12-03110-s001.zip › S1_FILE-1.pdf]

materials

MDPI

Supplementary Material S1

Development of 18 quality control gates for additive manufacturing of error free patient-specific implants

Daniel Martinez-Marquez [a], Milda Jokymaityte [b], Ali Mirnajafizadeh [c], Christopher P. Carty [d,e], David Lloyd [d], and Rodney A. Stewart [a*]

[a]*School of Engineering, Griffith University, Gold Coast, QLD, Australia*
[b]*Ortho Baltic, Kaunas, Lithuania*
[c]*Molecular Cell Biomechanics Laboratory, University of California, Berkeley, CA, USA*
[d]*School of Allied Health Sciences and Gold Coast Orthopaedic Research and Education Alliance, Menzies Health Institute Queensland, Griffith University, Gold Coast, QLD, Australia*
[e]*Department of Orthopaedic Surgery, Queensland Children's Hospital, Children's Health Queensland Hospital and Health Service, Brisbane, QLD, Australia*

* Corresponding author. Tel.: +61 (7) 5552 8778
E-mail address: r.stewart@griffith.edu.au

Note: Reference this document same as the main article

Contents

Data Collection.......... 1
Study selection.......... 2
Data extraction and analysis.......... 2
Questionnaire Guide.......... 4
    Interview questions and steps.......... 4
Summary of comparative analysis of companies' quality control gates and methods.......... 5
Assistance interview documents.......... 10

Data Collection

Conducting case study in business and management research requires the gathering of primary data through interviews and questionnaires from key individuals such as managers, workers, and technical staff to extract expert knowledge about their experiences, beliefs, or opinions [1-3]. Interviews can be classifies in structure, unstructured, and semi-structured interviews. Structured interviews are closely associated with the scientific approach, where data collection is inflexible, giving no room to changes. Structured interviews usually collect quantitative data through a set of previously prepared closed questions [4]. This represent the advantage of structured interviews, where the data is collected in a consistent way across interviews, minimizing the differences between each interview to increase its reliability [2]. Unstructured interviews are compose by unstructured questions that have not been prepared beforehand [4]. This kind of interviews have the disadvantages of been difficult to control, are very time consuming, and may lead to problems recording the questions and answers [4]. The great flexibility of unstructured interviews lead to collect only qualitative data laminating its applicability. On the other hand, semi-structured interviews have the advantages of both structure and unstructured interviews providing a balance between flexibility and rigidity. Semi-structured interviews are

conversational interviews composed of a set of standardized open-ended questions, with new questions that emerge through the interview process [5]. This freedom through the interview process, allow to reveal new and different aspects of the topic, giving strength to this kind of interviews [4]. Semi-structured interviews are mainly used to gather qualitative data, and when the researcher wants to delve deeply into a topic and to understand thoroughly the answers provided [4]. Nevertheless, there are occasions where qualitative research tools, such as semi-structured interviews can provide quantitative data [6]. Moreover, face-to-face interviews have the advantage of having the highest response rate in survey research [7]. Additionally, face-to-face interviews capture the most detail of both verbal and nonverbal communication, and provide a space to establish rapport with participants, permitting the researcher to clarify ambiguous answers during the interview [7]. Therefore, the team selected face-to face interviews as the main data collection method for this research. The interviews were aimed at achieving four main objectives: (1) Identifying the workflow process of the design and fabrication processes of additively manufactured implants; (2) Identifying quality issues and percentages of rework and scrap; (3) Identifying technologies and methodologies used for quality control and assurance; and (4) Identifying communication and interaction methods between companies and clients (surgeons).

Study selection

The criteria to select the companies for this study were based on their experience and expertise in the design and manufacturing of medical devices using AM technologies. These include companies in the aerospace field due to their shared similarities in relation to materials used and strict quality regulations. Hence, the companies selected for this study had to comply with at least one of the following criteria: (1) Companies that manufacture patient-specific implants and/or medical devices using AM; (2) Companies that design patient-specific implants and/or medical devices for AM; (3) Companies that manufacture aerospace parts with AM technologies; and (4) Companies that design aerospace parts for AM.

Data extraction and analysis

For the data extraction of this study the team developed a semi-structured guide and a PowerPoint presentation to be conducted in the form of face-to-face interview in the premises of each company following the consolidated criteria for reporting qualitative research (COREQ) [8]. A research information sheet and a consent form were developed and delivered at the beginning of each interview. The purpose of the information sheet was to provide a detailed description of this study and the type of information that would be requested from each company. The consent form described that the identity of each participant will be considered confidential and that only a de-identified summary of results may be used for presentations and publications. Moreover, a protocol composed of 11 steps was developed to perform the semi-structured interviews in the following manner: (1) An invitation letter will be send via email explaining the purpose of the research and why is important the participation of experts; (2) After each participant confirm his/her participation, meeting time and place will be arranged for conducting the interview taking into consideration to choose the locations to be convenient for each participant; (3) The interviewees identity will be kept confidential and code numbers will be assign to each of them; (4) Each interviewee will receive at the beginning of the interview a consent form where they agree to be audio recorded, and that the information that they will provide will be considered confidential and the summary of results of this study may be used for presentations and publications; (5) The interviews were designed to last minimum 90 minutes; (6) At the start of the interview, the researcher will introduce himself, and explain the nature of the interview; (7) Then, PowerPoint presentation with the results of our previous studies and other preliminary results will be performed, followed by a discussion; (8) Next, each participant will be asked to talk about his/her experience

related to fabrication and design of patient-specific implants with additive manufacturing; (9) The questionnaire then will be performed; (10) The interviews will have a process of continuous refinement. Therefore, after each interview, a preliminary analysis will be carried out with the purpose of identifying other questions to be used in the following interviews and which ones could be omitted; (11) Additionally, each interviewee will be asked to suggest other industry experts from his/her network to be interviewed.

The interview guide was composed by constructing a set of 28 open-ended questions to guide the direction of the conversation. The 28 questions were distributed in 6 different sections of the questionnaire for the purpose of:

1. Acquire the background of the expert interviewed and the company.
2. Identify the workflow process of the design and fabrication processes of additively manufactured patient-specific implants.
3. Identify how is the communication and interaction between the company and client (Surgeon)
4. Identify quality issues and percentage of rework and scrap (Industry perspective), including preventive and reactive solutions to these issues.
5. Obtain historic documentation of production and quality issues
6. Identify technologies and methodologies used for quality assurance.

Questionnaires have several advantages in comparison to other survey methods. For example, they are easy to design and the overall time to develop and administer is minimal. Moreover, large amount of data with a high level of reliability can be collected at low cost, make it the least-costly survey instrument [9]. However, when questionnaires are composed of a large set of questions there is always a high probability that the interviewees do not complete the entire questionnaire [10]. Therefore, short questionnaires are more effective and realisable because are easier to complete [10]. The type of questions asked during each interview where descriptive and structural. Descriptive questions are used to gather descriptions of things and processes in order to get insights, or to check validity or accuracy about them [3]. In contrast, structural questions help the researcher to categorize groups of things and processes and to understand its relationships [3]. An example of the 28 questions used in this study can be seen at the end of this section.

In regards to the data analysis, this was performed following within-case and cross-case analysis approaches [11]. In this study the within-case analysis was concerned with the evaluation of the collected data, as well as the reporting of the findings of each individual case study. The information obtained from each interview and visits to the manufacturing premises, provided a clear understanding of the design, fabrication, and quality control process of each company. Following this, the cross-case analysis was performed between the technologies and processes of the studied companies, with the purpose of making a comparative analysis of their advantages and disadvantages [1], in order to later produce an integrated quality control flow diagram that contains the best practices of each company.

materials

MDPI

Questionnaire Guide

Interview questions and steps

1. **<u>Background information Questions</u>**
   Researcher/Industry expert:

- Research field or field of expertise?
- Current position and responsibilities?
- Field of education/ and research?
- Years of experience?

Company:

- What type of products your company produce?
- How many years your company have been in the market?
- How many employees does your company have?

2. **<u>Identify the workflow process of the design and fabrication processes of 3D printed bone implants</u>**

- What are the main activities and processes necessary to design and fabricate a product?
- What are the key technologies and software used to perform these activities and processes?
- From these activities and processes which ones are the most critical in relation to the quality of the final product?

3. **<u>Identify how is the communication and interaction between the company and client (Surgeon)</u>**

- How the company and the client interact to design the product?
- In what processes and activities is the client involved?
- Which are the most common barriers and issues that can emerge during the client interaction process?
- How these issues and barriers are usually overcome?

4. **<u>Identify quality issues and percentage of rework and scrap.</u>** Precision, accuracy, consistency taste, specification (**PACTS**)

- In average how many implants are produced per month/year?
- From this production rate, in average how many products have to be discharged due to quality issues? Or what is the percentage of scrap?
- What activities or processes are the ones that required more rework or extra hours? In other words, what activities are that ones that most often have to be repeated due to mistakes?
- What are the most common issues that can emerge during the design and fabrication activities necessary to achieve the final product?
- From these issues, which ones represent a potential threat to the quality of the final product?

5. **Required documentation**
    - Routinely product quality tests
    - Production historical data
    - Historical data of production issues and defective products: rework and scrap

6. **Identify technologies and methodologies used for quality assurance.**

- What Quality Assurance system do you use? E.g. Total Quality management, Lean six sigma, etc.
- What are the strategies used to prevent or mitigate these potential issues?
- What are the key technologies and activities used to monitor and guarantee the quality of products?
- What are the variables that you use to measure product quality?
- What are the acceptable tolerances for these variables to satisfy product quality?
- What are the geometrical tolerances in your products? Or the precision required? E.g how many millimetres or micrometres are your product allowed to be offset in relation to patient's specific geometry.

Summary of cross analysis of companies' quality control gates and methods

Quality control gate (G)	Inspection Type	Description	Technology and tools required	Company A	Company B	Company c
**G-1: Software validation**	Off-line	G-1 is to validate all software used throughout the whole product design workflow and fabrication processes		✓	✓	✓
				Complies with FDA regulations	Complies with FDA regulations	Complies with FDA regulations
**G-2: Implant specifications**	On-line	Control communication issues between the surgeon and the clinical engineer. Through this interphase the most suitable medical image protocol is decided, and the necessary surgical requirements, patient's information, and implant specifications are collected and corroborated in a systematic way before proceeding to the next steps of the workflow.	Integrated communication interphase	In-house developed Integrated communication interphase.<br>Concurrent surgery planning to identify issues.	Integrated communication interphase	✗
				Reduces the risks of poor design.<br>Maximises stakeholders' involvement.	Reduces the risks of poor design.<br>Facilitates stakeholders' involvement.	
**G-3: Volumetric reconstruction validation**	On-line	In G-3 the 3D volumetric reconstruction is compared to the original medical images from the patient, in order to find segmentation mistakes.	Segmentation software, CT images	✓	✓	✓
				This process is compulsory for any patient-specific implant	This process is compulsory for any patient-specific implant	This process is compulsory for any patient-specific implant
**G-4: Computer simulation validation**	On-line	Non-destructive static and dynamic simulations are performed to test the implant design performance. Moreover, a thermo-mechanical simulation is required to identify thermic deformations during the fabrication process. The simulations are carried out two times during the overall design process, one after the primary design process and the other after the final design approval.		Finite element analysis software package.<br>Collaboration with university research groups for computational	Finite element analysis software package.<br>Used of published scientific articles with results of computational	Multiscale finite element analysis software package

				neuromusculoskeletal (NMS) predictions.<br><br>Biomechanical modelling, simulation and analysis software package	neuromusculoskeletal (NMS) predictions.	
				The results of simulations may be too far from reality<br><br>Patient-specific neuromusculoskeletal models provide better approximations.	The results of simulations may be too far from reality.	The use of multiscale simulations allow to better understand implant material behaviour. However this is only possible if real forces obtained from Patient-specific neuromusculoskeletal models are used.
**G-5: Final design approval**	On-line	Here the surgeon is asked to fill out and sign the presurgical planning protocol to approve that the surgical procedure plan, the patient-specific implant design and its corresponding surgical guides are suitable for the patient. The result of this procedure is a detailed planning report of the preoperative situation, which includes the characteristics of the implant and the expected postoperative situation to be achieved.	Integrated communication interphase	✓<br>Integrated communication interphase	✓<br>Integrated communication interphase	✓
				Allow to better control client requirements.<br><br>Reduces risks and complains from client.	Allow to better control client requirements.<br><br>Reduces risks and complains from client.	Allow to better control client requirements.<br><br>Reduces risks and complains from client
**G-6: Material supplier validation**	On-line	Control the quality of the powder material that comes from the material supplier. According to each AM equipment supplier, to achieve the highest performance of their specific AM system it is necessary to use validated powder material, which is strictly supplied by them. However, regardless who is the supplier of the powder material, the supplier must have a recognized quality management program such as ISO 9001, AS9100, or ISO 13485		✓<br>Powder suppliers are the same suppliers of the AM machine	✓<br>Powder suppliers are the same suppliers of the AM machine	✓<br>Used of standard powder for metal injection
				Due to low medical standards for AM industry, there is a high risk with regulation changes.	Due to low medical standards for AM industry, there is a high risk with regulation changes.	Facilitates standardization and material quality control.
**G-7: Blended material validation**	On-line	G-7 is performed in order to guarantee the physical and chemical characteristics of virgin and blended powder. For this purpose, first it is needed to characterize the metal powder to control its characteristics such as particle size distribution, flow rate, particle shape, tap density, oxygen content, and hydrogen content [16]. Moreover, metal powder should have a chemical composition within the established limits required by the ASTM and medical standards and be free from inclusions and impurities.		✓	✓	✓
				This is a more strict validation process	This is a more strict validation process	Easy validation process
**G-8: AM process validation**	Off-line	Validation of the AM process that links machine-process and nesting parameters with part mechanical properties, and more general dimensional and shape-related metrological parameters. Here coupons and representative components are also tested using destructive and non-destructive standard methods to verify that the dimensional accuracy, mechanical properties, porosity, chemical composition, and material microstructure are within the required quality standards and specifications. This allows to verify the correct functioning of the AM machine through the identification of links between material properties		✓	✓	✓

		of coupons and final products, including worst case scenarios and process limitations in relation to machine conditions, part placement and geometry.		This is required in order to acquired FDA approval	This is required in order to acquired FDA approval	This is required in order to acquired FDA approval
**G-9: Real time AM process validation**	On-line	Real-time process monitoring is essential for self-regulating process control. Therefore, the objective of G-9 is to monitor in real time the most important process parameter of the AM system used. Some of the machine parameters that need to be monitored are: laser or electron beam power, and diameter; scanning speed; layer thickness; hatch spacing; bed temperature; melt pool; cooling cycle; chamber temperature, atmosphere, and pressure.	Real-time AM monitoring system	✓	✓	✗
				Facilitates quality control	Facilitates quality control	More quality control activities are require.<br>More expertise in AM process is required
**G-10: Visual inspection**	On-line	Visual inspection of the implant surface quality and dimensional deviations. This is required because during the processes of fabrication, detachment from the build platform, and removal of support structures dimensional variations and visible surface marks could be introduced.		✓	✓	✓
				No comments	No comments	No comments
**G-11: Semi-finished product dimensional validation**	Off-line	Dimensional validation of the semi-finish components. The dimensional validation of components is performed by an expert that compares each component with the original design and its specified tolerances using basic measurement tools such as caliper and micrometer. However, if the implants geometrical complexity does not allow the undertaking of accurate metrological measurements using traditional tools a more detailed dimensional inspection is required. In this detailed dimensional inspection, a high-resolution point cloud data obtained from a CMM and a 3D laser scanner are combined to improve measurement resolution and speed. The result is a deviation map that quantifies critical component sections such as holes for future threads, spherical surfaces, bearing surfaces, and surface roughness. A report is then generated to determine whether the component is rejected or accepted based on the deviation map.		✓	✓	✓
				Use of simple tools such as caliper and micrometer.<br>CMM and 3D laser scanner<br>These tools allow a more a precise measurement, thus minimising cost of moving the product forward through the workflow process. However they make this process much slower.	Use of simple tools such as caliper and micrometer.<br>CMM<br>At this stage this tools provide a fast and cost-effective measurement method	Use of simple tools such as caliper and micrometer.<br>CMM<br>At this stage this tools provide a fast and cost-effective measurement method

Table 8. (Continued)

Quality control gate (G)	Inspection Type	Description	Technology and tools required	Company A	Company B	Company c
**G-12: Periodic metallography and chemical composition inspection**	On-line	Periodic inspection that takes place to certify that each manufactured batch complies with the required chemical composition and microstructure standards for its specific use. For this purpose, representative test coupons are used. The results of the metallographic examinations should be reported in the device master record with microphotographs of the material microstructure along with a paragraph containing an interpretation of the results. The results of the metallographic examinations should be reported in the device master record with microphotographs of the material microstructure along with a paragraph containing an interpretation of the results.	Light stereo microscope, etching solutions, grinder/polishing machine, Microhardness tester, and XRF spectrometer	✓<br>This is required in order to acquired FDA approval	✓<br>This is required in order to acquired FDA approval	✓<br>This is required in order to acquired FDA approval
**G-13: Defectoscopy and dimensional validation**	Off-line	Evaluation of shape deviations, defectoscopy and dimensional analysis of semifinished components in one single test. The defectoscopy test looks through the entire part to check to identify internal pores, and powder particles trapped within the trabecular and lattice structures.	Micro-CT scanner	Micro-CT scanner<br>This tool is the most appropriate for this task, and has been intensively used by NASA and aerospace companies.	X-ray machine<br>This tool are not the best option to perform detailed dimensional measurements for patient-specify implants. They are slow and don't allow a complete geometry measurement	X-ray machine<br>This tool are not the best option to perform detailed dimensional measurements for patient-specify implants. They are slow and don't allow a complete geometry measurement
**G-14: Periodic inspection of mechanical properties**	On-line	Periodic tests of each manufactured batch. For this the FDA recommends the use of test coupons for tensile and micro-hardness tests. The test coupons should be built within each batch, and their location and orientation in the building chamber shall correspond to the worst-case scenarios previously identified in G-8.	Universal testing machine	✗<br>The omission of this process can raise many potential critical risks.	✓<br>Due to the high uncreatability in AM this practice is recommended	✓<br>Due to the high uncreatability in AM this practice is recommended
**G-15: Surface and coating characterization**	Off-line	For modified and non-modified surfaces of metallic implants there are several surface characteristics at the microscale and nanoscale that need to be controlled. For this purpose, a noncontact topography characterization is preferred. However, micrometric and nanometric features should be characterized separately.	Non-contact profilometers such as low coherence interferometer, confocal microscope	✗<br>This process is outsourced, thus minimizes costs and risks for mall productions	✓<br>Due to the larger production volumes this process is more suitable for this company	✗<br>This process is outsourced, the company does not have expertise and experience
**G-16: Detailed periodic random inspection of finished product**	Off-line	Periodic random destructive tests of standard and bespoke components. In the case of bespoke components, they can only be randomly tested if a strong data base is present. Moreover, surface properties of coated and non-coated implants also need to be tested. Some of these properties are roughness, hardness, layer thickness, shear fatigue strength, static shear strength, plastic deformation, and abrasion.<br><br>The tests are static and dynamic mechanical tests that should be performed following the corresponding ASTM standards of each component type.	Fatigue Testing Machine, and universal testing machine, indentation hardness tester, scanning electron microscope, and coating thickness gauges	✗<br>The omission of this process can raise many potential critical risks.	✓<br>This is a great quality control practice	✓<br>This is a great quality control practice
**G-17: Visual inspection of finished products**	Off-line	Comprehensive visual inspection of the final product. The aim is to detect residual errors that could not be detected in previous stages. Here an inspector checks the overall quality of each implant and assembly, including all the product documentation from the previous quality control gates. In this quality control gate, the inspector visually compares each component and assembly with the original design and its specified tolerances. Some of the critical areas to be measured are thread holes, assembly tolerances and movement, and the height and width of each component.	Caliper, micrometer, magnifying goggles, and schematics	✗<br>This process is omitted by this company because is previously performed with a micro-CT scanner, which gives to this company enough confidence.<br><br>However, this process is recommended in order to reduce the probability of a defective product reaching the market.	✓<br>However, this process is recommended in order to reduce the probabilities of a defective product reaching the market.	✓<br>However, this process is recommended in order to reduce the probabilities of a defective product reaching the market.

**G-18: Sterilization and Packaging validation**	Off-line	Validation and routine inspections of Cleaning, disinfection, and sterilization, marking, labelling, and packaging processes, including biocompatibility tests to certify batches. The sterility validation of medical devices at the industrial scale can be performed using a small number of product samples to determine the sterility assurance level (SAL). After validation, the efficiency of disinfection, cleaning, and sterilization processes most be routinely monitored on each cycle. Therefore, during routine production quality engineers must check sterilization certificates, and sterilization indicators.<br>Regarding to the marking, labelling, and packaging of patient-specific implants a visual inspection is required. In this visual inspection it is necessary to verify that each component was adequately marked based on patient information and intended used. Moreover, external package labelling should correspond to it content, and following the corresponding standards. Regarding, the main implant package it is important to inspect it in an exhaustive way to identify potential issues such as punctures, damage, or defective sealing.	Product master record, sterilization certificates, and magnifying goggles	Sterilization process is Outsourced	✓	Cleaning, disinfection, and sterilization, marking, labelling, and packaging processes are outsourced
				This process is outsourced, thus minimizes costs and risks for mall productions	Due to the larger production volumes this process is more suitable for this company.	This process is outsourced, thus minimizes costs and risks for mall productions

Assistance interview documents

The sistance files used in this study were taken from our previous work [12]

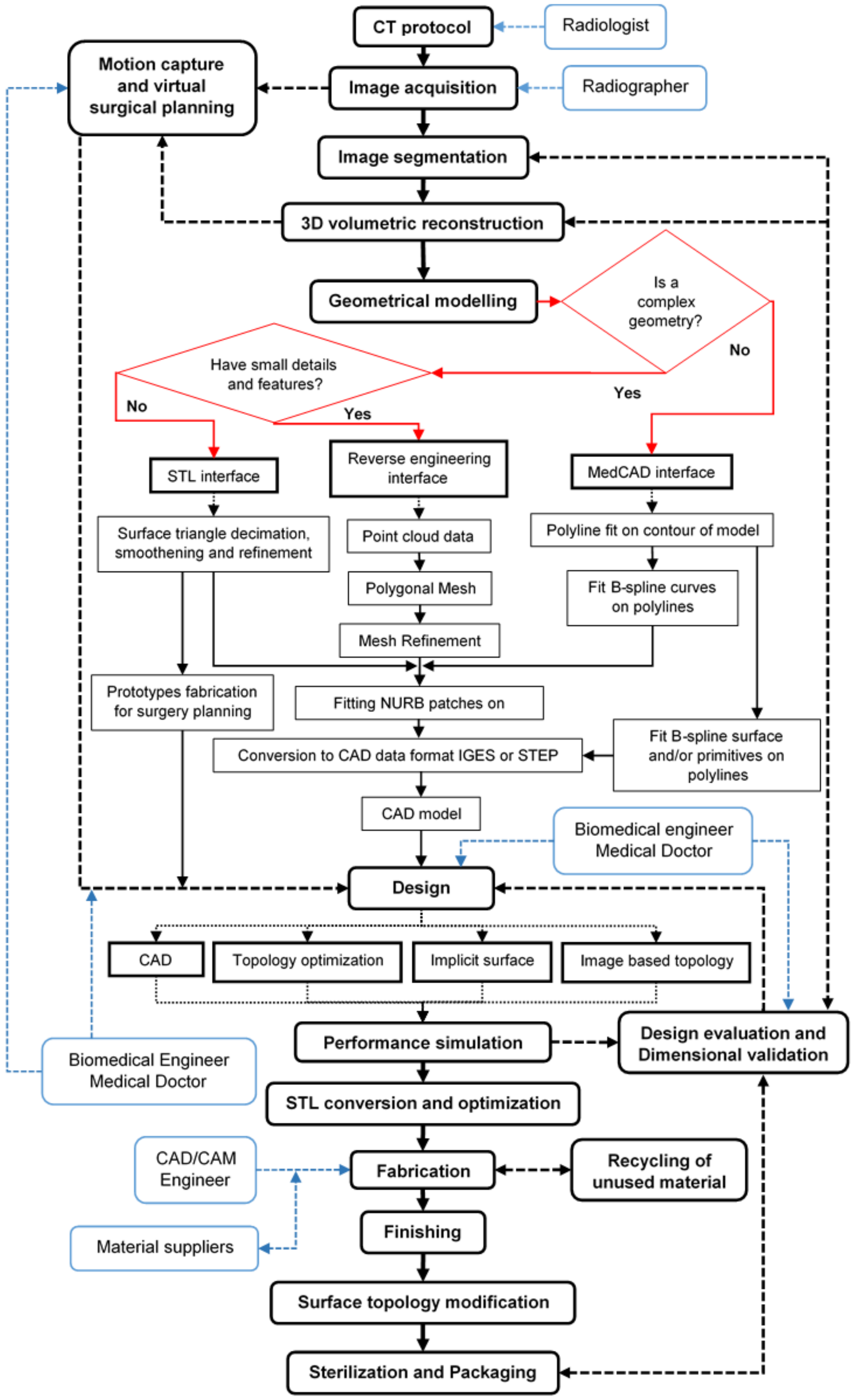

Figure S1. Detailed process flow diagram of custom 3D printed prostheses and scaffolds [12]

materials

Table S1. RBS of the causes of quality non-conformity in custom 3D printed bone prostheses [12]

Level 0	Level 1	Level 2	Level 3		
			Code	Risk	Effect
Non-conformance risks	1. Method	1. CT protocol	1.1.1	Difficulty to implement CT protocol. Use of wrong CT parameters	• Model dimension distortion
			1.1.2	Slice increment is too large or the slice thickness is too big	• Stair step effect<br>• Rough dissolved surface<br>• Fail to capture thin bone (mainly in facial structures such as orbital walls)<br>• Smooths out sharp corners greatly affecting the accuracy of sharp vertices or acute edges.
			1.1.3	Small radiation dose compared to slice thickness	• Noise in images leads to wrong 3D reconstruction
		2. Image acquisition	1.2.1	Wrong gantry tilt	• Distortion of 3D volumetric reconstruction
			1.2.2	Patient involuntary movement during CT scan	• Discrepancies in CT images<br>• Distortion of 3D volumetric reconstruction
			1.2.3	Metallic artefacts	• Distortion of 3D volumetric reconstruction
			1.2.4	Compressed file or wrong file format	• Discrepancies in CT images<br>• Low quality image resolution
		3. Image segmentation	1.3.1	Incorrect thresholding or algorithm processing	• Fail to capture thin bone (mainly in facial structures such as orbital walls)<br>• Larger or smaller models due to wrong boundaries<br>• Noise in 3D volumetric reconstruction<br>• Dimensional variations in the model
		4. 3D volumetric reconstruction	1.4.1	Incorrect mesh generation	• Dimensional variations in the model<br>• Noise in 3D volumetric reconstruction<br>• Loss of data<br>• Fail to capture thin bone
			1.4.2	Incorrect mesh optimization or refinement	• Poor and rough surface quality
			1.4.3	Software used	• Dimensional variations in the model<br>• Noise in 3D volumetric reconstruction<br>• Loss of data<br>• Fail to capture thin bone
			1.4.4	Conversion from DICOM to STL.	• Dimensional variations in the model<br>• Noise in 3D volumetric reconstruction<br>• Loss of data<br>• Fail to capture thin bone
		5. Design	1.5.1	Software: file conversion between STL and CAD	• Loss of part details such as thin bone of the orbital wall, due to incomplete data transfer during file conversion<br>• Loss of thin bone<br>• Floating regions
			1.5.2	Wrong freeform approximation	• Stair step effect
			1.5.3	Wrong implant/scaffold design	• Inclusions of particles inside closed cavities
			1.5.4	Wrong design (surface and unit cell)	• Wrong implant mechanical properties for soft-hard tissue contact adaptation (modulus of elasticity)
			1.5.5	Close tolerances	• Wrong tolerances of the fabricated part due to tolerances being geometry dependent

	1.5.6	Wrong scaffold pore design (unit cell), such as size, shape, and interconnection	• Insufficient cell density and bone regeneration
	1.5.7	Insufficient support structures	• Part or surface damage
	1.5.8	Wrong tolerances	• Fusion of trapped particles
	1.5.9	Incorrect material or design parameters	• Part shrinkage and distortion
	1.5.10	Incorrect part orientation for fabrication	• Stair step effect<br>• Undesired surface quality<br>• Orthotropic or transvers isotropic mechanical behaviour<br>• Dimensional inaccuracy<br>• Bad surface quality<br>• Risk of warping<br>• Can increase fabrication time<br>• Leads to dimensional inaccuracies due to post machining
6. Simulation and FEA	1.6.1	Discrepancies between computational simulation and experimental data, due to inaccurate micro precision in fabrication	• Inaccurate mechanical and biological properties as well as fluid dynamics
	1.6.2	Errors in joint kinematics estimation	• May affect load computations.
	1.6.3	Inaccurate estimation of bone contact forces	• Implant failure
	1.6.4	Inaccurate estimation of bone/implant contact forces	• Inaccurate estimation of micromotion and stability between bone and implant
	1.6.5	Incorrect FEA parameters	• Wrong design<br>• Wrong design optimization<br>• Wrong implant prediction of mechanical behaviour
7. Fabrication	1.7.1	Inaccurate fabrication of micro-features such as pore size and shape	• Reduced biological and mechanical performance<br>• Defective product
	1.7.2	Localised material heating and cooling.	• Thermal warping or dimensional distortion<br>• Residual stress<br>• Material microstructure variation
	1.7.3	Material contamination	• Defective product due to impurities higher than max limits
	1.7.4	Part overhanging features	• Undesirable defects
	1.7.5	Residual polymerization	• Inaccurate parts
	1.7.6	Fabrication layer thickness.	• Stair step effect in Z direction<br>• Material density<br>• Surface roughness
	1.7.7	Large scanning spacing or hatch spacing	• Stair step effect in X and Y directions, leading to dimensional inaccuracy<br>• Insufficient bonding between scan tracks<br>• Poor mechanical properties
	1.7.8	Low scanning spacing or hatch spacing	• Slowdown the fabrication process<br>• Increases amount of energy require<br>• Increases fabrication cost
	1.7.9	Laser diameter	• Omission of part fine details
	1.7.10	High laser temperature	• Part shrinkage<br>• Fusion of surrounding particles<br>• Over-curing or over-sintering

		1.7.11	Low laser temperature	• Slow fabrication process<br>• Increases fabrication cost
		1.7.12	Powder bed temperature	• Variation on part density and mechanical properties<br>• Age hardening of material
		1.713	Material thermal and phase change effects	• Part shrinkage
		1.7.14	Incorrect cooling cycle	• Thermal warping<br>• Changes in colour<br>• Variation in material microstructure.<br>• Damage of unsintered powder
		1.7.15	Part exposition to atmosphere when still at high temperature	• Changes in colour
		1.7.16	Different professional terminologies	• Defective product<br>• Misinterpretation of different terminologies from the different fields involved.
		1.7.17	Process documentation and expression of documents	• Defective product.<br>• Inaccurate product definition<br>• Inefficient workflow<br>• Miscommunication and difficulty to understand different terminologies from the different fields involved
		1.7.18	Communication method (technology used)	• Defective product<br>• Inefficient workflow<br>• Inappropriate interpretation of the transferred knowledge
		1.7.19	Difficulty to monitor fabrication process	• Can leads to reduced quality<br>• Process inconsistency<br>• Unreliability
		1.7.20	Inaccurate/inefficient material recycling method	• In powdered materials this can lead to larger particle size, contamination, and oxidation.
	8. Finishing	1.8.1	Removal of supporting structures	• Part damage<br>• Lead to surface roughness where support structures were allocated
		1.8.2	Part cleaning (Sand blasting)	• Part damage (changes in part dimensions)<br>• Inclusions of particles in porous structures (biological contamination)
		1.8.3	Surface modification method	• Discrepancy in mechanical behaviour
	9. Surface topography modification	1.9.1	Surface topography modification parameters.	• Wrong biological performance<br>• Biofilm formation
		1.9.2	Selection of surface topography modification method.	• Different biological performance<br>• Biofilm formation
	10. Implant dimensional validation	1.10.1	Difficulty locating land marks to measure the model.	• Wrong measurement of model
		1.10.2	Human error during measurement	• Wrong measurement of model
		1.10.3	Dimensional validation method	• Wrong measurement of model
	11. Sterilization	1.11.1	Wrong selection of sterilization method	• Implant dimensional changes<br>• Alteration of surface chemistry and morphology

		1.11.2	Inefficient sterilization	• Biological hazard, such as viral and microbial transmission<br>• Leads to revision surgery
2. Machine		2.1	Building speed variation	• Inaccurate part dimensions<br>• Defective part<br>• Higher cooling rates<br>• Higher material porosity
		2.2	Misalignment of positioning system	• Inaccurate part dimensions<br>• Defective part
		2.3	Part movement during fabrication	• Inaccurate part dimensions<br>• Defective part
		2.4	Clogged print head or Nuzzle	• Damaged or defective part
		2.5	Worn coater blade	• Low surface quality
		2.6	Short of feed powder	• Low surface quality
		2.7	Laser failure	• Internal defects<br>• Undesirable porosities
		2.8	Random errors in parts made by the same process, using the same material and parameters	• No identical implants
		2.9	Localised material heating and cooling	• Thermal warping<br>• Dimensional distortion<br>• Residual stress<br>• Material phase change effects
		2.10	Machine maintenance	• Equipment failure<br>• Inefficiency
		2.11	Machine calibration	• Dimensional inaccuracies<br>• Low quality parts
		2.12	Machine parameters	• Dimensional accuracy<br>• Low quality parts<br>• Unreliable mechanical properties
		2.13	Difficulty to monitor fabrication process	• Can leads to reduced quality<br>• Process inconsistency<br>• Unreliability
3. Personnel		3.1	Poor communication between design team and surgeon	• Inaccurate product<br>• Difficulty to predict surgery risks and results
		3.2	Misinterpretation of the transferred knowledge	• Defective product<br>• Inefficient workflow<br>• Inaccurate product definition
		3.3	Availability of high qualified personal in the necessary professional skills	• Can lead to low performance<br>• Difficulties in product development
		3.4	Material Suppliers	• Low material quality
		3.5	Fabrication/design suppliers	• Low quality
		3.6	Deficient personnel traits and training	• High chances of mistakes during design and fabrication processes, leading to low quality products
		3.7	Lack of training and experience due to introduction of new technologies and surgical methods (medical doctor)	• Inaccurate use of implant<br>• Higher surgery risk
		3.8	Low stakeholders involvement during the	• Incorrect product characteristics

		product development and design process	
	3.9	Limited knowledge of patient's current health condition an biological characteristics	• Poor preoperative planning<br>• Iatrogenic trauma<br>• Concomitant injuries and illnesses<br>• Longer operation time<br>• Increment of revision surgeries<br>• Higher infection rate<br>• Decreased of bone healing rate
4. Materials	4.1	Material stored under sun light and humid places	• Damage material and contamination<br>• Distinct mechanical properties
	4.2	Expired material	• Inconsistent mechanical properties<br>• Inconsistent printing quality
	4.3	Wrong material mix (% of virgin and % of recycled)	• Inconsistent mechanical properties
	4.4	Wrong powder particle size	• Incorrect particle bonding,<br>• Weaken part areas.<br>• Incorrect powder material flowability<br>• Decreases fabrication resolution
	4.5	Wrong material characteristics and contamination	• Inconsistent mechanical properties, not compliance with regulations
	4.6	Wrong material selection	• Reduced implant biocompatibility<br>• Reduced life span
	4.7	Material reuse times	• Can lead to contamination<br>• Increment in oxygen content,<br>• Less spherical and rougher powder particles<br>• Increase in powder flowability, Inconsistent mechanical properties

References

1. McCutcheon, D.M.; Meredith, J.R. Conducting case study research in operations management. *Journal of Operations Management* **1993**, *11*, 239-256, doi:10.1016/0272-6963(93)90002-7.
2. Maylor, H.; Blackmon, K.L. *Researching business and management*; Palgrave Macmillan: New York;Houndmills, Basingstoke, Hampshire;, 2005.
3. Harrell, M.C.; Bradley, M.A. *Data collection methods. Semi-structured interviews and focus groups*; DTIC Document: 2009.
4. Hussey, J.; Hussey, R. *Business research: a practical guide for undergraduate and postgraduate students*; Macmillan: London, 1997.
5. DiCicco-Bloom, B.; Crabtree, B.F. The qualitative research interview. *Medical education* **2006**, *40*, 314-321.
6. Bryman, A. Integrating quantitative and qualitative research: how is it done? *Qualitative research* **2006**, *6*, 97-113.
7. University of Wisconsin. Data collection methods. Availabe online: https://people.uwec.edu/piercech/ResearchMethods/Data%20collection%20methods/DATA%20COLLECTION%20METHODS.htm (accessed on 18th of December).

8. Tong, A.; Sainsbury, P.; Craig, J. Consolidated criteria for reporting qualitative research (COREQ): a 32-item checklist for interviews and focus groups. *International Journal for Quality in Health Care* **2007**, *19*, 349-357.
9. Phillips, P.; Phillips, J.; Aaron, B.; Books24x, I. *Survey Basics: A Guide to Developing Surveys and Questionnaires*; American Society for Training & Development: 2013.
10. Sarica, A.; Guzzi, P.H.; Cannataro, M. Building and mining web-based questionnaires and surveys with SySQ. *Interdisciplinary Sciences: Computational Life Sciences* **2013**, *5*, 233-239, doi:10.1007/s12539-013-0167-8.
11. Eisenhardt, K.M. Building Theories from Case Study Research. *The Academy of Management Review* **1989**, *14*, 532-550, doi:10.5465/AMR.1989.4308385.
12. Martinez-Marquez, D.; Mirnajafizadeh, A.; Carty, C.P.; Stewart, R.A. Application of quality by design for 3D printed bone prostheses and scaffolds. *PLOS ONE* **2018**, *13*, e0195291, doi:10.1371/journal.pone.0195291.
