# Supplementary material for: Development of 18 Quality Control Gates for Additive Manufacturing of Error Free Patient-Specific Implants
Source: Materials (Basel). 2019 Sep 24;12(19):3110. doi: 10.3390/ma12193110 (PMC6803939; doi:10.3390/ma12193110)
Supplement: Supplementary file 1 [file materials-12-03110-s001.zip › S2_FILE-1.pdf]

materials MDPI

Supplementary Material S2

Practitioner companion guide: Development of 18 quality control gates for additive manufacturing of error free patient-specific implants

Daniel Martinez-Marquez [a], Milda Jokymaityte [b], Ali Mirnajafizadeh [c], Christopher P. Carty [d,e], David Lloyd [d], and Rodney A. Stewart [a*]

[a]*School of Engineering, Griffith University, Gold Coast, QLD, Australia*
[b]*Ortho Baltic, Kaunas, Lithuania*
[c]*Molecular Cell Biomechanics Laboratory, University of California, Berkeley, CA, USA*
[d]*School of Allied Health Sciences and Gold Coast Orthopaedic Research and Education Alliance, Menzies Health Institute Queensland, Griffith University, Gold Coast, QLD, Australia*
[e]*Department of Orthopaedic Surgery, Queensland Children's Hospital, Children's Health Queensland Hospital and Health Service, Brisbane, QLD, Australia*

* Corresponding author. Tel.: +61 (7) 5552 8778
E-mail address: r.stewart@griffith.edu.au

Note: Reference this document same as the main article

Integrated quality control Flow chart

Multistage manufacturing systems, such as the ones used to produce patient-specific implants, are composed by multiple production processes that require a delicate coordination to obtain the final product. The overall performance of these type of systems depends on the accumulated performance of their stages [1]. Therefore, error propagation is the major cause that affects the overall system performance of multistage manufacturing systems [2]. According to Hrgarek [3] the cost of fixing an error increases exponentially when it moves forward through the product development cycle and production chain [3]. This cost is even higher in the medical industry, because a defective product can represent a life-threatening risk and lead to product recalls with serious financial implications such as liability costs and market capitalization loss due to negative brand image [3-5].

Quality control is composed of several processes designed to effectively monitor and prevent quality issues, in order to help to achieve the necessary process performance and product quality standards [6]. In our previous work we identified that through the design and manufacturing processes of patient-specific implants, a total of 85 causes of risks of non-conformance quality [7]. These potential risks of non-quality conformance are mainly caused by the novelty of additive manufacturing (AM) technologies, product geometrical complexity, material properties, and the great variability of customers' needs.

Controlling the quality of this type of product is a difficult task. This is due to fact that patient-specific implants are one-off design products that require higher quality standards than traditional implants, leaving no space for uncertainty. Moreover, the large variation of product characteristics of patient-specific implants increases the probability of human errors due to the reduced opportunity for learning from repetitive operations [2]. Therefore, the quality control activities for the design and fabrication of patient-specific implants should take place in the most sensible activities of the design and fabrication process. This is not a rare practice in many discrete manufacturing process, where total inspection at each intermediate operation are commonly performed [1].

To overcome the above challenges, this study explored the quality control methods employed within three different companies to select the best quality control practices and develop an integrated quality control flow diagram for patient-specific implants. The selection of best practices was based on the quality performance of each company, including their internal quality management culture, and technologies used. The integrated quality control flow diagram was also developed taking into account the FDA guideline: "*Technical Considerations for Additive Manufactured Medical Devices*" [8] and ASTM standards, including the following assumptions:

- Mass production with AM is performed.

- It is assumed that a biocompatibility assessment was previously performed following the ISO 10993 standard.
- The aim is to achieve the highest production performance and customer quality ratings, pursuing the 6σ rating.
- Defective products are unacceptable, due to the potential high risks that they represent to the company, the customer, and the patient.
- It is assumed that missed flaws in the final product have serious consequences to the patient ranging from injury to fatality.
- Product external failure costs and penalty cost are much higher than a quality inspection cost. Therefore, they should be avoided in any instance.
- The scrap and rework costs, and penalty risks should stay at a minimum level.
- The company employees should embrace a proactive quality culture, similar to the one promoted in Total Quality Management
- Inspections are only performed by highly qualified personnel.
- If defects are not detected in a quality control gate they should be detected in the following gate.
- The minimum quality management system in place should be ISO 13485.
- A detailed risk identification and a failure mode analysis should be previously performed.

The resulting quality control workflow chart presented in Fig 1 considers the entire design and production cycle of patient-specific implants. It focusses in preventive quality control activities from a conservative approach. This integrated quality control flow diagram is composed by 18 go/no-go quality control gates that take place before, during, or after the most sensible processes and activities depending on their criticality, and availability of quality control technologies. Go/no-go gates means that the corresponding product quality attributes at each stage must be satisfied in order to continue the following process [6]. Each quality control gate must have its corresponding product validation documentation containing checklists, and control diagrams to track/trace product quality variations at each stage.

According to the Pareto principle, decisions made during product planning and design phases are responsible for approximately 80% of the product final costs [9,10]. Therefore, 28% of the proposed quality control gates of this study were strategically allocated in the product design phase, which can be divided into four different sub-phases: (1) Information design phase; (2) Conceptual design phase; (3) Preliminary design phase; (3) Detailed design phase [11]. From all of the 18 proposed quality control gates, 66.6% are *on-line inspections*. *On-line inspections* are part of the flow process of the design and production line [12]. They are performed during production to catch quality variations caused by careless workers, maladjusted and uncalibrated machines, and environmental conditions [13]. This type of inspection is also aimed to control and ensure that the quality requirements of incoming materials and semi-finished/finished products are met before an value-adding operation is undertaken [13]. The remaining 33.4% of the quality control gates correspond to *off-line inspections* performed by specialised quality inspectors. These are more detailed and time-consuming inspections that interrupt the process flow. However, they are more effective than on-line inspections [12]. The following paragraphs present a detailed description of each quality control gates (G) of the proposed quality control workflow chart developed in this study.

materials
MDPI

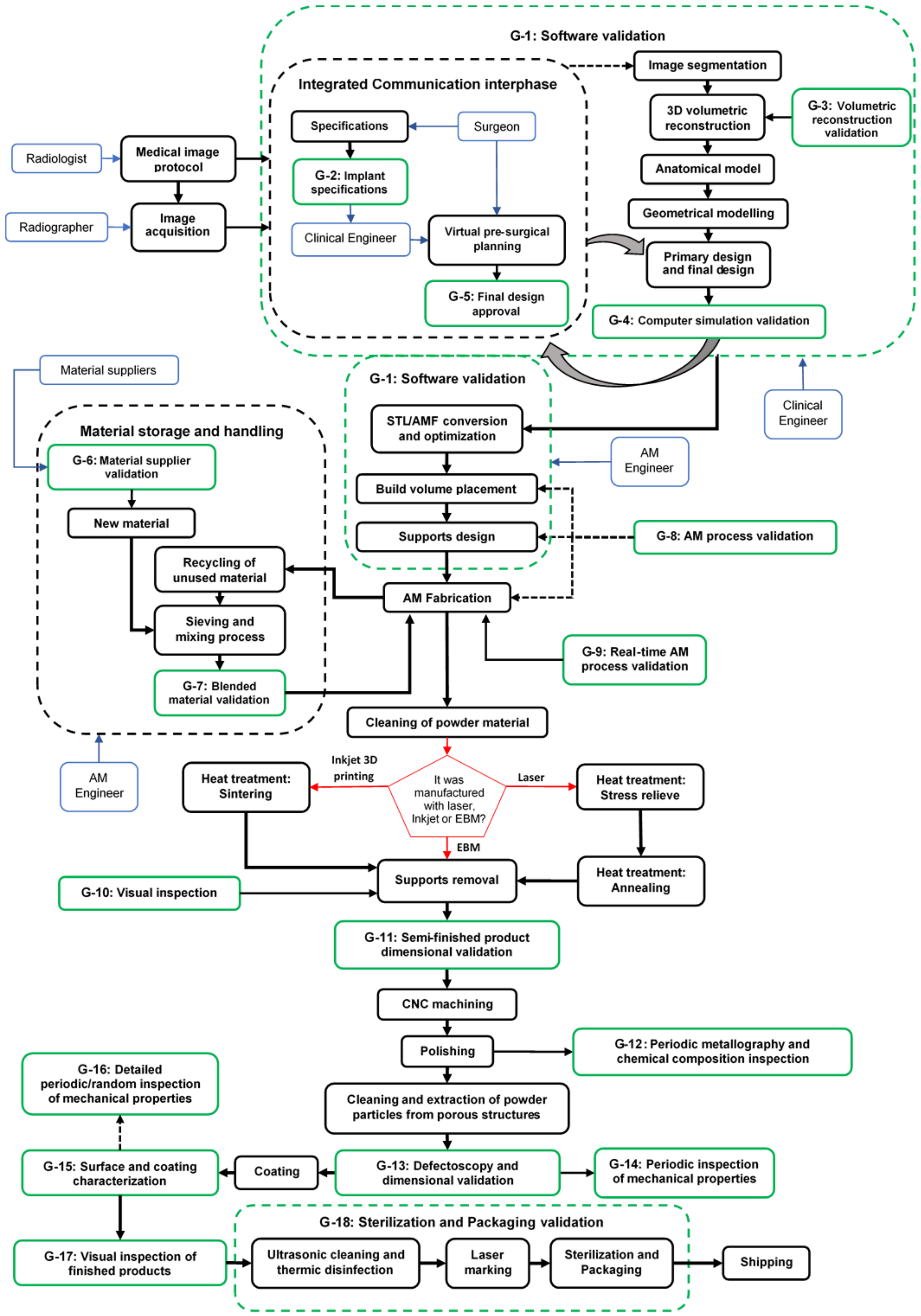

Figure 1. Integrated quality control workflow chart with 18 gates. The meaning of the items in the chart is as follows: Black solid outlined box = process; Black dashed outline box = overarching Process; Green solid outlined box = quality control gate; Green dashed outline box = overarching quality control gate; Red pentagonal box = decision; Blue box = expert/staff; Solid arrow = on-line process; Dashed arrow = off-line process.

G-1: Software validation

Acting as a global quality control gate, G-1 is designed to ensure the reliability of the software workflow and each of the software packages used during the design and fabrication processes of patient-specific implants. This is required because critical data and files are manipulated and shared during the design and fabrication processes where potential imperceptible software errors can occur affecting the quality of the final product. Therefore, before starting to produce patient-specific implants, it is imperative to perform a software workflow validation process to ensure expected performance [8]. The validation of the workflow software requires that the device manufacturer ensures that each software used during the workflow process are appropriate for its intended used. Moreover, according to FDA [14], documentation containing fully defined requirements and testing results should be supplied as evidence to show that each software and the software workflow process are validated. Moreover, the ISO/ASTM 52915 standard recommends the use of Additive Manufacturing File format (AMF). The new AMF file format has higher geometry fidelity than the traditional STL file. Moreover, the AMF file format acts as a document control system that is able to store objects and vital information such as location in a build volume and material information [15].

G-2: Implant specifications

In the production of highly customized products, one of the main causes of producing obsolete components is poor communication between customer and product designers. According to Bordat, *et al.* [16], to achieve error-free products implicates better communication methods, between the design team, the customer and the integration of those with a properly planned design process. Concurrent engineering telecommunication software packages can help to reduce communication errors with real time information exchange and adequate protocols [2]. Therefore, G-2 takes place in an integrated concurrent engineering web software tool.

During this study it was found that two of the studied companies use their own online communication interphase to communicate and interchange information between the surgeon and a company's clinical engineer. Through this interphase the most suitable medical image protocol is decided, and the necessary surgery requirements, patient's information, and implant specifications are collected and corroborated in a systematic way before proceeding to the next steps of the workflow. Some of the necessary information includes the type of surgery to be performed including its risks and constraints, implant's and/or surgical guides' requirements, and patient's medical data such as medical images, description of pathology, and adjacent conditions. Moreover, a concurrent engineering interphase is used within this software to collaboratively plan each surgery. Surgery planning is performed in a 3D environment which simulates the placement of surgical guides, implant, and screws in the patient's anatomy. This facilitates the identification of issues and allows the surgeon to be familiar with the whole procedure.

G-3: Volumetric reconstruction validation

The image segmentation process of the patient's region of interest, is performed to isolate all the different tissues such as muscle, bones, ligaments, cartilage, blood vessels and nerves in order to have an approximated tissue differentiation. However, the high sensitivity of these processes, coupled with the need for in-depth anatomical knowledge, increases the probability of human error, which ultimately could lead to an inaccurate implant design.

To prevent and control the potential errors of this process the G-3 is performed to validate the image segmentation process and the 3D volumetric reconstruction. This quality control process requires comparison of the 3D volumetric reconstruction of the patient's region of interest directly with the original medical images from the patient (computer tomography scan or magnetic resonance tomography). If errors are found during this process, they can be corrected by repeating the image segmentation process. Then if the 3D reconstruction process is satisfactory, the 3D volumetric reconstruction is used as a template to design the implant, and to create an anatomical model of the region of interest to assist the pre-surgical planning process.

G-4 and G-5: Computer simulations and final design approval

G-4 is usually carried out by the studied companies at least two times during the overall design process, one after the primary design process and after the final design approval. After the primary design process, the implant and its corresponding surgical guides have already been designed to match the geometry of the patient's region of interest. However, the mechanical properties and dynamic performance of the implant need to be tested according to the specific conditions of the patient and correspond to medical standards. For this purpose, a virtual non-destructive testing of the implant design is usually performed with a finite element analysis (FEA) software package to guarantee the implant

mechanical integrity, as well as to optimise its weight. For this purpose, companies refer to the recommendations of ASTM standards for FEA related to the type of implant that is been design. Examples of these standards are presented in Table 1. However, according to the clinical engineers of the studied companies, currently there are no standard guides or recommendations for the external forces and boundary conditions for FEA of patient-specific implants. Therefore, these companies use the external forces and boundary conditions published in literature and also from muscle skeletal models developed in collaboration with universities research groups.

Knowing that the future changes in medical device regulations will make more stringent the requirements to register and commercialize patient-specific implants, it is vital that companies in this sector to proactively be prepared. It is already known that regulatory changes will force manufacturers and designers of patient-specific medical devices to provide sufficient clinical data and clinical evaluation before the registration of a device, in order demonstrate its clinical performance and benefits [17]. Therefore, to in order to be comply to future challenges that new regulations are imposing it is vital to not just design implants based on the unique 3D geometrical features of patients' bone. Instead, it is proposed that patient-specific implants should be designed taking into consideration joint contact forces that occur during real life activities. For this purpose, a 4D implant design approach, as described in our previous work [7], should be used to help to validate patient-specific implants with patient-specific computational neuromusculoskeletal (NMS) predictions and multiscale finite element analysis (MFEA). This approach also permits medical practitioners to understand the mechanisms of injury and disease of the musculoskeletal system and structural form-function relationships to better design, test, and validate implants [7]. The computational NMS considers the muscle force contributions to estimate the in vivo contact loads of the patient's region of interest. These muscle forces are affected by external loading conditions, joint kinematics, as well as an individual's task-specific muscle activation patterns, during real life activities. Furthermore, MFEA enables virtual tests and simulations for more insightful study, design and optimization of micro and nanostructured hierarchical materials. These simulate the interactions of different implants microstructures, surfaces, and tissues [7].

Furthermore, according to the AM engineers of the studied companies another aspect to consider when components are fabricated with DMLS and Inkjet 3D printing are the resulting residual stresses and part distortion caused during the fabrication process. These residual stresses and part distortion are caused by large thermal gradients during the metal sintering process [18]. Consequently, to reduce the number of trials to make a reliable product they also run thermo-mechanical simulations to predict residual stresses and product distortion [19]. With the results of these simulations the AM engineers then can design strategies to counteract and mitigate these issues [20].

Following the simulation process, the virtual pre-surgical planning is performed with an interactive communication between the surgeon and the clinical engineer using the integrated concurrent engineering web software tool. Sometimes during the pre-surgical planning process, it can be found that the preliminary design needs further refinement which leads to several design iterations before reaching the final design. When this process is satisfactory, the changes on the design are implemented and the G-4 should be performed once again as a final simulation. The result of this final simulation is a detailed engineering report describing worst case scenarios, critical loads and critical implant areas, and parameters for an optimum fabrication process and heat treatments.

G-5 takes place as a final design approval. Here the surgeon is asked to fill out and sign the presurgical planning protocol to confirm that the surgical procedure plan, the patient-specific implant design and its corresponding surgical guides are suitable for the patient. The result of this procedure is a detailed planning report of the preoperative situation, which includes the characteristics of the implant and the expected postoperative situation to be achieved.

a) b)

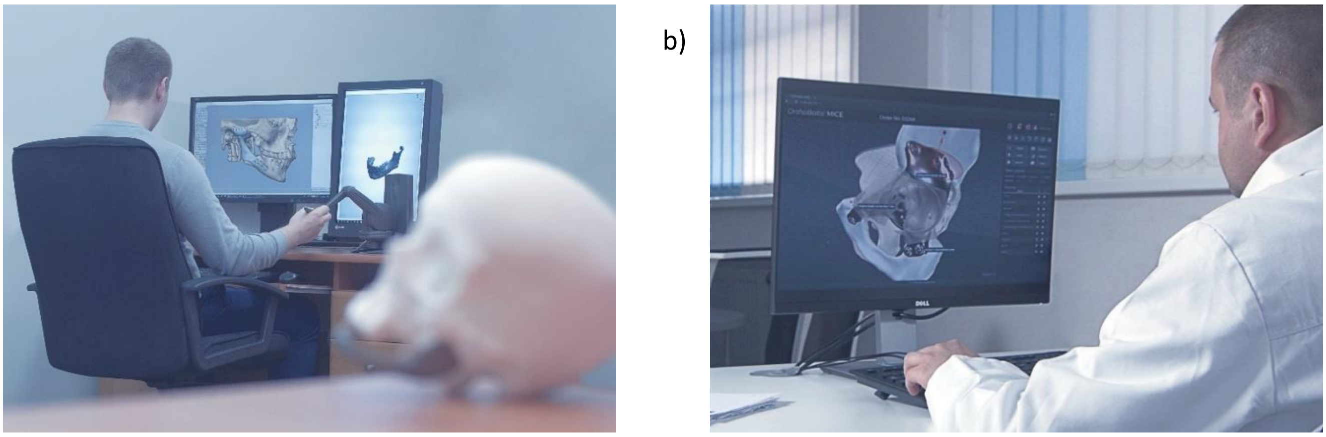

Figure 2. (a) Implant design and (b) surgery planning performed with the surgeon-manufacturer communication tool MICE (Medical Implants Customization Engine).

Table 1. Examples of FEA ASTM standard test for orthopaedic components [21-23].

Standard designation code	Standard	Description
**ASTM F3161-16**	Standard Test Method for Finite Element Analysis (FEA) of Metallic Orthopaedic Total Knee Femoral Components under Closing Conditions	This standard can be used to calculate stresses on knee femoral components. Moreover, it can be used to identify the worst-case size for a particular implant family.
**ASTM F2996-13**	Standard Practice for Finite Element Analysis (FEA) of Non-Modular Metallic Orthopaedic Hip Femoral Stems	This standard can be used to calculate stresses on a femoral hip stem when loaded in as described in ISO 7206-4 (2010). Moreover, it can be used to identify the worst-case size for a particular implant family.
**WK59162**	New Test Method for Finite Element Analysis (FEA) of Metallic Orthopaedic Total Knee Tibial Components	This is a work-in-process standard that aims to establish requirements and considerations for the numerical simulation of metallic orthopaedic total knee tibial components for the estimation of stresses and strains, but it does not include the prediction of fatigue strength. Moreover, it can be used to identify the worst-case size for a particular implant family.

G-6 and G-7: Material supplier validation and Blended material validation

All the AM systems used by the studied companies use material in powder form to fabricate patient-specific implants and surgical guides. This powder material can be virgin or blended. Powder material is considered virgin if this comes from a single powder lot. Whereas a powder blend is the result of the combination of different powder lots, including recycled powder [24]. However, regardless if the power is virgin or blended it has to follow the same strict specifications, in order to produce consistent high-quality parts with predictable mechanical and chemical properties [25]. Therefore, G-6 and G-7 identified in this study act as the material control and handling process validation.

G-6 is used with the purpose of controlling the quality of the powder material that comes from the material supplier. According to each AM equipment supplier, to achieve the highest performance of their specific AM system it is necessary to use validated powder material, which is strictly supplied by them [26]. This is because material from different vendors might have different characteristics which can significantly impact negatively the properties of additively manufactured parts [27]. However, regardless who is the supplier of the powder material, the supplier must have a recognized quality management program such as ISO 9001, AS9100, or ISO 13485 [24]. Moreover, suppliers should provide material data sheets and certificates with testing results of the material properties.

G-7 is performed to guarantee the physical and chemical characteristics of virgin and blended powder. For this purpose, first it is needed to characterize the metal powder to control its characteristics, such as particle size distribution, flow rate, particle shape, tap density, oxygen content, and hydrogen content [25]. Moreover, metal powder should have a chemical composition within the established limits required by the ASTM standards and component purchaser, and be free from inclusions and impurities [24]. For more details refer to Table 2.

To control some of the powder characteristics, different systems for powder handling and sieving are offered by AM equipment suppliers. Nevertheless, we identified that to further improve the reliability of these type of systems, one of the studied companies uses a sealed room with controlled atmospheric conditions to process their powder material. This is due to the fact that titanium alloys such as Ti6Al4V can easily absorb water, oxygen, and hydrogen from the surrounding atmosphere during the AM fabrication, recycling process, and storage affecting the density and flow rate of the powder particles, and even the chemical composition of the final part [28,29]. Furthermore, according to medical regulations such as the FDA 21 CFR Part 820, ISO 13485, and to the FDA "*Technical Considerations for Additive Manufactured Medical Devices*" every process of the production chain shall be validated. Therefore, at the G-6 and G-7 it is essential to have established procedures and data documentation to facilitate material traceability, and to ensure that all purchased materials and blends conform to the specified requirements by the AM equipment supplier and corresponding standards.

Table 2. Recommended standard tests for characterization of titanium alloy Ti6Al4V powder used for AM, and required material chemistry composition for Powder Bed Fusion systems [24,25].

Recommended tests				Related ASTM standard test methods
Sampling procedures for metal powder				B215
Density				B923, B212, B329, B417, B703, B417, B527
Flow characteristics				B964, B213, B213, B855, B964,
Chemical composition	**Element**	**min**	**max**	E1447, E1941, E1409, E2371, E539, E572, E2465, E2594, and E2626
	Aluminum	5.5	6.75	
	Vanadium	3.5	4.5	
	Iron	-	0.3	
	Oxygen	-	0.2	
	Carbon	-	0.08	
	Nitrogen	-	0.05	
	Hydrogen	-	0.015	
	Yttrium	-	0.005	
	Other elements, each	-	0.1	
	Other elements, total	-	0.4	
	Titanium	remainder		
Morphology characterization				B243
Particle size				B214, B822

G-8: AM process validation

For cost-effective efficient machine volume utilization in AM production, companies produce multiple parts in a single setup. To maximize production, it is required the use of skilled technicians and specialised AM software to optimize the nesting process within the build volume [30]. Nonetheless, there are a variety of potential quality risks that can occur during the nesting process that can affect implant's critical quality attributes such as material properties, dimensional accuracy, and biological safety [7]. To prevent some of the potential risks the factors that need to be taken into account are: part building orientation; part location within the build volume; separation between parts; support structures; AM machine settings; including some specific issues related to the type of AM system used [31,32]. Consequently, an annual validation of the AM process that links machine-process and nesting parameters with part mechanical properties, and more general dimensional and shape-related metrological parameters must be conducted as the G-8.

As best machine validation practice, we identified that Company B use test coupons, and components with similar geometries and features such as trabecular/lattice structures like the ones present in patient-specific devices. These test coupons and components are used as representative test samples to challenge the complete build volume of the fabrication chamber of the AM machine [8], as shown in Figs 3a. This practice allows verification of the correct functioning of the AM machine through the identification of the relationship between material properties of coupons and final products. Moreover, challenging the AM machine allows identification of the worst-case scenarios and process limitations in relation to machine conditions, part placement and geometry [33], as shown in Fig 3b. The result of this validation process is the establishment of the final process parameters that produce consistent and repeatable products that fulfil the required specifications.

Coupons may be tested using destructive and non-destructive standard methods to verify that the dimensional accuracy, mechanical properties, porosity, chemical composition, and material microstructure are within the required quality standards and specifications, as listed in Tables 3. Moreover, according to FDA [8] the data collected during this validation process should be accordingly documented to conform existing guidelines for device validation.

materials

MDPI

Table 3. Example of minimum requirements of mechanical properties, final material permissible variation composition, and related standards of titanium alloy Ti6Al4V components produced with Powder Bed Fusion systems [24].

Recommended tests				Required values	Related standards practice and test methods
Surface texture				Decided by manufacturer	ASME B46.1
Component Density				99%+	ASTM B311
Material Porosity				Less than 2%	ASTM B311
Microstructure					AS1814, ASTM B600, ASTM E3, ASTM E407
Mechanical properties	Hardness				ASTM E10, ASTM E18, ASTM E384, ISO 6506-1, ISO 6507-1, ISO 6508
	Fracture toughness				ASTM E1820, ASTM E399, ASTM E23
	Compression strength				ASTM E9
	Shear				ASTM B769
	Fatigue crack growth				ASTM E647, ISO 12108,
	Fatigue				ASTM E466, ASTM E606, ISO 12108, ISO 1099
	Ultimate tensile strength (UTS)			$\sigma_{max\ x}$ = 895 MPa<br>$\sigma_{max\ y}$ = 895 MPa<br>$\sigma_{max\ z}$ = 895 MPa	ASTM E8/E8M, ISO 6892
	Yield strength (YS) at 0.2 % offset			σ_x = 825 MPa<br>σ_z = 825 MPa	
	Elongation in 5 cm or 4D X,Y and Z direction			10%	
	Reduced area X, Y and Z directions			15%	
Chemical composition	**Element**	**min**	**max**	**Permissible variation in check analysis**	
	Aluminum	5.5	6.75	±0.04	
	Vanadium	3.5	4.5	±0.15	
	Iron	-	0.3	±0.10	
	Oxygen	-	0.2	±0.02	
	Carbon	-	0.08	±0.02	
	Nitrogen	-	0.05	±0.02	
	Hydrogen	-	0.015	±0.002	
	Yttrium	-	0.005	±0.0006	
	Other elements, each	-	0.1	±0.02	

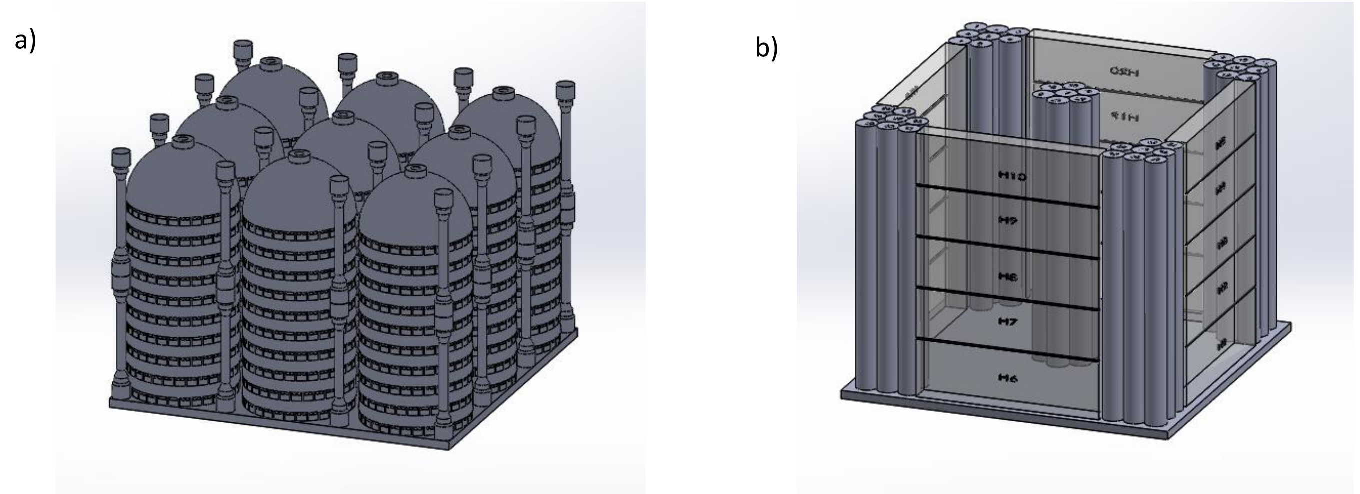

Figure 3. a) Text coupons and representative geometries to challenge the complete build volume; b) Representative sampling to evaluate extremes of range for worst case machine conditions.

G-9: Real time AM process monitoring

G-9 is a real-time process monitoring of the AM process. This is essential for self-regulating process control [34]. This practice is commonly used in traditional manufacturing processes to further guarantee the long-term reliability of industrial systems, allowing to reverse the effects of disturbances and faults during manufacturing [35]. Nevertheless,

in-process monitoring still limited for AM processes [36,37], because this is a relatively young industry. Furthermore, the large number of machine parameters that need to be controlled makes this task extremely challenging [38].

Some of the machine parameters that need to be monitored in a AM machine are: laser or electron beam power, and diameter [39,40]; scanning speed [41]; layer thickness; hatch spacing [42]; bed temperature; cooling cycle [43]; chamber temperature, atmosphere, and pressure [44]. The variability of machine parameters during the additive manufacturing process may cause changes to the material microstructure and chemical composition [41]. Moreover, material fatigue strength can be strongly affected when voids and pores are developed during fabrication [40]. Additionally, during fabrication a nonuniform heat transfer and uncontrolled cooling cycles leads to residual stresses and dimensional distortion of the manufactured component affecting its dimensional accuracy and fatigue crack growth [45,46].

There are just a few commercial monitoring systems available for AM. Some of them are currently used by the companies in this study. For example, the EBM system Q10plus Arcam offers two process validation systems LayerQam for defect detection and xQam for machine autocalibration. The LayerQam is an inline camera-based quality verification system for in-situ process monitoring. This system is composed of an image processing algorithm, and a high-resolution camera that works with in visible light and near-infrared region (NIR) to analyse the melt pool for defect detection. This monitoring system takes pictures of every layer to monitor porosities created during the melt process to verify 100% density of produced components. Furthermore, the system generates a post-build report, and the pictures captured during the building process can be staked in a similar way as a CT scan to create a 3D image of the entire built [47,48]. On the other hand, the xQam system is a real-time high precision autocalibration system which compromises an X-ray detection system and a powerful software platform. The xQam system allows a better electron beam control to improve focus accuracy and eliminates the need for manual calibration, reducing the process from 4 hours to just 15 minutes [49].

The DMLS EOSINT M 280 machine comes with an EOSTATE which is a status control and reporting software that works together with several sensors to permanently monitor and record in-real time the laser power, cooling system, build platform positioning, electrical system, build platform temperature, ambient temperature, air circulation system, oxygen concentration, dispensing system and collection vessel, including a scanner self-calibration. Moreover, if the system detects deviations out of a pre-defined range, it stops the fabrication process to avoid further fabrication issues [50].

Nevertheless, to further improve machine monitoring of DMLS machines EOS recently launched EOSTATE process monitoring suite that consist of four modules developed to monitor in real-time the intrinsic characteristics of the building process [51]. The first, two monitoring systems are EOSTATE MeltPool and EOSTATE Exposure OT together, both systems can detect variations in the scan speed, hatch distance, and laser power before the creation of part defects. The EOSTATE MeltPool determines process deviations through two photodiodes that measures the light emitted in the near-infrared spectrum from the entire build platform and the melt pool. Whereas, the EOSTATE Exposure OT is a camera based system that works in the NIR wavelength range to monitor the entire build platform [52]. The third real-time monitoring system is EOSTATE PowderBed. This system consists in an integrated 1.3 Megapixel industrial camera that monitors the powder bed taking pictures after each coating and laser exposure to identify irregularities such as cavities, incomplete powder layer, and grooves [53,54]. Additionally, EOSTATE can create a detailed report of the building process, and independently selected components containing machine data and statistics [55].

To acquire a customisable control of 3D printing laser systems, there are commercial solutions such as Materialise Control Platform & Inspector by Materialise, and PrintRite3D by Sigma Labs. Materialise Control Platform & Inspector is a modular software-driven, embedded hardware solution an a machine calibration tool that enables total control of 3D printing machines parameters reducing fabrication time, facilitating quality monitoring and control, and allowing better process repeatability [56]. This system has an image processing software that can detect recoater failure, warping, tear, and the quality of the melt pool. Furthermore, it provides an energy density map that can be used in combination with a simulation of the fabrication process to investigate behaviour of the AM building process helping root cause analysis, and to optimise the manufacturing process [57]. PrintRite3D, is a group of software modules for real-time process control and quality assurance for laser-based AM. The software algorithms were specifically designed to work in conjunction with multi-sensors and hardware offered by Sigma Labs to measure the melt pool thermal energy density with a resolution down to 100 microns [58]. The multi sensor pack comes with two photodetectors, a high-speed, single wave length pyrometer, and a sensor to collect signals from the scan head controller [59]. There are two software modules available the PrintRite3D INSPECT and the PrintRite3D CONTOUR. PrintRite3D INSPECT is for in-process inspection for metal AM that uses statistical analysis of the manufacturing process to produce part quality reports. Whereas, PrintRite3D CONTOUR is a real-time monitoring software for geometry checking. This is done by edge detection algorithms that verifies geometry during the building process layer by layer, allowing to compare the original build part with the CAD model [58].

Table 4. Summary of commercial monitoring systems for AM

In-monitoring system	Modules	Developer	AM system	Failure mode monitored	Equipment
Materialise Control Platform & Inspector		Materialise	SLA, DMLS, SLS	Complete control of the machine parameters, geometrical calibration of scan field, prediction and control of fabrication errors, optimization of slicing algorithms and toolpath	• Software and hardware for control of AM machine
PrintRite3D	PrintRite3D INSPECT<br><br>PrintRite3D CONTOUR (in development)	Sigma labs	DMLS	Melt pool energy density	• Two photodetectors<br>• A high-speed, single wave length pyrometer<br>• Sensor to collect X and Y command signals from the scan head controller
EOSTATE monitoring suite	EOSTATE basic	EOS	EOSINT M 280	Laser power, cooling system, build platform positioning, electrical system, build platform temperature, ambient temperature, air circulation system, oxygen concentration, dispensing system and collection vessel, laser scanner self-calibration.	• Set of different sensors across the entire machine (no detailed information found)
	EOSTATE PowderBed	EOS	EOS M 290	Cavities, incomplete powder layer, and grooves	• Integrated 1.3 Megapixel industrial camera
	EOSTATE MeltPool	EOS	EOS M 290	Melt pool intensity profile	• Two off-the-shelf photodiodes
	EOSTATE Exposure OT	EOS	EOS M 290	Scan speed, hatch distance	• Optical tomography: NIR camera with resolution of 2560 x 2160 pixels
LayerQam		Arcam	Arcam Q10plus	Melt monitoring and porosity	• NIR high-resolution camera
xQam		Arcam	Arcam Q10plus	Electron beam autocalibration	• X-ray detection system for electron beam autocalibration

G-10: Visual inspection

G-10 is an on-line visual inspection of the surface quality and dimensional deviations of semi-finished products. Some of the dimensional variations are caused during the rapid heating and cooling cycles during fabrication. To control these dimensional changes, after fabrication different heat treatments are performed depending on the type of AM system used. However, during the processes of detachment from the build platform, and removal of support structures (Fig 4) also dimensional variations and visible surface marks could be introduced. Therefore, at this quality control gate, the technician in charge of these activities is also in responsible of inspecting each component.

It is well known that known defects of repeatable location are detected most successfully [12]. Consequently, proper training and experience play an important role in the performance of this task. If surface marks and dimensional deviations are encountered during in G-10, they should be reported in the master record of each component to take a closer analysis in the following quality control gate.

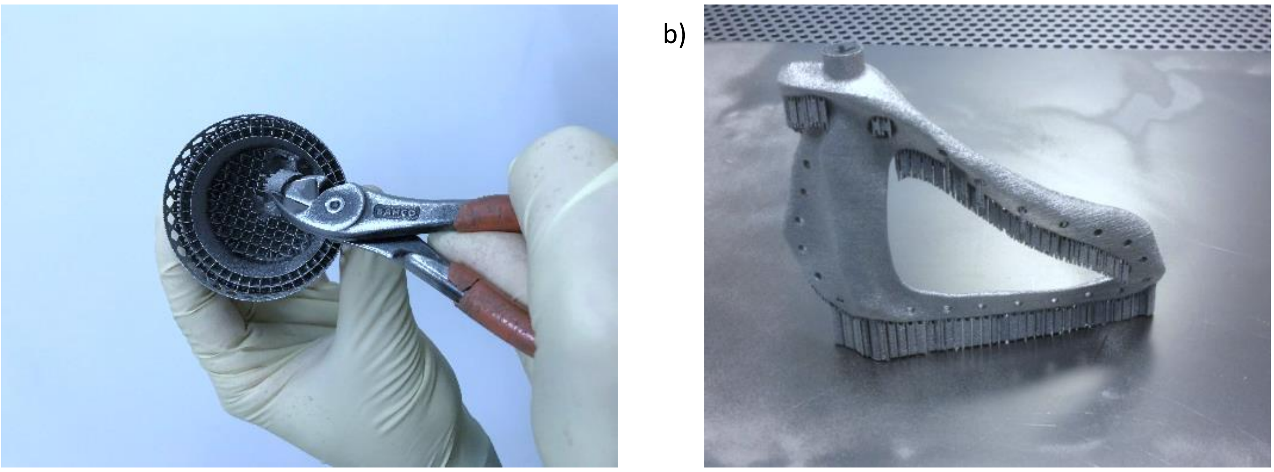

Figure 4. a) Removal of support structures of a component fabricated with an EBM system; b) A patient-specific scapula implant with support structures fabricated with an EBM system.

G-11: Semi-finished product dimensional validation

G-11 is a rapid but detailed dimensional validation of the semi-finished components. This quality control gate is required to identify dimensional deviations caused during the AM fabrication process. Some of these deviations can be caused by internal stresses when AM systems such as DMLS and Inkjet 3D printing are used [45,60,61]. These stresses are simulated in G-4 counteracting their effects with different heat treatments such as stress relief and annealing [62]. Nevertheless, computational simulations of complex objects are difficult to perform, so accurate predictions are not always obtained. Therefore, in this quality control gate the dimensional validation of components is performed by an expert that compares each component with the original design and its specified tolerances using basic measurement tools such as caliper and micrometer, as shown in Fig 5. Some of the critical areas to be measured are holes for future threads, and the height and width of the component using predetermined landmarks. Product quality deviations in this process are compared with pre-specified tolerances and categorised according to its criticality based on a ranking criterion. Then depending on the score obtained it is decided if the component is rejected or if requires a more detailed dimensional inspection. It is important to highlight that this type of quality control activity is highly sensible due to its subjective nature and dependency on expert knowledge and experience [63].

However, sometimes the geometrical complexity of freeform shapes of patient-specific implants does not allow to perform accurate metrological measurements using traditional tools such as the calliper and micrometre [64]. In this case a more detailed dimensional inspection is required. In this detailed dimensional inspection, a high-resolution point cloud is obtained by combining a contact coordinate measurement machine (CMM) and a 3D laser scanner improve measurement resolution and speed of both systems. The 3D object obtained from the combined point cloud is compared with the original design to generate a deviation map that allows the measurement critical component sections such as holes for future threads, spherical surfaces, and bearing surfaces including the surface roughness of the component. Then based on the deviation map with predefined tolerances limits, a report is generated to decide if the component is rejected or accepted. It is important to point out that at this point of the process the product does not have its final dimensions. Therefore, the process that follows this quality control gate, uses a CNC 5+1 axis milling machine (Fig 6b) to take the product to its final dimensions based on the results of this quality control gate, and where holes for future threads are further drilled to match the required tolerances [65].

The reason behind using both technologies, CMM and a 3D laser scanner, is to combine their advantages and counteract their disadvantages. Contact CMM provides very accurate high-resolution data used to detect defects of defined geometries with high accuracy [66]. For example, the ALTERA^{S} contact CMM used by Company A can detect defects of defined geometries with a high volumetric accuracy up to 1.8 µm [67]. However, contact CMM systems require long inspection times, and the data obtained is sparse making the inspection of freeform shapes a complex task. [68]. On the other hand, 3D laser scanners are non-contact measurement systems capable of simultaneously measuring thousands of points per second making them ideal for freeform shapes [68]. For example, Company A uses a Nikon LC15Dx laser scanner mounted on the ALTERA^{S} CMM for high precision parts (Fig 6a). This 3D laser scanner can take 70,000 points/s and intricate details of reflective materials with a proving error of 1.9 µm without the need of toxic spray treatments [69]. Despite 3D laser provide a dense point cloud the downside is that the resulting point cloud

materials
MDPI

frequently have noise data that requires further processing methods such as mesh refinement [70]. Therefore, by simultaneously using both technologies it is possible to obtain and accurate virtual representation of the real implant to perform detailed and fast metrological checks compared with micro-CT scanners.

a) b)

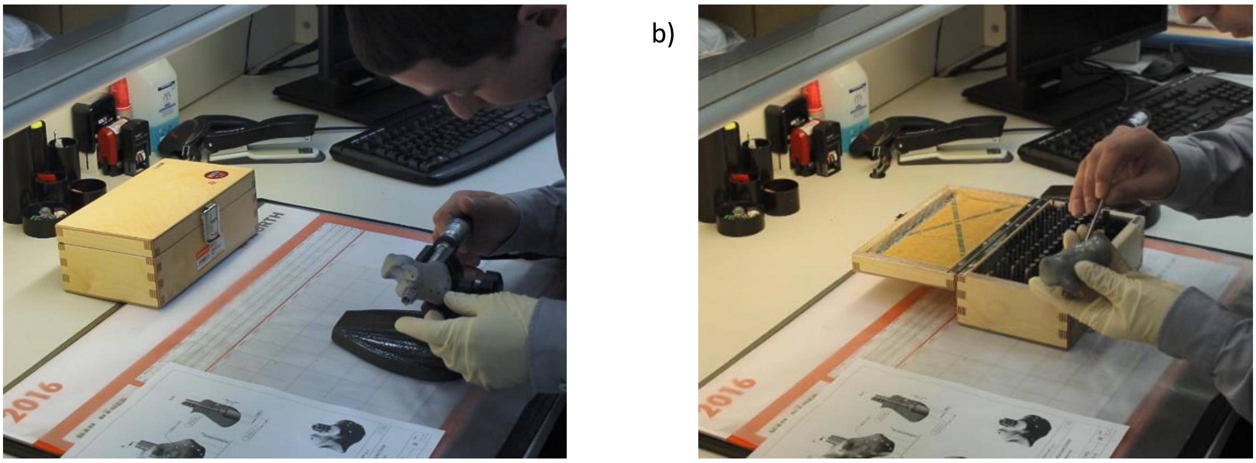

Figure 6. (a) and (b), show a visual inspection of a semi-finished patient-specific implant performed with traditional measurement tools.

a) b)

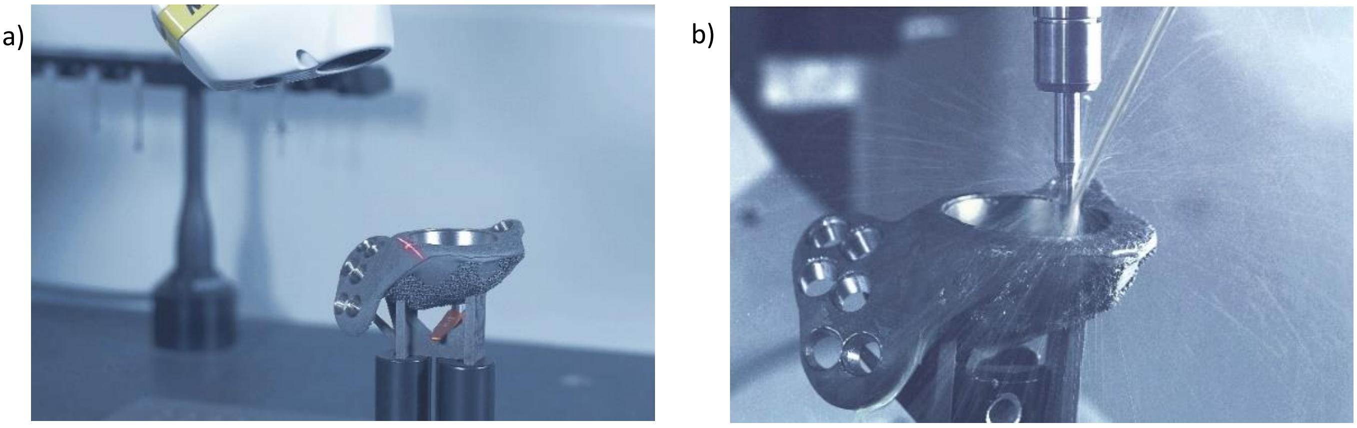

Figure 5. a) Nikon LC15Dx laser scanner for metrology inspection of semi-finished patient-specific implants; b) CNC 5+1 axis milling machine to taking a patient-specific acetabular cup to its final dimensions.

G-12: Periodic inspection of material microstructure and chemical composition

G-12 is a metallographic periodic inspection that takes place in order to certify that each manufactured batch complies with the required chemical composition and microstructure standards for its specific use. As metallographic examinations are considered destructive tests, this quality control gate uses test coupons that are built within each batch. The location and orientation of these test coupons should correspond to the worst-case scenarios previously identified in G-8. The chemical analysis for titanium alloys may be performed by X-Ray fluorescence (XRF) spectrometry following the ASTM standard E539 [71]. Additionally, according to ASTM standard F2924, the chemical composition of titanium Ti-6Al-4V alloy final products produced with AM Powder Bed Fusion made should be within the required limits, previously presented in Tables 2 and 3. Moreover, according to the ASTM standard F2924 [24] “the alpha case is not permitted on a metallurgical cross section at 100X magnification”. Therefore, Ti–6Al–4V alloy components have to be descaled and cleaned following the STM guide B600 [24].

It is known that when titanium and titanium alloys are heat treated above 480°C, they easily form a hard and brittle oxygen contaminated layer known as alpha case. This alpha case has detrimental effects on titanium mechanical properties such tensile ductility and fatigue resistance [72]. Moreover, the rapid heating and cooling cycles produced during the additive manufacturing process of powder bed fusion systems can affect the microstructure of metals. At the microscopic scale, metal microstructures can be seen as grains that can vary in shape and size and distributions

according to the crystal structure of the material. These microscopic features can be qualified, measured, and compared to determine if the sample satisfies a predetermine criteria [73]. Microstructures have a strong influence on material mechanical and chemical properties [74]. In the case of titanium alloys, they are classified in four main groups depending on their crystal structure at room temperature. These groups are α phase alloys (HCP crystal structure), near α alloys, α+ β alloys, and β phase alloys (BCC crystal structure) [75]. Titanium Ti–6Al–4V alloy crystal structure is α+ β. Thanks to the combination of these two phases this alloy has high strength, low modulus of elasticity, and high corrosion resistance [76]. Making this alloy ideal for cardiovascular and bone implant applications [77,78].

Metallographic examinations are performed using a light optical microscope or a scanning electron microscope [79]. According to the ASTM standards F1472 [80] and F2924 [24] the microstructure of final net shape components made of Ti–6Al–4V alloy "shall essentially consist of an equiaxed and/or elongated primary alpha in a transformed beta matrix with no continuous network of alpha at prior beta grain boundaries" [80]. The required standards to prepare metallographic specimens and to examine the microstructure of metals and alloys such as Ti-6Al-4V are the ASTM standards E3 and E407, respectively. The ASTM E3 standard describes how specimens should be selected and prepared to minimise alteration of metal microstructure in order to provide adequate data to reveal the maximum variations within the produced batch [79]. On the other hand, the ASTM E407 is a standard method for etching a metal sample to reveal its microstructure. This standard provides the necessary information such as chemical solutions and procedures for each metal and alloys for microscopic examination [81].

The results of the metallographic examinations should be reported in the device master record with microphotographs of the material microstructure along with a paragraph containing an interpretation of the results [79]. The test results of this quality control gate are used as a criterion for acceptance or rejection of an entire batch. If a batch is rejected appropriate heat treatments can help to adjust the material microstructure and in some cases material chemistry to the required standards.

a) b)

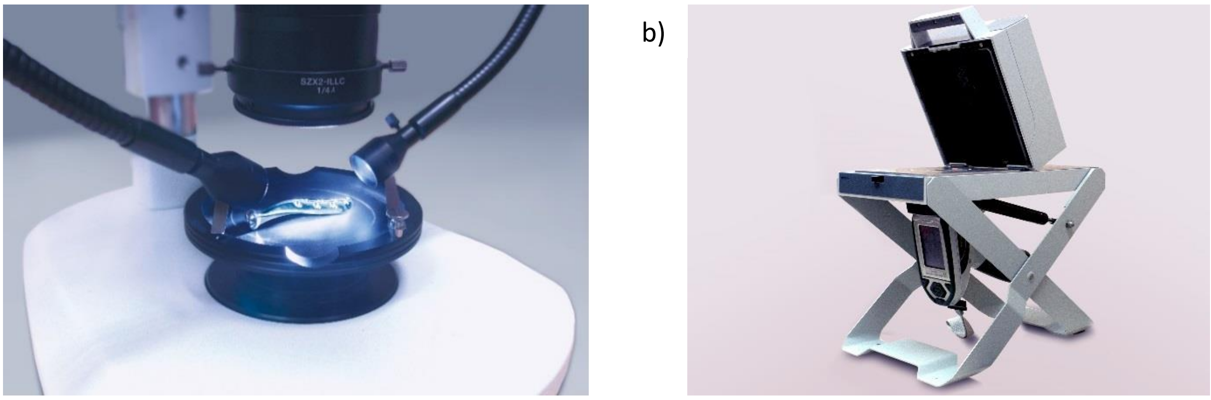

Figure 7. a) Metallographic examination with light optical microscope of a patient-specific component; b) X-Ray fluorescence (XRF) spectrometer.

G-13: Defectoscopy and dimensional analysis

G-13 uses a micro-CT scanner to control three different product critical quality attributes with a non-destructive test. The first attributes to control are the dimensional deviations of the implant final shape in relation to the virtual original design. As explained before some dimensional variations are introduced during the fabrication process and from the detachment of support structures and build platform. However, according to the AM engineers from the three studied companies post processes such sandblasting, and polishing removes a great amount of material from the implant surface reaching up to 200 µm or even more for manual processes.

The second product critical quality attribute to control is material integrity. This is due to the fact errors during the fabrication process with Powder Bed Fusion systems can introduce internal and external porosities in the material. Some of these internal porosities are caused by unfused particles due to defects on the coater blade [54], and wrong machine settings such as laser focus and power [40]. The third attribute to control is the implant's biological safety, which can be affected by the quantity of trapped particles in trabecular and lattice structures. Inclusion of particles in patient-specific implants is caused during the fabrication, and post processing processes such as sandblasting and polishing [82,83], as shown in Fig 8. During this study it was identified that all the companies studied developed their own confidential technologies to extract these particles from the implant trabecular structures. According to the AM

materials

Engineers and Quality Managers of the studied companies, a 100 percent extraction of particles is not possible yet. Nevertheless, Company B is able to extract powder particles up to 60% over the regulatory allowance with their own in-house developed system.

Micro-CT scanners are excellent tools that allows to perform defectoscopy and dimensional analysis in a single test [84]. Micro-CT scanners can detect internal cracks, inclusion of particles, and porosity in materials in just a few in seconds, making them suitable for in-line inspections [85]. Moreover, this is the only non-destructive technology available that can measure inner and outer geometries of complex parts with microns of accuracy [85]. This technology is also capable of taking high accuracy images of nano-size cross sections giving 10,000 times more resolution than conventional CT scanners and at a lower level of radiation exposure [86], being ideal for high precision implants [87].

By using this technology Company A can control dimensional variations up to 7 µm. This is done by creating a digital 3D representation of the real implant in the same way as a 3D volumetric reconstruction process, as presented in Fig 9c. Then in the same way as in the G-11, the original implant design is aligned with the 3D volumetric reconstruction of the real implant to identify dimensional deviations with a colour deviation map that allows the comparison of actual internal and external geometries with nominal values. Moreover, it is possible to look through the entire part to check the implant material integrity to identify internal pores, and powder particles trapped within the trabecular and lattice structures. The system that this company uses is a Nikon Metrology's XT H 225 system which includes a Varian 4030 digital panel and Inspect-X software, capable of penetrating dense materials such titanium alloys, and can accommodate large parts up to 50 cm and 15 kg, as shown in Fig 8 [88]. Moreover, this system can target spots as small as 3 µm with micro focus x-ray source that provides a high-resolution 3D image [69]. Table 5 shows a variety of standards related to non-destructive evaluation suitable for AM.

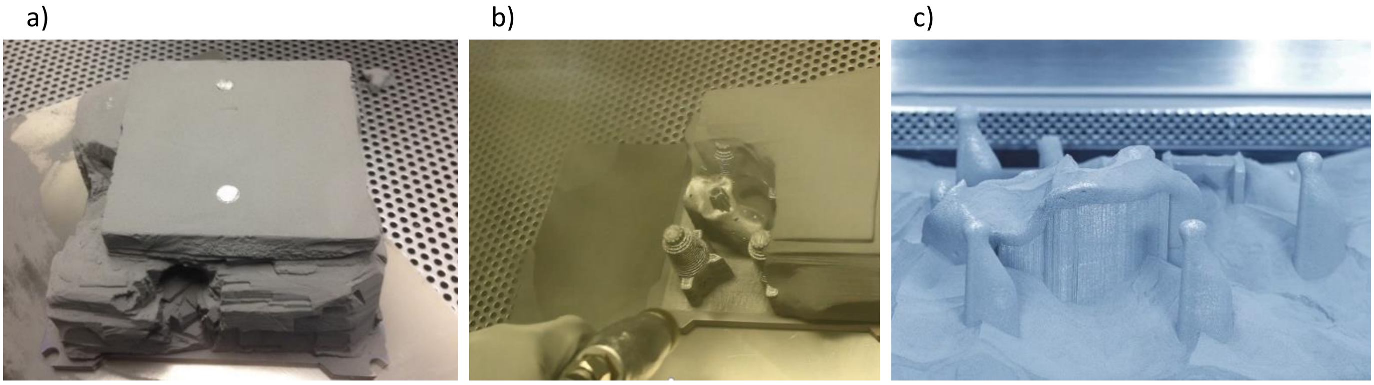

Figure 8. a) Powder cake containing EBM manufactured patient-specific implants; c) Blasting of the powder revealing manufactured components; c) Components manufactured with the DMLS system.

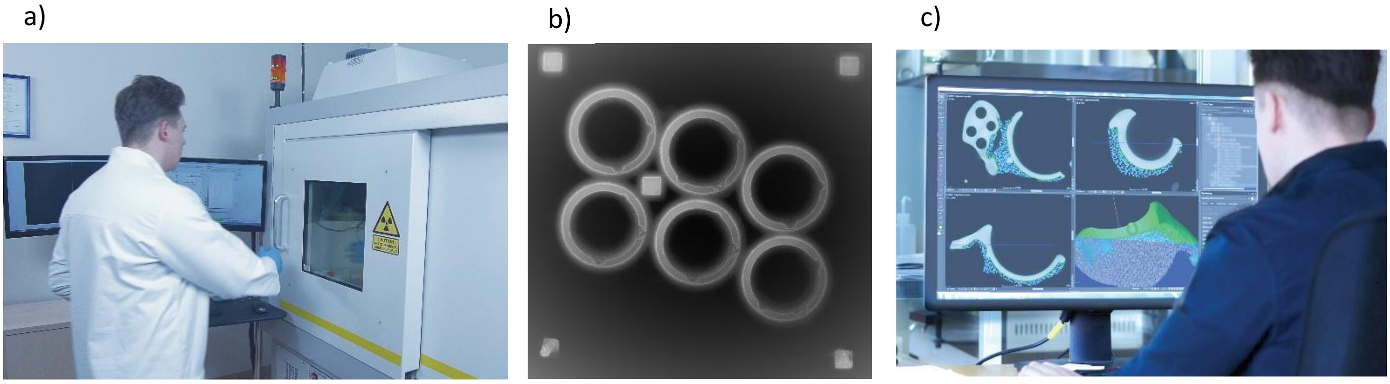

Figure 9. a) Micro CT-scanner Nikon XT H 225; b) Example of an X-ray scan of acetabular cups; c) Volume Graphics VGStudio MAX 3 software.

Table 5. Example of ASTM for non-destructive examinations with CT and Micro CT scanners.

Standard designation code	Standard
ASTM E1316	Standard Terminology for Nondestructive Examinations
ASTM E1441	Standard Guide for Computed Tomography (CT) Imaging
ASTM E1570	Standard Practice for Computed Tomographic (CT) Examination
ASTM E1814	Standard Practice for Computed Tomographic (CT) Examination of Castings
ASTM E1695	Standard Test Method for Measurement of Computed Tomography (CT) System Performance
ASTM E2767	Standard Practice for Digital Imaging and Communication in Nondestructive Evaluation (DICONDE) for X-ray Computed Tomography (CT) Test Methods
ASTM F3259	Standard Guide for Micro-computed Tomography of Tissue Engineered Scaffolds
ASTM F2450	Standard Guide for Assessing Microstructure of Polymeric Scaffolds for Use in Tissue-Engineered Medical Products
ASTM E3147	Standard Practice for Evaluating DICONDE Interoperability of Nondestructive Testing and Inspection Systems
ASTM E1817	Standard Practice for Controlling Quality of Radiological Examination by Using Representative Quality Indicators (RQIs)
ASTM E3169	Standard Guide for Digital Imaging and Communication in Nondestructive Evaluation (DICONDE)
ASTM E2339	Standard Practice for Digital Imaging and Communication in Nondestructive Evaluation (DICONDE)

G-14: Periodic inspection of mechanical properties

The static mechanical properties of as built, milled, and heat-treated titanium Ti6Al4V alloy components fabricated with DMLS and EBM can fall over the required values of the ASTM standard F2924 [74,89,90]. However, their strong dependency on part orientation and location within the build volume is a major concern to the final quality of patient-specific implants. Therefore, to guarantee consistent mechanical properties, the objective of G-14 is to perform periodic tests of each manufactured batch. For this the FDA recommends the use of test coupons for tensile and micro-hardness tests [8]. The test coupons should be built within each batch, and their location and orientation within the build volume shall correspond to the worst-case scenarios previously identified in G-8, in a similar way as presented in Fig 10. Moreover, the mechanical properties presented in Table 3 should be used as reference for this gate.

a) b)

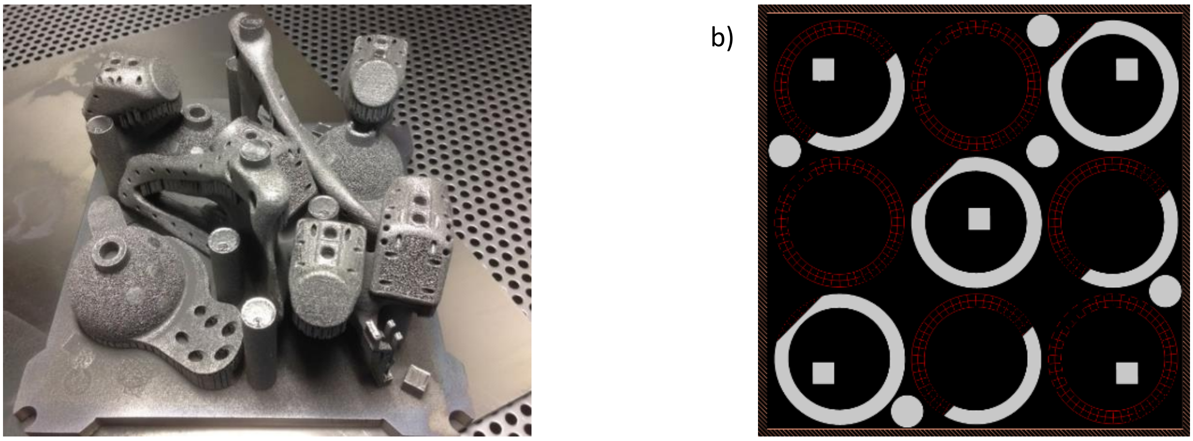

Figure 10. a) Test coupons located in worst-case scenarios between nested patient-specific implant components; b) Cross section of test coupons located in worst-case scenarios between acetabular cups.

G-15: Surface and coating characterization

Implant surface characteristics are critical for the evaluation of medical devices functionality, safety and quality. Many physical and chemical surface modification methods have been developed to improve biological, chemical, tribological, and mechanical properties of titanium implants [91], such as osseointegration, corrosion resistance, wear resistance, bioactivity, biocompatibility, and blood compatibility [92,93]. Some surface modification methods are aimed to change surface characteristics at the microscale, while others act at nanometre level or a combination of both [76,93,94]. However, different sections and components of patient-specific implants require distinct surface characteristics. For example, the implant areas that are in direct contact with bone is preferred a high roughness to improve cell attachment [95], whereas smooth polished surfaces are required in load bearing surfaces to reduce friction and wear [76]. To achieve high surface area in some patient-specific implants, lattice and trabecular structures are built during the additive manufacturing process. In the case of smooth surfaces, a high gloss finish with surface roughness of Ra < 0.2 μm are achieve through a series of fine grinding and polishing processes to create a perfect surface finish. Moreover, even though many titanium alloys are biocompatible, they must be treated to fulfil all the clinical requisites [76].

In general surface roughness measurement methods are classified in contact and non-contact. The most common contact method is stylus profilometry as the most surface finish standards are written for these profilometers [96]. Nevertheless, the disadvantages of the stylus profilometry are that is destructive surface measurement method that creates scratches and surface damage, and cannot be used for topography description of nanostructured surfaces already present in some commercial implants [97]. On the other hand, non-contact methods use areal topography measurement for characterization of surface topography with 2D and 3D parameters proving a 3D topographical map of the surface with more detailed information [98].

Due to the importance of controlling micro and nano surface characteristics in patient-specific implants, the aim of G-15 is to verify that the different implant surfaces follow the required specifications. However, surface roughness values are dependent on the scale of measurement [99]. Therefore, micrometric and nanometric features should be characterized separately [98]. In industry the most used standards for topography characterization are the ISO 25178 and the ISO 4287. ISO 25178 is a group of 17 documents and guidelines that describe and define areal parameters for different non-contact methods, whereas ISO 4287 provides general terms, definitions and surface texture parameters [100]. However, the ISO 4287 standard will be replaced by the ISO/NP 21920-2 which is under development. Examples of different standards for surface topography characterization are shown in Table 6.

Table 6. Example of some ASME, ASTM and ISO standards for surface topography characterization.

Standard designation code	Standard
ASME B46.1	Surface Texture (Surface Roughness, Waviness, and Lay)
ASTM F2791	Standard Guide for Assessment of Surface Texture of Non-Porous Biomaterials in Two Dimensions
ISO 4287	Geometrical Product Specifications (GPS) -- Surface texture: Profile method -- Terms, definitions and surface texture parameters
ISO 4288	Geometrical Product Specifications (GPS) -- Surface texture: Profile method -- Rules and procedures for the assessment of surface texture
ISO 3274	Geometrical Product Specifications (GPS) -- Surface texture: Profile method -- Nominal characteristics of contact (stylus) instruments
ISO 25178-1:2016	Geometrical product specifications (GPS) -- Surface texture: Areal -- Part 1: Indication of surface texture
ISO25178-2	Geometrical product specifications (GPS) -- Surface texture: Areal -- Part 2: Terms, definitions and surface texture parameters
ISO 25178-3	Geometrical product specifications (GPS) -- Surface texture: Areal -- Part 3: Specification operators
ISO 25178-6:2010	Geometrical product specifications (GPS) -- Surface texture: Areal -- Part 6: Classification of methods for measuring surface texture
ISO 25178-600	Geometrical product specifications (GPS) -- Surface texture: Areal -- Part 600: Metrological characteristics for areal topography measuring methods
ISO 25178-601	Geometrical product specifications (GPS) -- Surface texture: Areal -- Part 601: Nominal characteristics of contact (stylus) instruments

ISO 25178-602	Geometrical product specifications (GPS) -- Surface texture: Areal -- Part 602: Nominal characteristics of non-contact (confocal chromatic probe) instruments
ISO 25178-603	Geometrical product specifications (GPS) -- Surface texture: Areal -- Part 603: Nominal characteristics of non-contact (phase-shifting interferometric microscopy) instruments
ISO 25178-604:2013	Geometrical product specifications (GPS) -- Surface texture: Areal -- Part 604: Nominal characteristics of non-contact (coherence scanning interferometry) instruments
ISO 25178-605	Geometrical product specifications (GPS) -- Surface texture: Areal -- Part 605: Nominal characteristics of non-contact (point autofocus probe) instruments
ISO 25178-606	Geometrical product specification (GPS) -- Surface texture: Areal -- Part 606: Nominal characteristics of non-contact (focus variation) instruments
ISO 25178-607	Geometrical product specifications (GPS) -- Surface texture: Areal -- Part 607: Nominal characteristics of non-contact (confocal microscopy) instruments

a) b)

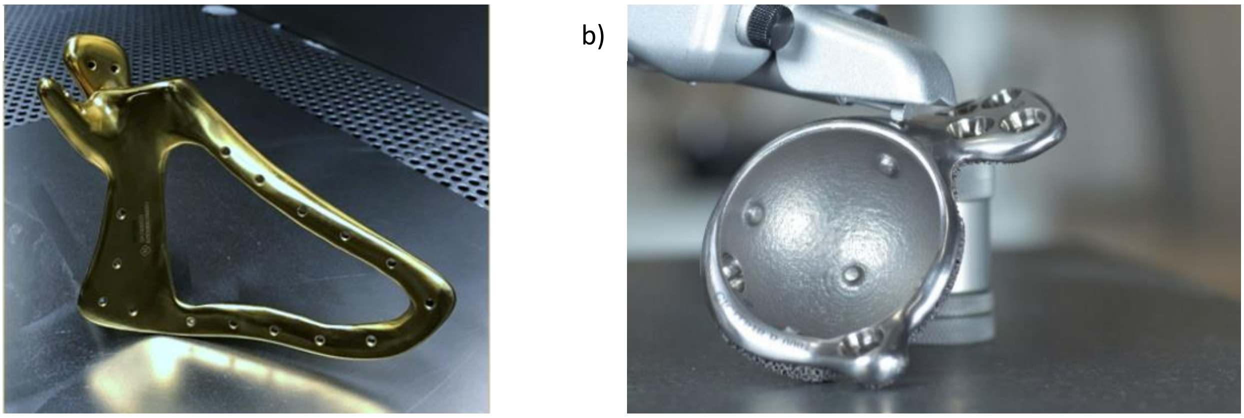

Figure 11. a) Scapula implant coated with titanium nitride (TiN) to reduce wear and minimise ion release; b) Surface roughness test with stylus profilometer on a non-coated patient-specific acetabular cup.

G-16: Detailed periodic/random inspection of finished product

Until this point of the production chain, the initial powder material has been transformed into a complex product through several processes to reach the final desired characteristics. In each of these processes different types of defects could have been imperceptibly propagated affecting the product overall mechanical properties. Most patient-specific joint implants are composed of standard and customised components that require to be assembled. To ensure the quality of standard components and standard implants manufactured with traditional methods, it is a common practice in industry to use destructive tests. These testing methods are based on random lot samples to statistically control the overall mechanical properties of a whole batch. This type of quality control method is very effective for mass production of standardised components.

In the medical industry it is believed that performing destructive testing of customised components is not feasible due to the time and costs involved. However, understanding defect propagation in multistage manufacturing processes combined with AM is a complex task [2], especially if they produce small batches of customised products that benefit very little from statistical quality control techniques. Furthermore, some mechanical properties of AM metal parts may differ with those fabricated with wrought metals. For example, according numerous studies the high surface roughness used in patient-specific implants to improve bone healing [101], negatively affects properties such as fatigue limit and crack initiation [90]. The as built surface roughness of titanium AM parts has such impact on the mechanical properties that even hot isostatic pressing (HIP) has little effect in improving fatigue life [74,90]; but in the absence of material defects, microstructure is the factor that dominates the fatigue performance and static mechanical properties [74,89].

Table 7. Example of different standards mechanical tests for various types of implants.

Standard designation code	Standard
ASTM F2033	Standard Specification for Total Hip Joint Prosthesis and Hip Endoprosthesis Bearing Surfaces Made of Metallic, Ceramic, and Polymeric Materials

ASTM F382	Standard Specification and Test Method for Metallic Bone Plates
ASTM F1800	Standard Practice for Cyclic Fatigue Testing of Metal Tibial Tray Components of Total Knee Joint Replacements
ASTM F1801	Standard Practice for Corrosion Fatigue Testing of Metallic Implant Materials
ASTM F1814	Standard Guide for Evaluating Modular Hip and Knee Joint Components
ASTM F2267	Standard Test Method for Measuring Load Induced Subsidence of Intervertebral Body Fusion Device Under Static Axial Compression
ASTM F3122	Guide for Evaluating Mechanical Properties of Metal Materials Made via AM Processes
ASTM F3140	Standard Test Method for Cyclic Fatigue Testing of Metal Tibial Tray Components of Unicondylar Knee Joint Replacements
ASTM F3187	Guide for Directed Energy Deposition of Metals
ISO 7206-10	Implants for surgery -- Partial and total hip-joint prostheses -- Part 10: Determination of resistance to static load of modular femoral heads
STP1025	Factors That Affect the Precision of Mechanical Tests
ASTM F2346	Standard Test Methods for Static and Dynamic Characterization of Spinal Artificial Discs

Table 8. Example of standard tests for different types of implant surfaces and coatings.

**Standard designation code**	**Standard**
ASTM G171	Standard Test Method for Scratch Hardness of Materials Using a Diamond Stylus
ASTM C 633	Standard Test Method for Adhesion or Cohesion Strength of Thermal Spray Coatings
ASTM C1624	Standard Test Method for Adhesion Strength and Mechanical Failure Modes of Ceramic Coatings by Quantitative Single Point Scratch testing
ASTM F86	Standard Practice for Surface Preparation and Marking of Metallic Surgical Implants
ASTM F1044	Standard Test Method for Shear Testing of Calcium Phosphate Coatings and Metallic Coatings
ASTM F1147	Standard Test Method for Tension Testing of Calcium Phosphate and Metallic Coatings
ASTM F1377	Standard Specification for Cobalt28Chromium6Molybdenum powder for Coating of Orthopedic Implants
ISO 14243-2	Implants for surgery — Wear of total knee-joint prostheses — Part 2: Methods of measurement
ISO 14602	Non-active surgical implants — Implants for osteosynthesis — Particular requirements
ISO 14577	Metallic materials — Instrumented indentation test for hardness and materials parameters — Part 4: Test method for metallic and non-metallic coatings
ASTM F1978	Standard Test Method for Measuring Abrasion Resistance of Metallic Thermal Spray Coatings by Using the Taber Abraser
ASTM F2033 -	Standard Specification for Total Hip Joint Prosthesis and Hip Endoprosthesis Bearing Surfaces Made of Metallic, Ceramic, and Polymeric Materials
JIS H 8666	Test methods for ceramic sprayed coatings
ISO/DIS 17327-1	Non-active surgical implants — Implant coating — Part 1: General requirement

In the case of modified and non-modified surfaces of metallic implants there are several surface characteristics that need to be controlled, such as hardness, layer thickness, shear fatigue strength, static shear strength, plastic deformation, and abrasion [102]. ISO 17327 standard provide a framework for the design and test of coatings for non-active surgical implants [103]. For example, according to the FDA *Guidance Document for Testing Orthopedic Implants with Modified Metallic Surfaces* [102] the static shear and tensile strength of the surface/substrate interface must exceed 20 MPa, and should be tested following the ASTM F 1044 and ASTM F 1147 respectively.

Taking all this into consideration, the aim of the G-16 is to perform a strict quality control of the final product that does not leave space for uncertainty, thus ensuring the long-term safety of patient-specific implants. For this purpose, this quality control gate is designed to test standard and bespoke components differently. The production of additively manufactured standard components has the advantage that they can be can easily be tested using statistical methods. Therefore, to guarantee tribological properties, and the static and dynamic mechanical properties each build batch of additively manufactured standard components, the objective of G-16 is to perform periodic random destructive tests. In the case of bespoke components, they can only be randomly tested if a strong data base is present. This data base should contain enough information about all the different variations of an implant family to be able to predict the

mechanical behaviour of its different variations. If this is not the case, bespoke components should be manufactured with a twin coupon to be subjected to the same destructive tests of AM standard components. These tests should be performed not just to control quality, but also to create a strong data base for continuous improvement of the whole manufacturing process chain. In Tables 7 and 8 are listed various ASTM and ISO standards that should be considered for different types of bone implants and bone implant coatings.

G-17: Visual inspection of finished products

G-17 is a comprehensive off-line visual inspection of the final product. The aim of this quality control gate is to detect residual errors that could not be detected in previous stages. Here an inspector checks the overall quality of each implant and assembly, including all the product documentation from the previous quality control gates. In this quality control gate, an expert visually compares each component and assembly with the original design and its specified tolerances using basic measurement tools such as calliper and micrometre, as shown in Fig 12. This includes active attempts to test the relative movement between components. Some of the critical areas to be measured are holes, threads, assembly tolerances and movement, and the height and width of each component using predetermined reference points. Product quality deviations in this process are compared with pre-specified tolerances and categorised according to its criticality based on a ranking criterion. Then depending on the score obtained it is decided if the component is rejected or accepted.

According to Fox [104] there are four important stages that need to be followed during visual inspections. These stages are visual screening; finding a defect ("detection"); defect classification; decision that classifies a component. During visual screening the search for potential defects takes place. The effectiveness of human visual inspection can vary from 52% to 100% and depends on task complexity and training [12]. Room illumination can drastically affect inspectors' visual performance. Therefore, for difficult and highly difficult inspection work 1000, and 2000 lux are recommended [105]. Visual screening strategy is also an important factor to take into account. According to See [106] systematic search is the most precise and efficient strategy. However, this type of search strategy requires perfect memory of previous scanned areas [106]. Therefore, to efficiently and accurately finding defects inspectors may use visual aids such as engineering drawings, including documents and standards that describe the type of errors that can be encountered [107]. Furthermore, previous knowledge about the most common locations of errors eliminates the need to rely on long-term memory, and increases inspectors success rates [12]. In regard to the decision of acceptance or rejection of components, it is important to highlight that this type of quality control activity is highly sensible due to its subjective nature and dependency on expert knowledge and experience [63]. Therefore, adequate training of cognitive abilities, procedures and the use of tools is vital [12]. If a component or assembly are accepted, then they are labelled to later be cleaned, sterilised and packed. Otherwise, depending on the type of quality issue they are send for rework or discarded. Nevertheless, discarded components are classified and stored. The purpose of this is that defective products can help to better understand the cause and roots of errors in order prevent similar mistakes to improve performance of the whole production chain.

a) b) c)

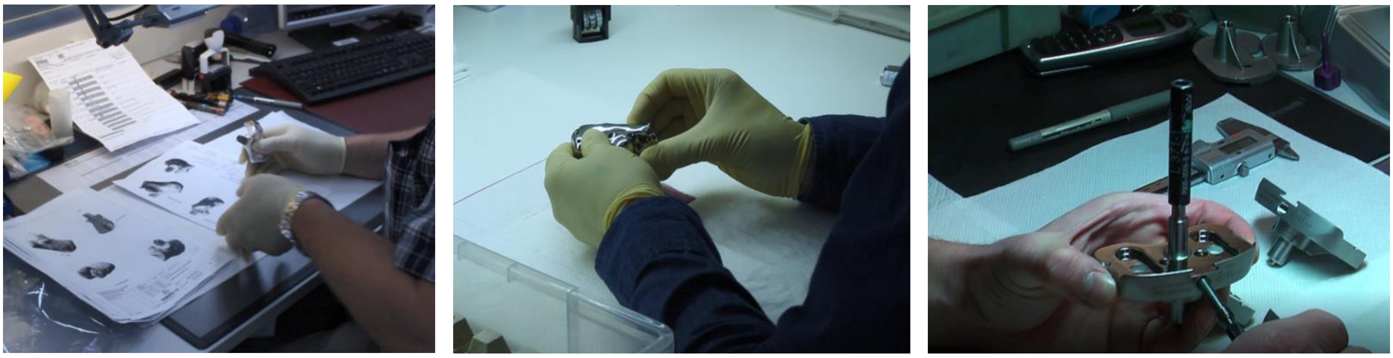

Figure 12. (a) and (b) show a detailed visual inspection of a finished patient-specific implant; (c) visual inspection of a standard tibial component

G-18: Sterilization and packaging validation

Cleaning, disinfection, and sterilization, marking, labelling, and packaging of critical medical devices such as patient-specific implants are vital activities that if they are inadequately performed it can lead to adverse effects to patients such as infections and mortality/morbidity result in significant institutional costs [108]. Identification of potential

contaminants that can influence the safety of patient-specific implants can represent a challenge for many manufacturers [109]. This is due to the fact that the evaluation and validation of cleaning, disinfection, and sterilization methods require an exhaustive knowledge of the manufacturing process, handling, and packaging [110]. Moreover, due to the geometrical complexity of patient-specific implants this type of medical products may undergo several cleaning and sterilization steps in order to guarantee their safety and biocompatibility [109]. However, cleaning, disinfection and sterilization of additively manufactured devices is not different than other medical devices within the same classification [83].

Medical devices should be thoroughly cleaned prior to disinfection and sterilization in order to remove micro-organisms hidden behind proteinaceous or greasy matter [111]. The goal of cleaning medical devices is to remove organic and inorganic particles and molecules coming from the previous manufacturing steps. Disinfection and sterilization are both decontamination processes. While disinfection is the process of eliminating or reducing harmful microorganisms from inanimate objects and surfaces, sterilization is the process of killing and stopping the reproduction of microorganisms such virus, fungi, bacteria and spores [111,112]. There are different cleaning, disinfection, and sterilization methods available and their selection mainly depends on the implant's material properties [113,114]. However, there are no cleaning, disinfection, and sterilization processes for all different types of medical devices and materials [115]. Therefore, the efficiency and safety of the selected cleaning, disinfection, and sterilization processes should be considered when choosing a biomaterial, designing the device and choosing the packaging and sterilisation technique [111]. Moreover, in the case of titanium implants with modified surface it is critical to take into account the effects of cleaning and sterilization in surface properties [112]. It had been demonstrated that surface hydrophobicity and roughness are affected by these processes leading to the alteration of implant biological performance [112].

During our visit to the premises of Company B, we identified that this company selected high-power ultrasound, washer-disinfectors, and gamma radiation as their cleaning, disinfection, and sterilization processes for coated and non-coated titanium implants. In an industrial setup, multi chamber ultrasonic cleaning machines and high-power ultrasound machines (Fig 13a, and 13b) are used to apply high-frequency sound waves to remove a variety of organic and inorganic contaminants such as oils, chips and powder material from metallic implants immersed in aqueous media. When high-frequency sound waves are transmitted through in an aqueous media, these pressure waves create cavitation bubbles that implode freeing contaminants from their bonds with the implant substrate [116,117]. This cleaning process provides an excellent penetration and cleaning of small and intricate geometries such as the lattice/trabecular structures of patient-specific implants without damaging the substrate [118,119]. For ultrasonic cleaning the selection the appropriate chemical composition of the medium is an critical element no only to speed up the removal rate, but also to achieve a consistent cleaning process to meet production and quality demands [117].

a) b) c)

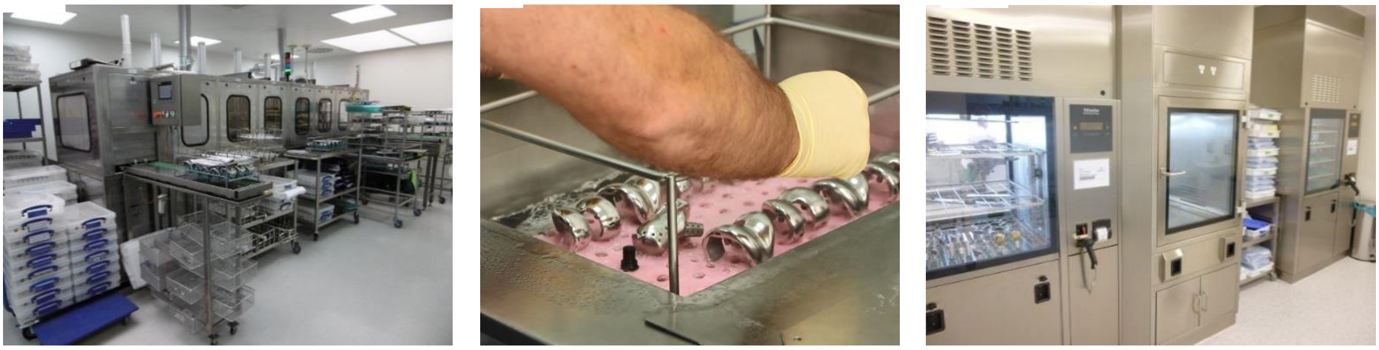

Figure 13. (a) Automatic cleaning thermic disinfection; (b) Multi chamber ultrasonic cleaning; and (C) Ultrasonic cleaning of a patient-specific implants and femoral component of standard knee implants.

In order to inactivate microorganisms except some heat resistant viruses, cryptosporidia, and bacterial spore's thermal washer disinfectors are used in the medical device industry [120]. Automatic washer-disinfectors combine cleaning and heat disinfection using demineralised water and moist, followed by a drying stage with saturated steam

(Fig 13c) [121]. Consequently, these machines are only suitable for medical devices that are not vulnerable to wet heat at temperatures of up to 80ºC [120]. Following the disinfection process, a gamma radiation sterilization process is performed to complete destruction of all viable micro-organisms including spores and viruses. Gamma radiation is a cost-effective industrial sterilization process for large batches of single-use items such as metallic implants [111,121]. The sterility is achieved by keeping medical devices in the vicinity of a radioactive source of gamma radiation such as Cobalt-60 isotope [114]. The emission of gamma rays ionizes key cellular components such as DNA bonds preventing cellular division and leading to death of microorganisms [114]. The advantage of Gamma radiation is that is a penetrating sterilant that can sterilize high-density products and its packaging with very little temperature effect without uncertain sterility after treatment [115]. The most commonly validated dose used to sterilize medical products is 25 kiloGrays. Nevertheless, gamma radiation has detrimental effects in polymers such as Polyethylene (PE) and Ultra High Molecular Weight PE. Some of the adverse effects of gamma rays in these materials are oxidation and decreased crystallinity leading to embrittlement of the polymer. To counteract these effects in polymers, gamma radiation is usually performed in an inert atmosphere or vacuum [111,114].

Some of the risks related with marking, labelling, and packaging are implantation of wrong device due to wrong marking and labelling, infection, and premature revision surgery caused by defective packaging [122,123]. Permanent marking of orthopaedic components is required for its traceability. Some of the risks related to wrong marking are implantation of wrong device and premature revision surgery [122]. Permanent marking in metallic components is usually performed with a laser (Fig 14a) to add information such as the manufacturer, material, lot number, model number, implant size, and whether an implant is intended for right limb or left limb reconstruction [124]. However, it is important to take into account that the location of laser marking can have detrimental effects in the fatigue life of load bearing implants [125]. Therefore, marking location and its effects on implant's mechanical properties should be considered during the implant design process and in G-4.

The objective of implant packaging is to provide an external barrier between the environment and the implant to ensure that the implant remains sterilized and intact until it reaches the operation room [123]. Package sealing and integrity are one of the main challenges in the implant industry [123]. Usually the implant designer is also responsible for the packaging design or selection of it. A well designed package system for orthopaedic implants is a factor that can provide to a company competitive advantage, thus details about packaging systems are well kept secrets by each company [123], Fig 14b presents an example of a packaging system for an orthopaedic implant. However, issues with packaging orthopaedic implants can also represent serious financial costs, such as several cases of well-known companies due to large scale recalls [126,127].

a) b)

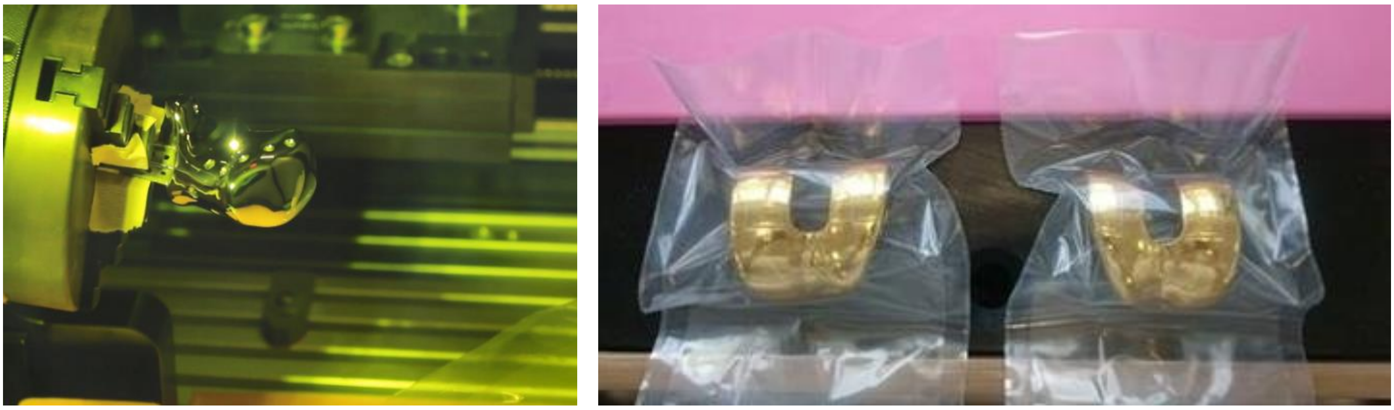

Figure 14. a) Laser marking of a patient-specific orthopaedic implant; b) Flexible packaging for knee primary endoprosthesis.

Considering the potential adverse effects caused by unwanted inorganic agents, and transmission of microorganisms such as bacteria, viruses and fungi during the fabrication, handling, and packaging of implants, the aim of the G-18 is to validate the performance and monitoring of each of different processes employed for disinfection, cleaning,

sterilization, marking, labelling, and packaging. Sterilization can also leave toxic residues or by-products formed during sterilization. Therefore, the validation of these processes shall be performed under actual manufacturing conditions and with the products in its final packaging configuration [111]. The sterility validation of medical devices at industrial scale can be performed using a small number of product samples to determine the sterility assurance level (SAL) using sterilization product development and validation studies [114]. The SAL is the probability that a product is not sterile after a specified sterilization process [128]. Usually the maximum acceptable SAL is 10^{-6} [128]. After validation, also the efficiency of disinfection, cleaning, and sterilization processes most be routinely monitored on each cycle. Delivery of the sterilization process to the sterilization load shall be verified by confirming, that within specified tolerances, data obtained during routine processing are the same as data obtained during validation. Production engineers must check sterilization certificates, sterilization indicators to verify the minimum and maximum dosages of gamma radiation received by the product. When several sterilization processes are used, product release can be based on dosimetric release, parametric control, or process control [111]. Once the sterilization of products is verified an expiration date is labelled on each on sterilised product, then a final visual inspection is performed to confirm that each component was adequately marked based on patient information and intended used. After, the final visual inspection products are released for shipment. It is important to consider that to get the European Community (EC) or FDA approval, the sterilization validation process requires functional and biocompatibility tests on the final packaged and sterilized products [108] which have to follow standards for these processes such as the ones presented in Table 9.

Regarding, the main implant package it is important to inspect it in an exhaustive way to identify potential issues such as punctures, damage, or defective sealing. For the external package labelling there are also standards that need to be followed, in order to clearly present important information of the implant. This information is aimed facilitate implant storage and identification. The ASTM F2943 standard guide proposes a unified universal format package labelling that includes the layout and location of information, to facilitate product identification in hospitals inventory and operating room environment [129]. For more information about related standards for marking, labelling, and packaging refer Table 10.

Table 9. Example of standards for cleaning, sterilization, and biological evaluation of orthopaedic implants.

Standard designation code	Standard
ANSI/AAMI ST79:2017	Comprehensive Guide to Steam Sterilization and Sterility Assurance in Health Care Facilities
ISO 19227:2018	Implants for surgery — Cleanliness of orthopaedic implants — General requirements
ISO 10993-1	Biological evaluation of medical devices -- Part 1: Evaluation and testing within a risk management process
ISO 10993-3:2014	Biological evaluation of medical devices -- Part 3: Tests for genotoxicity, carcinogenicity and reproductive toxicity
ISO 10993-4:2017	Biological evaluation of medical devices -- Part 4: Selection of tests for interactions with blood
ISO 10993-5:2009	Biological evaluation of medical devices -- Part 5: Tests for in vitro cytotoxicity
AAMI/ANSI/ISO 11137-1	Sterilization of health care products -- Radiation -- Part 1: Requirements for development, validation and routine control of a sterilization process for medical devices
AAMI/ANSI/ISO 11137-2	Sterilization of health care products -- Radiation -- Part 2: Establishing the sterilization dose
AAMI/ANSI/ISO 11137-3	Sterilization of health care products -- Radiation -- Part 3: Guidance on dosimetric aspects of development, validation and routine control
ISO 11134	Sterilization of health care products -- Requirements for validation and routine control -- Industrial moist heat sterilization
ISO 11135:1994	Sterilization of health-care products -- Ethylene oxide -- Requirements for the development, validation and routine control of a sterilization process for medical devices
ISO 17664	Sterilization of medical devices — Information to be provided by the manufacturer for the processing of resterilizable medical devices
ISO 15883-1:2006	Washer-disinfectors -- Part 1: General requirements, terms and definitions and tests
ISO 15883-2:2006	Washer-disinfectors -- Part 2: Requirements and tests for washer-disinfectors employing thermal disinfection for surgical instruments, anaesthetic equipment, bowls, dishes, receivers, utensils, glassware, etc.
ISO/CD 15883-5	Washer disinfectors -- Part 5: Performance requirements and test method criteria for demonstrating cleaning efficacy

Table 10. Example of ASTM and ISO standards for marking, labelling, and packaging for orthopaedic implants.

Standard designation code	Standard
ASTM F86	Standard Practice for Surface Preparation and Marking of Metallic Surgical Implants
ASTM F983	Standard Practice for Permanent Marking of Orthopaedic Implant Components
ASTM F2943 - 14	Standard Guide for Presentation of End User Labelling Information for Musculoskeletal Implants
ASTM F1886 / F1886M	Standard Test Method for Determining Integrity of Seals for Flexible Packaging by Visual Inspection
ISO 11607	Packaging for Terminally Sterilized Medical Devices -- Part 1: Requirements for Materials, Sterile Barrier Systems and Packaging Systems

Revalidation

According to the FDA any changes that are introduced at any point or the manufacturing process and products will lead to the need for revalidation [8]. Revalidation is required due to the potential risks that can emerge in the workflow process such as: the medical device design; software update, machine settings; manufacturing location; manufacturing process [8]. For example, when software is updated some time unidentified software bugs or defects can be introduced [14]. Moreover, as best practice some of the participant companies of this study perform and annual assessment of validation to keep their processes calibrated and in check.

References

1. Shi, J.; Zhou, S. Quality control and improvement for multistage systems: A survey. *IIE Transactions* **2009**, *41*, 744-753.
2. Colledani, M.; Tolio, T.; Fischer, A.; Iung, B.; Lanza, G.; Schmitt, R.; Váncza, J. Design and management of manufacturing systems for production quality. *CIRP Annals-Manufacturing Technology* **2014**, *63*, 773-796.
3. Hrgarek, N.; Bowers, K.-A. Integrating six sigma into a quality management system in the medical device industry. *Journal of Information and Organizational Sciences* **2009**, *33*, 1-12.
4. Chang, D. Internalizing the External Costs of Medical Device Preemption. *Hastings LJ* **2013**, *65*, 283.
5. He, D. Engineering Quality Systems: Cost of Quality. *Modern Applied Science* **2010**, *Vol. 4*, 102-104.
6. Nicholas, J.M.; Steyn, H. *Project management for business, engineering, and technology: principles and practice*; Elsevier: New York, USA, London, UK, 2008.
7. Martinez-Marquez, D.; Mirnajafizadeh, A.; Carty, C.P.; Stewart, R.A. Application of quality by design for 3D printed bone prostheses and scaffolds. *PLOS ONE* **2018**, *13*, e0195291, doi:10.1371/journal.pone.0195291.
8. FDA. Technical Considerations for Additive Manufactured Medical Devices: Guidance for Industry and Food and Drug Administration Staff. Services, U.S.D.o.H.a.H., Ed. U.S Food & Drug Administration: 2017.
9. Appleton, E. Product Design for Manufacture and Assembly. *Assembly Automation* **2008**, *28*, doi:10.1108/aa.2008.03328cae.001.
10. Craft, R.C.; Leake, C. The Pareto principle in organizational decision making. *Management Decision* **2002**, *40*, 729-733, doi:10.1108/00251740210437699.
11. Junior, O.C.; Okumura, M.L.M.; Young, R.I.M. The application of an integrated product development process to the design of medical equipment. 2015; 10.1007/978-3-319-13776-6_25.
12. Kujawińska, A.; Vogt, K. Human factors in visual quality control. *Management and Production Engineering Review* **2015**, *6*, 25-31.
13. Bhat, K.S.; ebrary, I. *Total quality management: text and cases*; Himalaya Pub. House: Mumbai [India], 2010; Vol. Rev.
14. FDA. General Principles of Software Validation; Final Guidance for Industry and FDA Staff. Services, H.a.H., Ed. FDA: Rockville, Maryland, USA, 2002.
15. ISO/ASTM. Standard Specification for Additive Manufacturing File Format (AMF) Version 1.21. ASTM International: West Conshohocken, PA, USA, 2016.

16. Bordat, C.; McCullouch, B.; Sinha, K. *An analysis of cost overruns and time delays of INDOT projects*; Joint Transportation Research Program, Indiana Department of Transportation and Purdue University: West Lafayette, Indiana, 2004; p 11.
17. Clemens, N. *The New European Medical Device Regulation 2017/745: Main Changes and Challenges*; The association of clinical research professionals: Washington, D.C., USA, 2017; 10.14524/CR-17-0028pp. 26-29.
18. Qian, M.; Xu, W.; Brandt, M.; Tang, H. Additive manufacturing and postprocessing of Ti-6Al-4V for superior mechanical properties. *MRS Bulletin* **2016**, *41*, 775-784.
19. Caiazzo, F.; Alfieri, V. Simulation of Laser-assisted Directed Energy Deposition of Aluminum Powder: Prediction of Geometry and Temperature Evolution. *Materials* **2019**, *12*, 2100.
20. Han, J.; Wu, M.; Ge, Y. A Study on the Dimension Accuracy on the Inner Structure of the 3D Printed Parts Caused by the Scanning Strategy. *Materials* **2019**, *12*, 1333.
21. ASTM. Standard Test Method for Finite Element Analysis (FEA) of Metallic Orthopaedic Total Knee Femoral Components under Closing Conditions. ASTM: West Conshohocken, PA, USA, 2016; Vol. F3161 - 16.
22. ASTM. Standard Practice for Finite Element Analysis (FEA) of Non-Modular Metallic Orthopaedic Hip Femoral Stems. ASTM International: West Conshohocken, PA, USA, 2013; Vol. F2996 - 13.
23. ASTM. ASTM WK59162 New Test Method for Finite Element Analysis (FEA) of Metallic Orthopaedic Total Knee Tibial Components. Availabe online: https://www.astm.org/DATABASE.CART/WORKITEMS/WK59162.htm (accessed on March 19th, 2019).
24. ASTM. Standard Specification for Additive Manufacturing Titanium-6 Aluminum-4 Vanadium with Powder Bed Fusion1. ASTM international: West Conshohocken, PA, USA, 2014; Vol. F2924 – 14.
25. ASTM. Standard Guide for Characterizing Properties of Metal Powders Used for Additive Manufacturing Processes. ASTM international: West Conshohocken, PA, USA, 2014; Vol. F3049 – 14.
26. Dawes, J.; Bowerman, R.; Trepleton, R. Introduction to the additive manufacturing powder metallurgy supply chain. *Johnson Matthey Technology Review* **2015**, *59*, 243-256.
27. Sun, Y.; Aindow, M.; Hebert, R.J. Comparison of virgin Ti-6Al-4V powders for additive manufacturing. *Additive Manufacturing* **2018**, *21*, 544-555.
28. Sun, Y.; Aindow, M.; Hebert, R.J. The effect of recycling on the oxygen distribution in Ti-6Al-4V powder for additive manufacturing. *Materials at High Temperatures* **2018**, *35*, 217-224.
29. Hebert, R.J. metallurgical aspects of powder bed metal additive manufacturing. *Journal of materials science* **2016**, *51*, 1165-1175.
30. Zhang, Y.; Gupta, R.K.; Bernard, A. Two-dimensional placement optimization for multi-parts production in additive manufacturing. *Robotics and Computer Integrated Manufacturing* **2016**, *38*, 102-117, doi:10.1016/j.rcim.2015.11.003.
31. Hitzler, L.; Janousch, C.; Schanz, J.; Merkel, M.; Heine, B.; Mack, F.; Hall, W.; Öchsner, A. Direction and location dependency of selective laser melted AlSi10Mg specimens. *Journal of Materials Processing Technology* **2017**, *243*, 48-61.
32. Hitzler, L.; Merkel, M.; Hall, W.; Öchsner, A. A Review of Metal Fabricated with Laser-and Powder-Bed Based Additive Manufacturing Techniques: Process, Nomenclature, Materials, Achievable Properties, and its Utilization in the Medical Sector. *Advanced Engineering Materials* **2018**, *20*, 1700658, doi:10.1002/adem.201700658.
33. Galati, M.; Minetola, P.; Rizza, G. Surface Roughness Characterisation and Analysis of the Electron Beam Melting (EBM) Process. *Materials* **2019**, *12*, 2211.
34. Toeppel, T.; Schumann, P.; Ebert, M.-C.; Bokkes, T.; Funke, K.; Werner, M.; Zeulner, F.; Bechmann, F.; Herzog, F. 3D analysis in laser beam melting based on real-time process monitoring. In Proceedings of Mater Sci Technol Conf, Salt Lake City, UT, USA, 23–27 October, 2016.
35. Severson, K.; Chaiwatanodom, P.; Braatz, R.D. Perspectives on process monitoring of industrial systems. *Annual Reviews in Control* **2016**, *42*, 190-200, doi:10.1016/j.arcontrol.2016.09.001.
36. Boone, N.; Zhu, C.; Smith, C.; Todd, I.; Willmott, J.R. Thermal near infrared monitoring system for electron beam melting with emissivity tracking. *Additive Manufacturing* **2018**, *22*, 601-605, doi:10.1016/j.addma.2018.06.004.
37. Everton, S.K.; Hirsch, M.; Stravroulakis, P.; Leach, R.K.; Clare, A.T. Review of in-situ process monitoring and in-situ metrology for metal additive manufacturing. *Materials & Design* **2016**, *95*, 431-445.

38. Tapia, G.; Elwany, A. A review on process monitoring and control in metal-based additive manufacturing. *Journal of Manufacturing Science and Engineering* **2014**, *136*, 060801.
39. Butler, J. Using selective laser sintering for manufacturing. *Assembly Automation* **2011**, *31*, 212-219.
40. Gong, H.; Rafi, K.; Gu, H.; Ram, G.J.; Starr, T.; Stucker, B. Influence of defects on mechanical properties of Ti–6Al–4 V components produced by selective laser melting and electron beam melting. *Materials & Design* **2015**, *86*, 545-554.
41. Juechter, V.; Scharowsky, T.; Singer, R.; Körner, C. Processing window and evaporation phenomena for Ti–6Al–4V produced by selective electron beam melting. *Acta Materialia* **2014**, *76*, 252-258.
42. Bikas, H.; Stavropoulos, P.; Chryssolouris, G. Additive manufacturing methods and modelling approaches: a critical review. *The International Journal of Advanced Manufacturing Technology* **2015**, 1-17.
43. Murr, L.; Quinones, S.; Gaytan, S.; Lopez, M.; Rodela, A.; Martinez, E.; Hernandez, D.; Martinez, E.; Medina, F.; Wicker, R. Microstructure and mechanical behavior of Ti–6Al–4V produced by rapid-layer manufacturing, for biomedical applications. *Journal of the mechanical behavior of biomedical materials* **2009**, *2*, 20-32.
44. Gong, H.; Rafi, K.; Gu, H.; Starr, T.; Stucker, B. Analysis of defect generation in Ti–6Al–4V parts made using powder bed fusion additive manufacturing processes. *Additive Manufacturing* **2014**, *1-4*, 87-98, doi:10.1016/j.addma.2014.08.002.
45. Liu, H. Numerical analysis of thermal stress and deformation in multi-layer laser metal deposition process. Missouri University of Science and Technology, Rolla, Missouri, USA, 2014.
46. Li, S.J.; Murr, L.E.; Cheng, X.Y.; Zhang, Z.B.; Hao, Y.L.; Yang, R.; Medina, F.; Wicker, R.B. Compression fatigue behavior of Ti–6Al–4V mesh arrays fabricated by electron beam melting. *Acta Materialia* **2012**, *60*, 793-802, doi:10.1016/j.actamat.2011.10.051.
47. Arcam, A.B. Process Validation Tools. Availabe online: http://www.arcam.com/technology/electron-beam-melting/process-validation-tools/ (accessed on January 3rd, 2019).
48. Petelet, M. Additive Manufacturing: In-situ Process Monitoring, Defect Detection and Control. In Proceedings of Materials Science & Technology, Columbus, Ohio USA, 14–18 October 2018.
49. GE-additive. Electron Beam Melting (EBM) machines. Availabe online: https://www.ge.com/additive/additive-manufacturing/machines/ebm-machines/arcam-ebm-q10plus (accessed on January 3rd, 2019).
50. EOS. EOSTATE System: Control over all production-relevant data in the 3D printing process. Availabe online: https://www.eos.info/software/monitoring-software/eostate-system (accessed on January 4th, 2019).
51. EOS. EOS Software for Additive Manufacturing. Availabe online: https://www.eos.info/systems_solutions/software (accessed on January 4th, 2019).
52. Fuchs, L.; Eischer, C. In-process monitoring systems for metal additive manufacturing. EOS Electro Optical Systems: 2018.
53. EOS. EOSTATE PowderBed: Recording every coating and exposure phase. Availabe online: https://www.eos.info/software/monitoring-software/eostate-powderbed-control (accessed on January 4th).
54. Foster, B.; Reutzel, E.; Nassar, A.; Hall, B.; Brown, S.; Dickman, C. Optical, layerwise monitoring of powder bed fusion. In Proceedings of Solid Freeform Fabrication Symposium, Austin, TX, Aug, 10–12 August 2015; pp. 10-12.
55. EOS. Technical Description EOSINT M 280. Availabe online: https://webbuilder5.asiannet.com/ftp/2684/TD_M280_en_2011-03-29.pdf (accessed on January 4th, 2019).
56. Materialise. Materialise control platform. Availabe online: http://www.materialise.com/en/software/control-platform (accessed on January 7th, 2019).
57. Materialise. The Next Level of Quality Inspection with 3D Print Software. Availabe online: https://vimeo.com/187782860 (accessed on January 3rd, 2019).
58. Sigma_labs. PrintRite3D In-Process Control and Quality Assurance Software for Additive Manufacturing. Availabe online: https://sigmalabsinc.com/products/ (accessed on January 8th).
59. Betts, S.; Engineer, R.; Process, D. Evaluation of Quality Signatures™ using In Situ Process Control during Additive Manufacturing with Aluminum Alloy AlSi10 Mg. *Sigma Labs, Santa Fe, NM, Report No. BY6-2018-003. https://www. sigmalabsinc. com/sites/default/files/2018-04/In-Situ% 20Process% 20Mapping%*

20using% 20Thermal% 20Quality% 20Signatures% 20during% 20Additive% 20Manufacturing% 20with% 20Titanium% 20Alloy% 20Ti-6Al-4V. pdf **2017**.
60. Lim, G.; Lau, K.; Cheng, W.; Chiang, Z.; Krishnan, M.; Ardi, D. Residual Stresses in Ti-6Al-4V Parts Manufactured by Direct Metal Laser Sintering and Electron Beam Melting. *British Society of Strain Meaurement* **2017**.
61. Mercelis, P.; Kruth, J.-P. Residual stresses in selective laser sintering and selective laser melting. *Rapid prototyping journal* **2006**, *12*, 254-265.
62. Forging Industry Association. Heat treatment titanium alloys. Availabe online: https://www.forging.org/design/374-heat-treating-titanium-alloys (accessed on November 10th, 2019).
63. Hassan, M.H.; Diab, S.L. Visual inspection of products with geometrical quality characteristics of known tolerances. *Ain Shams Engineering Journal* **2010**, *1*, 79-84.
64. Salmi, M.; Paloheimo, K.-S.; Tuomi, J.; Wolff, J.; Mäkitie, A. Accuracy of medical models made by additive manufacturing (rapid manufacturing). *Journal of Cranio-Maxillofacial Surgery* **2013**, *41*, 603-609.
65. Ostra, T.; Alonso, U.; Veiga, F.; Ortiz, M.; Ramiro, P.; Alberdi, A. Analysis of the Machining Process of Inconel 718 Parts Manufactured by Laser Metal Deposition. *Materials* **2019**, *12*, 2159.
66. Carbone, V.; Carocci, M.; Savio, E.; Sansoni, G.; De Chiffre, L. Combination of a Vision System and a Coordinate Measuring Machine for the Reverse Engineering of Freeform Surfaces. *International Journal of Advanced Manufacturing Technology* **2001**, *17*, 263-271, doi:10.1007/s001700170179.
67. Metrology, L. Bridge CMM ALTERA series. Availabe online: http://www.lkmetrology.com/coordinate-measuring-machines/bridge-cmm/ (accessed on January 15th, 2019).
68. Li, Y.; Gu, P. Free-form surface inspection techniques state of the art review. *Computer-Aided Design* **2004**, *36*, 1395-1417.
69. Metrology, N. Nikon metrology solutions. Availabe online: https://www.nikonmetrology.com/images/brochures/nm-solutions-en.pdf (accessed on January 20th).
70. Van Gestel, N.; Cuypers, S.; Bleys, P.; Kruth, J.-P. A performance evaluation test for laser line scanners on CMMs. *Optics and lasers in engineering* **2009**, *47*, 336-342.
71. ASTM. Standard Test Method for Analysis of Titanium Alloys by X-Ray Fluorescence Spectrometry. ASTM International: West Conshohocken, PA, USA, 2011.
72. Gaddam, R.; Sefer, B.; Pederson, R.; Antti, M.-L. Study of alpha-case depth in Ti-6Al-2Sn-4Zr-2Mo and Ti-6Al-4V. In Proceedings of IOP Conference Series: Materials Science and Engineering, Luleå, Sweden, 21–22 March 2013; p. 012002.
73. Gammon, L.M.; Briggs, R.D.; Packard, J.M.; Batson, K.W.; Boyer, R.; Domby, C.W. Metallography and microstructures of titanium and its alloys. *ASM Handbook* **2004**, *9*, 899-917.
74. Greitemeier, D.; Palm, F.; Syassen, F.; Melz, T. Fatigue performance of additive manufactured TiAl6V4 using electron and laser beam melting. *International Journal of Fatigue* **2017**, *94*, 211-217, doi:10.1016/j.ijfatigue.2016.05.001.
75. Chen, Q.; Thouas, G.A. Metallic implant biomaterials. *Materials Science and Engineering: R: Reports* **2015**, *87*, 1-57.
76. Liu, X.; Chu, P.K.; Ding, C. Surface modification of titanium, titanium alloys, and related materials for biomedical applications. *Materials Science and Engineering: R: Reports* **2004**, *47*, 49-121.
77. Geetha, M.; Singh, A.; Asokamani, R.; Gogia, A. Ti based biomaterials, the ultimate choice for orthopaedic implants–a review. *Progress in Materials Science* **2009**, *54*, 397-425.
78. Rack, H.; Qazi, J. Titanium alloys for biomedical applications. *Materials Science and Engineering: C* **2006**, *26*, 1269-1277.
79. ASTM. Standard Guide for Preparation of Metallographic Specimens. ASTM International: West Conshohocken, PA, USA, 2017; Vol. E3 − 11.
80. ASTM. Standard Specification for Wrought Titanium-6Aluminum-4Vanadium Alloy for Surgical Implant Applications (UNS R56400)1. ASTM International: West Conshohocken, PA, USA, 2014.
81. ASTM. Standard Practice for Microetching Metals and Alloys. ASTM International: West Conshohocken, PA, USA, 2015; Vol. E407 − 07.
82. Toth, T.; Hudak, R.; Zivcak, J. Dimensional verification and quality control of implants produced by additive manufacturing. *Quality Innovation Prosperity* **2015**, *19*, 9-21, doi:10.12776/qip.v19i1.393.

83. Di Prima, M.; Coburn, J.; Hwang, D.; Kelly, J.; Khairuzzaman, A.; Ricles, L. Additively manufactured medical products – the FDA perspective. *3D Printing in Medicine* **2016**, *2*, 1-6, doi:10.1186/s41205-016-0005-9.
84. Kruth, J.-P.; Bartscher, M.; Carmignato, S.; Schmitt, R.; De Chiffre, L.; Weckenmann, A. Computed tomography for dimensional metrology. *CIRP Annals-Manufacturing Technology* **2011**, *60*, 821-842.
85. De Chiffre, L.; Carmignato, S.; Kruth, J.-P.; Schmitt, R.; Weckenmann, A. Industrial applications of computed tomography. *CIRP Annals-Manufacturing Technology* **2014**, *63*, 655-677.
86. Karatas, O.H.; Toy, E. Three-dimensional imaging techniques: A literature review. *European journal of dentistry* **2014**, *8*, 132.
87. Lantada, A.D.; Morgado, P.L. Rapid prototyping for biomedical engineering: current capabilities and challenges. *Annual review of biomedical engineering* **2012**, *14*, 73-96.
88. Nikon. XT H 225 for all-purpose X-ray and CT inspection. Availabe online: https://www.nikonmetrology.com/index.php?option=com_nikon&view=product&id=120&lang=en-gb (accessed on 30th of August, 2019).
89. Benedetti, M.; Cazzolli, M.; Fontanari, V.; Leoni, M. Fatigue limit of Ti6Al4V alloy produced by Selective Laser Sintering. *Procedia Structural Integrity* **2016**, *2*, 3158-3167.
90. Kahlin, M.; Ansell, H.; Moverare, J.J.; Konstruktionsmaterial; Linköpings, u.; Mekanik och, h.; Tekniska, f.; Institutionen för ekonomisk och industriell, u. Fatigue behaviour of notched additive manufactured Ti6Al4V with as-built surfaces. *International Journal of Fatigue* **2017**, *101*, 51-60, doi:10.1016/j.ijfatigue.2017.04.009.
91. De Nardo, L.; Altomare, L.; Del Curto, B.; Cigada, A.; Draghi, L. Electrochemical surface modifications of titanium and titanium alloys for biomedical applications. *Coatings for Biomedical Applications: Woodhead Publishing* **2012**, 106-142.
92. Ramsden, J.J.; Allen, D.M.; Stephenson, D.J.; Alcock, J.R.; Peggs, G.; Fuller, G.; Goch, G. The design and manufacture of biomedical surfaces. *CIRP Annals-Manufacturing Technology* **2007**, *56*, 687-711.
93. Kulkarni, M.; Mazare, A.; Schmuki, P.; Iglič, A. Biomaterial surface modification of titanium and titanium alloys for medical applications. *Nanomedicine* **2014**, *111*, 111.
94. Gulati, K.; Prideaux, M.; Kogawa, M.; Lima-Marques, L.; Atkins, G.J.; Findlay, D.M.; Losic, D. Anodized 3D–printed titanium implants with dual micro- and nano-scale topography promote interaction with human osteoblasts and osteocyte-like cells. *Journal of Tissue Engineering and Regenerative Medicine* **2017**, *11*, 3313-3325, doi:10.1002/term.2239.
95. Amin Yavari, S.; Loozen, L.; Paganelli, F.L.; Bakhshandeh, S.; Lietaert, K.; Groot, J.A.; Fluit, A.C.; Boel, C.H.E.; Alblas, J.; Vogely, H.C., et al. Antibacterial Behavior of Additively Manufactured Porous Titanium with Nanotubular Surfaces Releasing Silver Ions. *ACS applied materials & interfaces* **2016**, *8*, 17080.
96. Durakbasa, M.; Osanna, P.; Demircioglu, P. The factors affecting surface roughness measurements of the machined flat and spherical surface structures–The geometry and the precision of the surface. *Measurement* **2011**, *44*, 1986-1999.
97. Ghodrati, S.; Kandi, S.G.; Mohseni, M. Nondestructive, fast, and cost-effective image processing method for roughness measurement of randomly rough metallic surfaces. *JOSA A* **2018**, *35*, 998-1013.
98. Ehrenfest, D.M.D.; Coelho, P.G.; Kang, B.-S.; Sul, Y.-T.; Albrektsson, T. Classification of osseointegrated implant surfaces: materials, chemistry and topography. *Trends in biotechnology* **2010**, *28*, 198-206.
99. De Oliveira, R.; Albuquerque, D.; Cruz, T.; Yamaji, F.; Leite, F. Measurement of the nanoscale roughness by atomic force microscopy: basic principles and applications. In *Atomic force microscopy-imaging, measuring and manipulating surfaces at the atomic scale*, IntechOpen: London, UK, 2012.
100. Townsend, A.; Senin, N.; Blunt, L.; Leach, R.; Taylor, J. Surface texture metrology for metal additive manufacturing: a review. *Precision Engineering* **2016**, *46*, 34-47.
101. Barrere, F.; Mahmood, T.; De Groot, K.; Van Blitterswijk, C. Advanced biomaterials for skeletal tissue regeneration: Instructive and smart functions. *Materials Science and Engineering: R: Reports* **2008**, *59*, 38-71.
102. FDA. Guidance Document for Testing Orthopedic Implants With Modified Metallic Surfaces Apposing Bone Or Bone Cement. Branch, O.D., Ed. U.S. Food and Drug Administration: Rockville, Maryland, USA, 1994.

103. ISO. ISO/DIS 17327-1(en) Non-active surgical implants — Implant coating — Part 1: General requirements. Availabe online: https://www.iso.org/obp/ui#iso:std:iso:17327:-1:dis:ed-1:v1:en (accessed on January 24th).
104. Fox, J. Ergonomics contributions to subjective inspection in industrial quality control. *Production Engineer* **1975**, *54*, 111-114.
105. Megaw, E. Factors affecting visual inspection accuracy. *Applied ergonomics* **1979**, *10*, 27-32.
106. See, J.E. Visual inspection: a review of the literature. *Sandia Report SAND2012-8590, Sandia National Laboratories, Albuquerque, New Mexico* **2012**.
107. Jameeson, G. Inspection in the Telecommunications Industry: A Field Study of Age arid Other Performance Variables. *Ergonomics* **1966**, *9*, 297-303.
108. Lerouge, S. Introduction to sterilization: definitions and challenges. In *Sterilisation of Biomaterials and Medical Devices*, Elsevier: Sawston, UK, 2012; pp. 1-19.
109. Amann, B.; Bröcheler, P.; Carter, A.; Diedrich, D.; Fiedler, S.; Forster, A.; Jones, A.; Kamer, M.; Krüger, S.; Thomann, R., et al. *Reprocessing implants, supplied in an unsterile state, for orthopaedics and traumatology - Part 2*; mhp_verlag: Wiesbaden, Germany, 2015; Vol. 23, pp. 371-376.
110. ISO. Implants for surgery — Cleanliness of orthopedic implants — General requirements. International Organization for Standarization: Geneva, Switzerland, 2018; Vol. ISO 19227:2018.
111. Lerouge, S. Sterilisation and cleaning of metallic biomaterials. In *Metals for Biomedical Devices*, Elsevier: Sawston, UK, 2010; pp. 303-326.
112. Park, J.H.; Olivares-Navarrete, R.; Baier, R.E.; Meyer, A.E.; Tannenbaum, R.; Boyan, B.D.; Schwartz, Z. Effect of cleaning and sterilization on titanium implant surface properties and cellular response. *Acta biomaterialia* **2012**, *8*, 1966-1975.
113. Alfa, M.J.P.F. Monitoring and improving the effectiveness of cleaning medical and surgical devices. *AJIC: American Journal of Infection Control* **2013**, *41*, S56-S59, doi:10.1016/j.ajic.2012.12.006.
114. Govindaraj, S.; Muthuraman, M. *Systematic Review on Sterilization Methods of Implants and Medical Devices*; Sphinx Knowledge House: Pant Nagar, India, 2015; Vol. 8, pp. 974-4290.
115. Silindir, M.; Özer, A.Y. Sterilization methods and the comparison of E-beam sterilization with gamma radiation sterilization. *Fabad Journal of Pharmaceutical Sciences* **2009**, *34*, 43.
116. Mason, T.J. Ultrasonic cleaning: An historical perspective. *Ultrasonics sonochemistry* **2016**, *29*, 519-523.
117. Awad, S.B. High-power ultrasound in surface cleaning and decontamination. In *Ultrasound technologies for food and bioprocessing*, Springer: 2011; pp. 545-558.
118. Fuchs, F. Ultrasonic cleaning and washing of surfaces. In *Power Ultrasonics*, Elsevier: 2015; pp. 577-609.
119. Verhaagen, B.; Zanderink, T.; Rivas, D.F. Ultrasonic cleaning of 3D printed objects and Cleaning Challenge Devices. *Applied acoustics* **2016**, *103*, 172-181.
120. Lewis, S.; McIndoe, A.K. Cleaning, disinfection and sterilization of equipment. *Anaesthesia & Intensive Care Medicine* **2004**, *5*, 360-363.
121. Hoh, C.S.L.; Berry, D.P. Decontamination and sterilization. *Surgery (Oxford)* **2005**, *23*, 282-284, doi:10.1383/surg.2005.23.8.282.
122. Fakler, J.K.; Robinson, Y.; Heyde, C.E.; John, T. Errors in handling and manufacturing of orthopaedic implants: the tip of the iceberg of an unrecognized system problem? BioMed Central: 2007.
123. Kurtz, S.M.; Zagorski, M. Packaging and sterilization of UHMWPE. In *UHMWPE Biomaterials Handbook*, Elsevier: 2016; pp. 21-32.
124. ASTM. Standard Practice for Permanent Marking of Orthopaedic Implant Components. ASTM International: West Conshohocken, PA, USA, 2018; Vol. ASTM F983 - 86(2018)
125. Ogrodnik, P.; Moorcroft, C.; Wardle, P. The effects of laser marking and symbol etching on the fatigue life of medical devices. *Journal of medical engineering* **2013**, *2013*.
126. FierceBiotech. Stryker recalls 16,000+ orthopedic implants due to poor packaging integrity. Availabe online: https://www.fiercebiotech.com/medical-devices/stryker-recalls-16-000-orthopedic-implants-due-to-poor-packaging-integrity (accessed on February 19th).
127. FierceBiotech. Zimmer Biomet warns of 730K+ orthopedic implants with faulty packaging. Availabe online: https://www.fiercebiotech.com/medical-devices/zimmer-biomet-warns-730k-orthopedic-implants-faulty-packaging (accessed on February 19th).

128. Ratner, B.D.; Knovel. Sterilization of implants and devices. In *Biomaterials science: an introduction to materials in medicine*, 2nd ed.; Elsevier Academic Press: Amsterdam;Boston;, 2004; pp. 754-760.
129. ASTM. Standard Guide for Presentation of End User Labeling Information for Musculoskeletal Implants. ASTM International: West Conshohocken, PA, USA, 2014; Vol. F2943 - 14
